# ULK1-ATG13 and their mitotic phospho-regulation by CDK1 connect autophagy to cell cycle

**DOI:** 10.1371/journal.pbio.3000288

**Published:** 2020-06-09

**Authors:** Zhiyuan Li, Xiaofei Tian, Xinmiao Ji, Junjun Wang, Hanxiao Chen, Dongmei Wang, Xin Zhang

**Affiliations:** 1 High Magnetic Field Laboratory, Key Laboratory of High Magnetic Field and Ion Beam Physical Biology, Hefei Institutes of Physical Science, Chinese Academy of Sciences, Hefei, Anhui, P. R. China; 2 Institute of Physical Science and Information Technology, Anhui University, Hefei, Anhui, P. R. China; Institute of Basic Medical Sciences, NORWAY

## Abstract

Unc-51-like autophagy activating kinase 1 (ULK1)–autophagy-related 13 (ATG13) is the most upstream autophagy initiation complex that is phosphorylated by mammalian target-of-rapamycin complex 1 (mTORC1) and AMP-activated protein kinase (AMPK) to induce autophagy in asynchronous conditions. However, their phospho-regulation and functions in mitosis and cell cycle remain unknown. Here we show that ULK1-ATG13 complex is differentially regulated throughout the cell cycle, especially in mitosis, in which both ULK1 and ATG13 are highly phosphorylated by the key cell cycle machinery cyclin-dependent kinase 1 (CDK1)/cyclin B. Combining mass spectrometry and site-directed mutagenesis, we found that CDK1-induced ULK1-ATG13 phosphorylation promotes mitotic autophagy and cell cycle progression. Moreover, double knockout (DKO) of ULK1 and ATG13 could block cell cycle progression and significantly decrease cancer cell proliferation in cell line and mouse models. Our results not only bridge the mutual regulation between the core machinery of autophagy and mitosis but also illustrate the positive function of ULK1-ATG13 and their phosphorylation by CDK1 in mitotic autophagy regulation.

## Abstract 2

This study shows that the ULK1-ATG13 autophagy initiation complex is differentially regulated throughout the cell cycle, especially in mitosis, in which both ULK1 and ATG13 are highly phosphorylated by CDK1/cyclin B, promoting mitotic autophagy and cell cycle progression.

## Introduction

Autophagy occurs at basal levels in most tissues to selectively eliminate unwanted cellular components and can also be induced in response to various physiological and pathological conditions. Evolutionarily conserved autophagy-related (ATG) proteins play essential roles in autophagy nucleation, elongation, autophagosome closure, and maturation [1–3]. Unc-51-like autophagy activating kinase 1 (ULK1)/ATG1, a mammalian serine/threonine protein kinase, plays a key role in autophagy initiation [1]. It forms a complex with ATG13 and FAK family-interacting protein of 200 kDa (FIP200), which enhance ULK1 kinase activity and are vital for its localization and stability, mediating mammalian target-of-rapamycin (mTOR) signaling to autophagy [4–6]. On the other hand, many ATG proteins have also been proven to have vital physiological roles in other cellular processes in higher eukaryotes [2]. For example, emerging data show that some ATG proteins, such as ATG7, FIP200, Beclin-1, and ATG5, function in cell cycle and mitosis regulation [7–10]. However, whether ULK1, as the most upstream member of autophagy initiation complex, also participates in cell cycle and mitosis regulation has not been investigated.

Although autophagy has been extensively studied, currently established autophagy regulation mechanisms are mostly from asynchronous cells, in which only around 5% or less are in mitosis. However, recent studies suggest that autophagy is differentially regulated throughout the cell cycle [11–14], especially in mitosis [15,16]. Although the autophagosome number at a fixed time point is much reduced in mitotic cells compared with interphase cells [15], the autophagic flux is actually active [14,16,17]. Moreover, it has been reported that multiple kinases are involved in both autophagy and mitosis [11,13], indicating that these 2 cellular processes are intertwined. However, the mitotic autophagy regulation is still underexplored.

The only work that has investigated the molecular mechanism of mitotic autophagy regulation so far is by Furuya and colleagues [15]. They reported reduced phosphatidylinositol-3-phosphate (PtdIns3P) in mitosis, which suggested decreased VPS34 complex activity. They further identified VPS34-Thr159 as the mitotic-specific phosphorylation site by CDK1 (the mammalian homolog of Cdc2 in yeast), which is one of the cyclin-dependent kinases (CDKs) that coordinate with their cyclin partners to regulate cell cycle progression. This work has provided very important insights into the mitotic regulation of autophagy machinery. However, whether other molecular mechanisms are also involved in mitotic autophagy regulation is still unknown, especially the one that is responsible for the active autophagy flux maintenance in mitosis.

It is well known that ULK1-ATG13, the core machinery for the ULK1 autophagy initiation complex, was phospho-regulated primarily by mTOR and AMP-activated protein kinase (AMPK) to control autophagy induction in asynchronous cells [18–21]. However, its regulation mechanism and function in mitosis and cell cycle are still unknown. Here we found that ULK1-ATG13 not only plays essential roles in cell cycle progression but also is directly phosphorylated by CDK1/cyclin B in mitosis to regulate mitotic autophagy and cell cycle progression.

## Results

### The electrophoretic mobility shift of mitotic ULK1 is due to its phosphorylation

**Fig 1:**
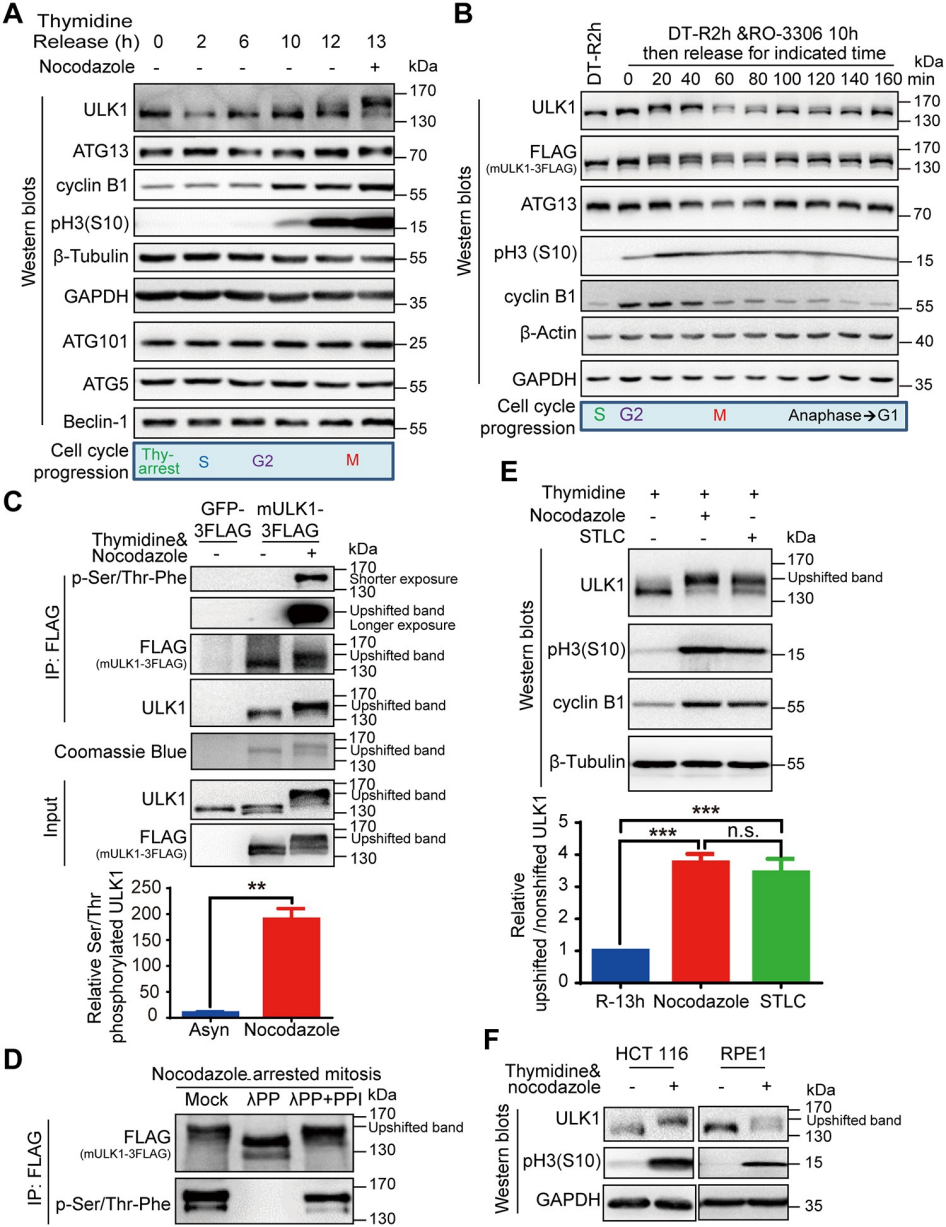
ULK1 undergoes electrophoretic mobility shift and is highly phosphorylated in mitosis. (A) ULK1-ATG13 shows a mobility shift in mitosis. HeLa cells synchronized by double-thymidine release in the presence or absence of nocodazole were subjected to SDS-PAGE and western blot analysis. (B) ULK1-ATG13 undergoes band shift during mitotic progression. HeLa cells synchronized by double-thymidine and RO-3306 were released into mitosis for western blot analysis. (C) ULK1 is phosphorylated in nocodazole-arrested mitosis. The 293T cells overexpressing FLAG-tagged mULK1 or GFP were synchronized by single-thymidine and nocodazole. The immunoprecipitates using the FLAG antibody were subjected to Coomassie Brilliant Blue R-250 staining and western blot analysis. Statistical analysis for relative serine/threonine phosphorylated ULK1 was shown in Fig 1C, lower panel. *n* = 4, **p < 0.01. (D) The immunoprecipitates from Fig 1C were treated with or without λPP in the presence or absence of PPIs and then subjected to western blot analysis. (E) ULK1 undergoes mobility shift in both nocodazole- and STLC-arrested mitosis. HeLa cells synchronized with single-thymidine and nocodazole or STLC were analyzed by western blot analysis. The upper panel shows the immunoblotting and the lower panel shows the ratio of upshifted and nonshifted ULK1. One-way ANOVA followed by Tukey’s multiple comparison test was used for the analysis. *n* = 5, ***p < 0.001. (F) ULK1 undergoes phosphorylation-induced electrophoretic mobility shift in single-thymidine and nocodazole synchronized mitotic HCT 116 and RPE1 cells analyzed by western blot analysis. Numerical data underlying the figure panels are available in S1 Data. Asyn, asynchronous; ATG, autophagy-related; DT-R2h, double-thymidine block and release for 2 hours; GAPDH, glyceraldehyde 3-phosphate dehydrogenase; GFP, green fluorescent protein; IP, immunoprecipitation; mULK1, mouse ULK1; n.s., not significant; HCT 116, human colorectal cancer cells; PPI, phosphatase inhibitor; RPE1, human retinal pigmented epithelial cells; STLC, S-trityl-L-cysteine; ULK1, unc-51-like autophagy activating kinase 1; λPP, lambda phosphatase.

To dissect the underlying mechanism of ULK1-ATG13 regulation during cell cycle and mitosis, we synchronized HeLa cells (human cervical cancer cells) using double-thymidine and nocodazole, a microtubule destabilizing reagent. Surprisingly, both ULK1 and ATG13 underwent a significant electrophoretic mobility shift in mitosis, whereas other ATGs such as ATG5, Beclin-1, or ATG101 did not (Fig 1A). In addition, we also used thymidine and a specific CDK1 inhibitor RO-3306 [22] for synchronization. Cells were treated with RO-3306 for 10 hours after thymidine release for 2 hours. Then, RO-3306 was washed out so that the cells can sequentially enter specific phases of mitosis. We found that the ULK1 and ATG13 electrophoretic mobility shifts were closely correlated with mitotic progression (Fig 1B).

We first examined ULK1 to see whether its electrophoretic mobility shift was due to its phosphorylation in mitosis by using immunoprecipitation from ULK1-expressing cell lines. Because mouse ULK1 (mULK1) has been validated in multiple studies in human cells [5,6,18,19,23], we constructed human embryonic kidney 293T (HEK-293T) cell lines overexpressing FLAG-tagged mULK1 (Q6PB82 in Uniprot, BC059835 in GenBank) or green fluorescent protein (GFP) control. The immunoprecipitated ULK1 shows an obvious band shift in mitosis both on the Coomassie brilliant blue–stained gel as well as on western blot (Fig 1C). Using a general anti-phosphoserine/threonine antibody [24,25], our results indicate that ULK1 is highly phosphorylated on serine/threonine in mitotic cells compared with asynchronous cells (Fig 1C). Then, we treated the ULK1 immunoprecipitation products with lambda phosphatase in the presence or absence of its inhibitors (Fig 1D). The FLAG-tagged mULK1 band was downshifted by lambda phosphatase treatment, which can be reversed by phosphatase inhibitors. The same pattern occurs with the phosphoserine/threonine antibody–detected band (Fig 1D). These results confirmed that the electrophoretic mobility upshift of ULK1 in mitosis is due to its phosphorylation.

To rule out the possibility that the band shift was caused by nocodazole-induced microtubule disruption, but not mitosis per se, we used a specific Eg5 inhibitor STLC [26] to synchronize HeLa cells to mitosis. We found that STLC could induce ULK1 band shift similar to nocodazole treatment, although to a lesser extent (Fig 1E). The dramatic band shift phenomena were also verified for endogenous ULK1 in human colorectal cancer cells (HCT 116) and human retinal pigmented epithelial cells (RPE1) (Fig 1F), as well as endogenous ULK1 and exogenous mULK1 in HEK-293T and HeLa cells (S1A and S1B Fig). Therefore, ULK1 is highly phosphorylated in mitosis of multiple cell types.

It should be mentioned that the original antibody we used for ULK1 (Cell signaling technology, #8054) did not detect the upshifted band but only showed decreased signal in mitosis (S1C Fig, the upper blot). However, it is interesting that when the polyvinylidene fluoride (PVDF) membrane was treated with lambda phosphatase to remove the phosphorylation, the upshifted band appeared (S1C Fig, the lower blot), which indicates that the mitotic ULK1 phosphorylation might interfere with the recognition of this specific antibody. Therefore, for all work other than indicated, we used another ULK1 antibody (Cell Signaling Technology, #4776) instead because it is consistent with the FLAG antibody recognition pattern (detecting the FLAG-tagged mULK1) as well as Coomassie staining in the FLAG-mULK1-expressing cells (Fig 1C).

### ULK1-ATG13 can be recognized by CDK substrate phosphorylation antibodies

**Fig 2:**
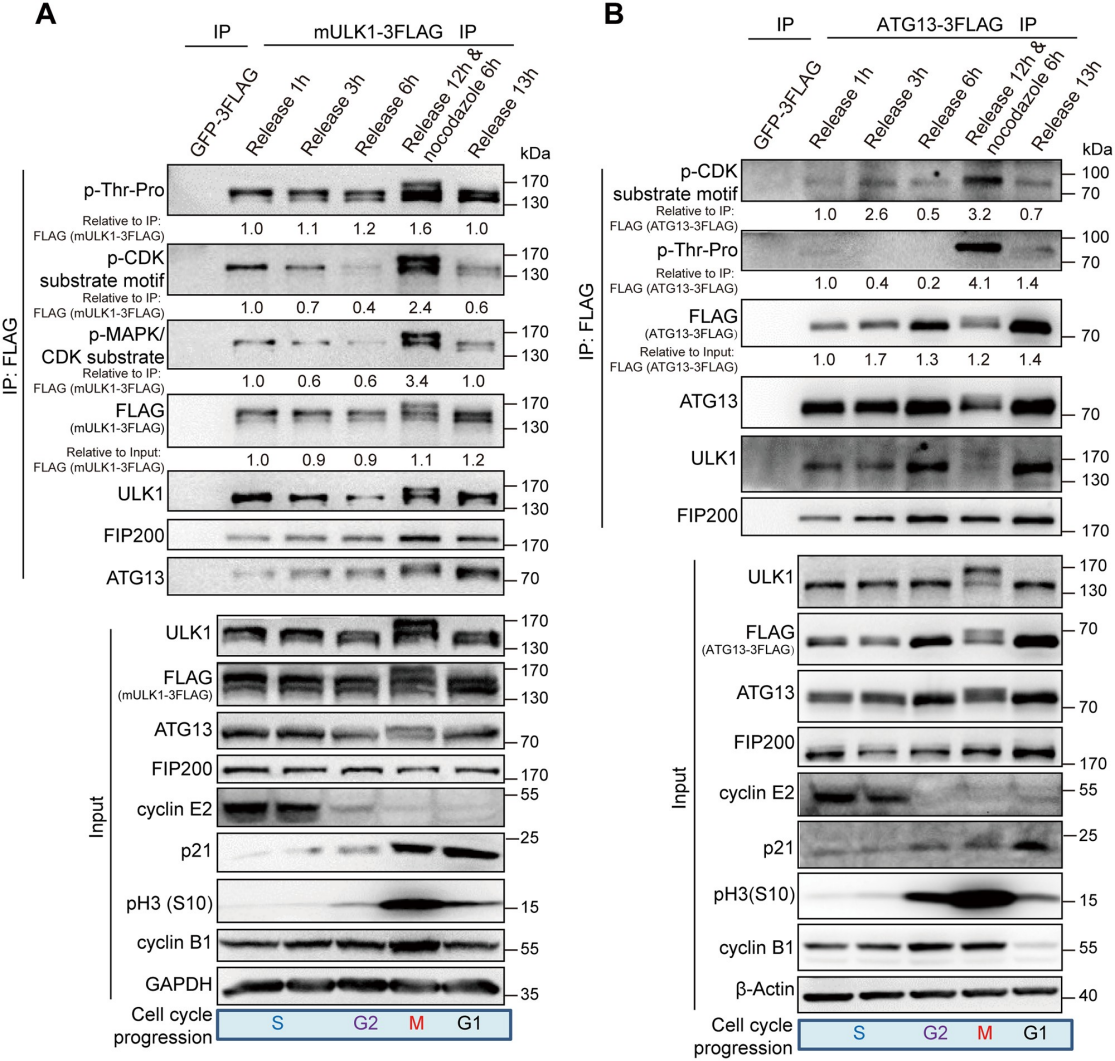
ULK1-ATG13 can be recognized by CDK substrate phosphorylation antibodies. (A-B) ULK1-ATG13 upshifted band in mitosis could be recognized by CDK substrate-specific antibodies. The 293T cells stably expressing FLAG-tagged mULK1 or ATG13 were synchronized by single-thymidine and released in the presence or absence of nocodazole. The coimmunoprecipitates and input were immunoblotted with specific antibodies. The phospho-signal was quantified relative to the FLAG antibody–detected bands (mULK1/ATG13-3FLAG) in IP; the IP signal of mULK1/ATG13-3FLAG was quantified relative to its input. Various cell cycle markers were detected to show the respective phases of cell cycle. ATG, autophagy-related; CDK, cyclin-dependent kinase; FIP200, FAK family-interacting protein of 200 kDa; GAPDH, glyceraldehyde 3-phosphate dehydrogenase; GFP, green fluorescent protein; IP, immunoprecipitation; mULK1, mouse ULK1; p-MAPK, phospho mitogen-activated protein kinase; ULK1, unc-51-like autophagy activating kinase 1.

Because phosphorylation-induced mobility shift is often indicative of phosphorylation on serine/threonine-proline residues [27], we first used Scansite 3 (Michael B. Yaffe Laboratory—Koch Institute, MIT) (http://scansite3.mit.edu/) to predict the potential kinases with proline-directed serine/threonine motif preference (the minimal consensus motif S/T-P, the optimal sequence S/T-P-X-R/K) [28,29], which revealed CDK1, CDK5, and mitogen-activated protein kinase 1/3 (MAPK1/3) as potential candidates for ULK1. We also constructed ATG13-overexpressing (O75143-2 in Uniprot, BC002378 in GenBank) cell line and performed immunoprecipitation in synchronized 293T cells overexpressing FLAG-tagged mULK1, ATG13, or GFP control (Fig 2A and 2B). Then, we examined the ULK1 or ATG13 immunoprecipitation products using motif antibodies for phospho mitogen-activated protein kinase (p-MAPK)/CDK substrate (PXS*P or S*PXR/K), p-CDK substrate [(K/H)pSP], and phospho-threonine-proline [30–32]. In fact, both the ULK1/FLAG antibodies and the CDK or MAPK/CDK substrate-specific phosphorylation antibodies could recognize a significant amount of ULK1 or ATG13 in both upshifted and nonshifted bands in mitotic cells compared with cells in other phases, which further indicates that ULK1-ATG13 was differentially regulated at both protein and phosphorylation levels during cell cycle (Fig 2A and 2B).

### ULK1 is a direct substrate of CDK1/cyclin B in mitosis

**Fig 3:**
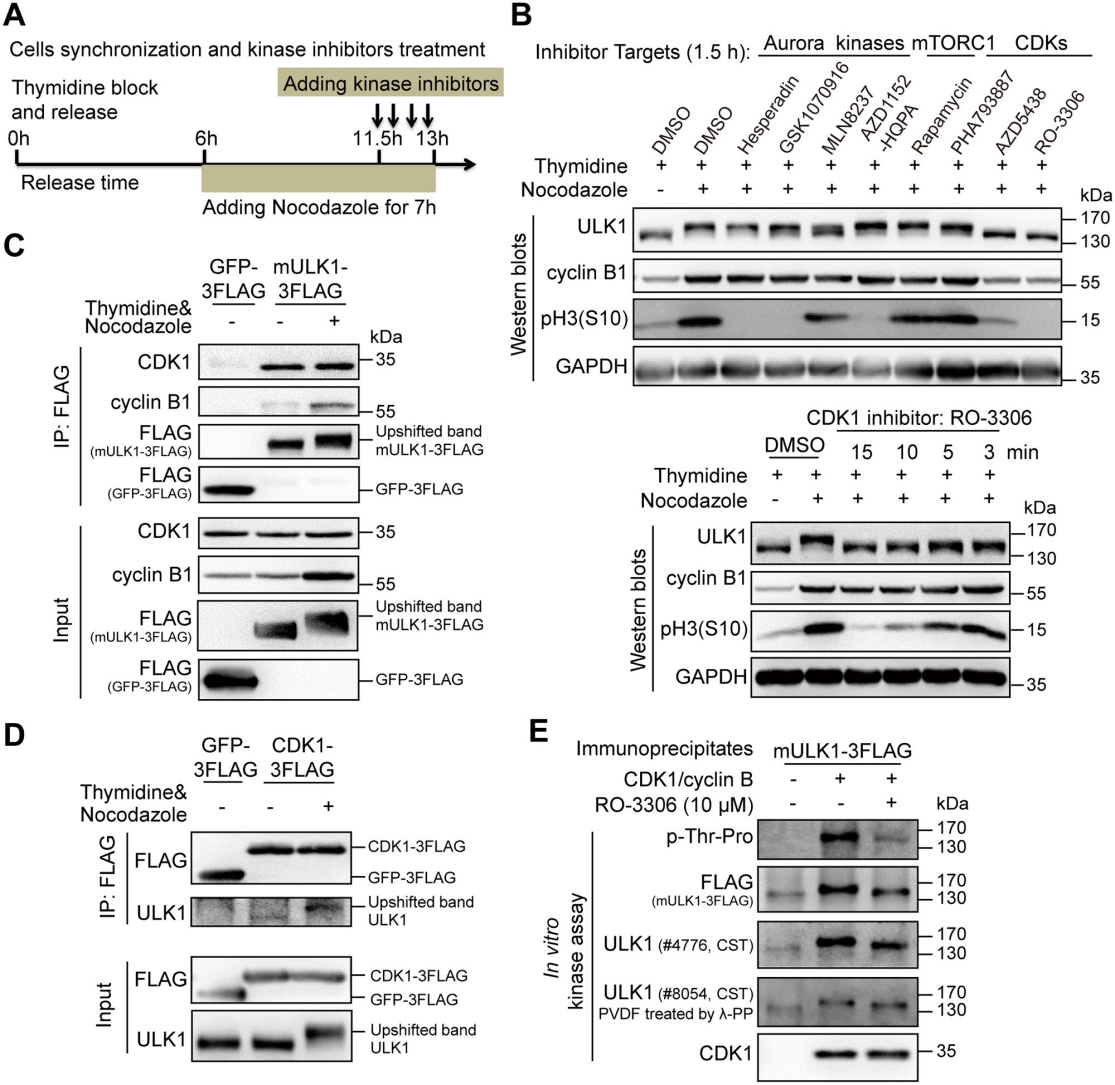
ULK1 is a substrate of CDK1/cyclin B in mitosis. (A) Illustration of cell synchronization and kinase inhibitors treatment. HeLa cells were synchronized with single-thymidine and nocodazole. Kinase inhibitors were added for different timepoints, ranging from 3 minutes to 1.5 hours. (B) CDK1 inhibitors, but not Aurora kinase, mTORC1, and other CDK inhibitors, abolished the ULK1 band shift in mitosis. HeLa cells synchronized and treated as (A) were subjected to western blot analysis. (C-D) ULK1 coimmunoprecipitates with CDK1 and vice versa. 293T cells stably overexpressing FLAG-tagged mULK1 or CDK1 were synchronized with single-thymidine and nocodazole and the coimmunoprecipitates were subjected to western blot analysis. (E) ULK1 is upshifted and phosphorylated by purified CDK1/cyclin B complex in vitro. Purified CDK1/cyclin B complex as kinase and the ULK1 immunoprecipitates from asynchronous 293T cells overexpressing FLAG-tagged mULK1 as substrate with or without RO-3306 were subjected to in vitro kinase assay and western blot analysis. CDK, cyclin-dependent kinases; CST, Cell Signaling Technology; GAPDH, glyceraldehyde 3-phosphate dehydrogenase; GFP, green fluorescent protein; IP, immunoprecipitation; mTORC1, mammalian target-of-rapamycin complex 1; mULK1, mouse ULK1; PVDF, polyvinylidene fluoride; ULK1, unc-51-like autophagy activating kinase 1; λ-PP, lambda phosphatase.

Because those aforementioned phospho-specific motif antibodies are not strictly specific, we hypothesized that the phospho-signal of ULK1 observed during thymidine release process is possibly due to the kinase activity of CDK1-cyclin B in mitosis and other CDKs or CDK1-cyclin A during S-G2 phase [33,34]. To find out the exact upstream kinase of ULK1 specifically in mitosis, we used various kinase inhibitors (Fig 3A), including multiple Aurora kinases inhibitors Hesperadin, GSK1070916, MLN8237, and AZD1152-HQPA [35–38]; mammalian-target-of rapamycin complex 1 (mTORC1) inhibitor rapamycin [39]; and CDKs inhibitors PHA793887, AZD5438, and RO-3306 [22,40,41], to examine their effects on mitotic ULK1 mobility shift (Fig 3B). Although none of the mTORC1, CDK2, CDK5, or CDK7 inhibitors could significantly reduce the ULK1 mobility shift, it was interesting that both CDK1 inhibitors AZD5438 and RO-3306 completely abolished the shift, whereas the CDK2/5/7 inhibitor PHA793887 did not (Fig 3B, the upper panel). In addition, although most Aurora kinases inhibitors have no effect, the Aurora A inhibitor MLN8237 moderately reduced ULK1 band shift. However, we found that the other 2 Aurora A inhibitors, MLN8054 and Aurora A inhibitor I, did not have such effect (S2A Fig). Moreover, because treating cells with Aurora or CDK1 inhibitors for 1.5 hours could induce loss of mitotic cells, which was shown by phospho histone H3 serine 10 [pH3(S10)], one of the most commonly used mitotic markers [42,43], or cyclin B1 reduction (Fig 3B and S2 Fig), the Aurora A inhibitor MLN8237-induced ULK1 mobility change is likely caused by cell cycle alteration. The fact that MLN8237 treatment for a shorter time (1 or 0.5 hours) did not cause ULK1 band shift change or cyclin B1 reduction (S2B Fig) further supports that Aurora A is less likely to be the upstream kinase for ULK1. However, when we treated the mitotic HeLa cells with RO-3306 for as short as 3 minutes, which did not reduce the pH3(S10) or cyclin B1 level (Fig 3B, the lower panel), we still observed the significant reduction of mitotic ULK1 mobility shift. Therefore, CDK1 is likely the major ULK1 upstream kinase that is responsible for its mobility shift in mitosis.

Next, we examined the in vivo association between ULK1 and CDK1 to further confirm their kinase–substrate relationship. ULK1 immunoprecipitation revealed that CDK1 and its mitotic partner cyclin B1 can be coimmunoprecipitated by ULK1 (Fig 3C). Reciprocally, we also established a FLAG-tagged CDK1–overexpressing 293T cell line and found that ULK1 could also be coimmunoprecipitated by the FLAG-CDK1 (Fig 3D), which further confirmed that CDK1 interacts with ULK1. Importantly, we also performed in vitro kinase assay using purified CDK1/cyclin B kinase complex and immunoprecipitates using FLAG antibody from 293T ULK1-knockout (KO) cells reconstituted with FLAG-mULK1. We found that ULK1 could be highly phosphorylated and upshifted by CDK1/cyclin B complex, which could be antagonized by the CDK1 inhibitor RO-3306 (Fig 3E). All these results confirm that ULK1 is a direct substrate of CDK1.

To further investigate ULK1 in mitosis, we constructed ULK1-KO HeLa and 293T cells using CRISPR/Cas9 (S3A and S3B Fig). Moreover, because ULK1 could be autophosphorylated, the kinase-dead ULK1-K46I mutant [19,44] was also investigated. Using HeLa ULK1-KO cells reconstituted with FLAG-ULK1-K46I, our results show that ULK1-K46I could undergo mobility shift and mitotic phosphorylation as well (S3C and S3D Fig). Consistent with the cellular experiments (S3C and S3D Fig), in vitro kinase assay also suggested that CDK1/cyclin B could phosphorylate ULK1-K46I and induce its band shift (S3E Fig). Therefore, the ULK1 phosphorylation in mitosis is independent of ULK1 kinase activity. Additionally, ULK2, a member of ULK1 kinase family, was also found to be phosphorylated in mitosis, which can be recognized by CDK substrate phosphorylation antibody and FLAG antibody (detecting FLAG-tagged mULK2, mULK2-3FLAG) (S4 Fig).

### ATG13 is also a direct substrate of CDK1/cyclin B in mitosis

**Fig 4:**
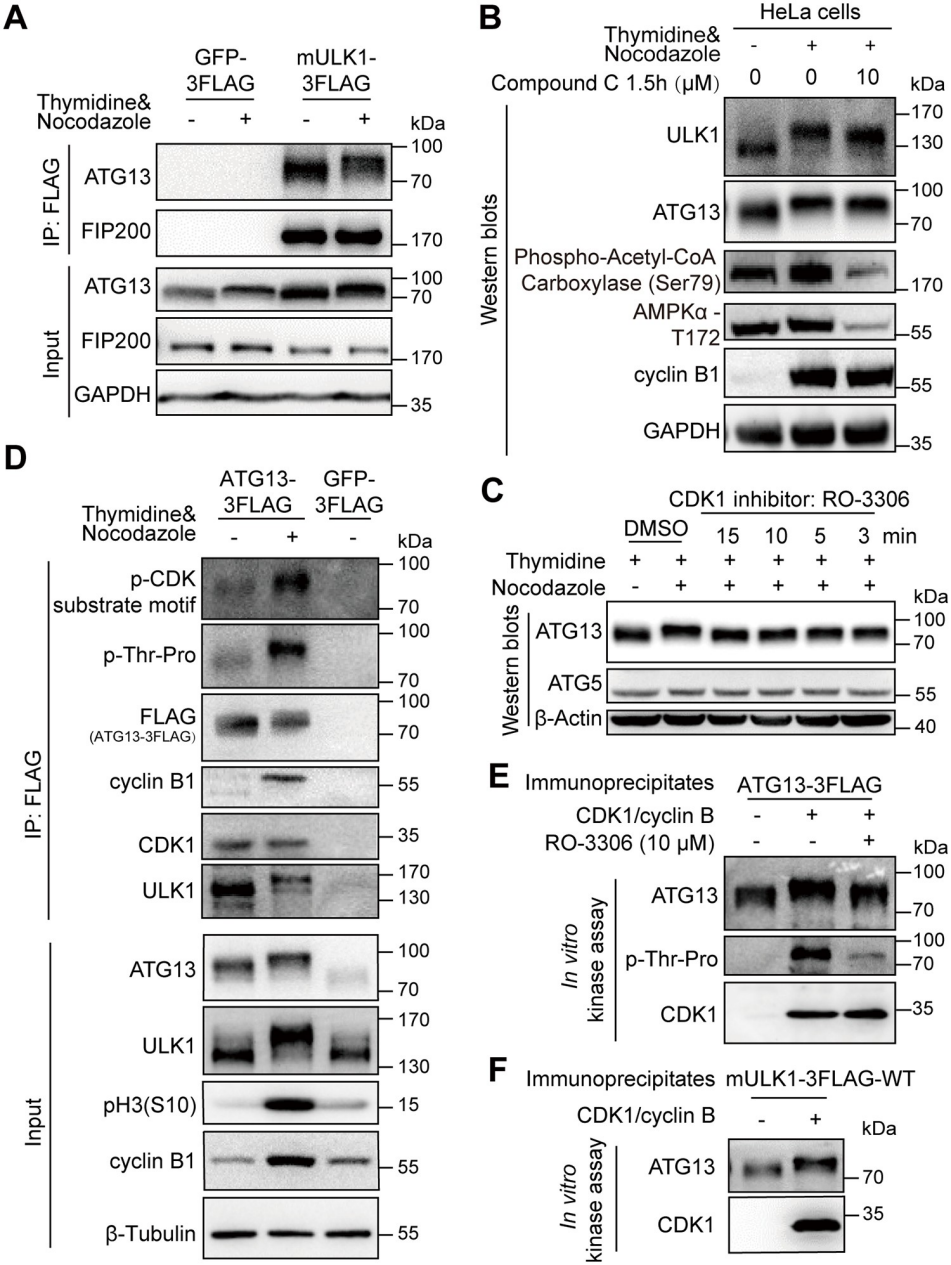
ATG13 is a substrate of CDK1/cyclin B in mitosis. (A) Mitotic ATG13 undergoes mobility upshift in mitosis in electrophoresis. The 293T cells overexpressing FLAG-tagged mULK1 or GFP were synchronized by single-thymidine in the presence or absence of nocodazole and coimmunoprecipitated by the FLAG antibody followed by western blot analysis. Size of endogenous ATG and expressed FLAG-tagged ATG13 were not distinguishable in electrophoresis here. (B) The effect of AMPK inhibition on ULK1 and ATG13 mobility shift in mitosis. The AMPK inhibitor Compound C was used to evaluate the role of AMPK on ULK1 or ATG13 mobility shift as Fig 3B. Phospho-AMPKα-T172 and phospho-Acetyl-CoA Carboxylase (Ser79) were used to indicate AMPK inhibition. (C) CDK1 inhibitor RO-3306 decreases the ATG13 band shift in mitosis. HeLa cells synchronized and treated as Fig 3A were subjected to western blot analysis. (D) ATG13 is phosphorylated and interacts with CDK1/cyclin B1 in mitosis. The 293T cells overexpressing FLAG-tagged ATG13 were synchronized by single-thymidine in the presence or absence of nocodazole. The coimmunoprecipitates by FLAG antibody were subjected to western blot analysis. (E-F) ATG13 is directly phosphorylated by purified CDK1/cyclin B complex in vitro. The ATG13 immunoprecipitates from asynchronous 293T cells overexpressing FLAG-tagged ATG13 or ULK1 and purified CDK1/cyclin B complex were subjected to in vitro kinase assay with or without RO-3306 followed by western blot analysis. (E) shows representative western blot analysis of the ATG13 immunoprecipitates as substrate, and (F) shows the representative western blot analysis of the ULK1-WT coimmunoprecipitates as substrate. AMPK, AMP-activated protein kinase; ATG, autophagy-related; CDK, cyclin-dependent kinase; FIP200, FAK family-interacting protein of 200; GAPDH, glyceraldehyde 3-phosphate dehydrogenase; GFP, green fluorescent protein; IP, immunoprecipitation; mULK1, mouse ULK1; ULK1, unc-51-like autophagy activating kinase 1; WT, wild type.

As mentioned earlier, ATG13 has the same electrophoretic mobility shift in mitosis as ULK1 does. However, another component of the ULK1 complex, the ULK1-interacting protein FIP200 [45], did not show mitotic mobility shift (Fig 4A). We also found that the upstream kinase of both ULK1 and ATG13 in interphase, AMPK, does not contribute to the mitotic mobility shift, as demonstrated by the results of an AMPK inhibitor Compound C [46] (Fig 4B). In contrast, similar to ULK1, ATG13 mobility shift in mitosis was decreased by the CDK1/cyclin B–specific inhibitor RO-3306 (Fig 4C and S5A Fig), which was further confirmed by coimmunoprecipitation (S5B Fig). These demonstrate that mitotic ATG13 is also phosphorylated by CDK1.

Next, we performed coimmunoprecipitation to examine the interaction between ATG13 and ULK1 or CDK1 in 293T cells overexpressing FLAG-ATG13. Phospho-antibodies of both proline -directed phosphoserine and threonine showed significant signal increase for the mitotic ATG13 (Fig 4D). The electrophoretic mobility shift was also apparent. Moreover, both CDK1 and cyclin B1 could be coimmunoprecipitated by ATG13 (Fig 4D), which confirmed the interactions among ATG13, ULK1, and CDK1/cyclin B1.

Because ATG13 is a substrate of ULK1 [47], it is possible that ULK1 phosphorylates ATG13 to regulate its mobility shift in mitosis. However, ULK1 kinase activity is dispensable for ATG13 mitotic mobility shift according to the data from kinase-dead ULK1-K46I-expressing cells (S3D Fig, the input and IP for ATG13). It should be mentioned that in mitosis, the extent of ATG13 band shift is indistinguishable between ULK1-KO and ULK1-overexpression cell lines but is abrogated by RO-3306 (S5C Fig, ATG13 blot of lane 1/2 and lane 3/4). This indicates that ATG13 phosphorylation-associated electrophoretic mobility shift in mitosis is dependent on CDK1/cyclin B kinase activity but not its well-known kinase ULK1, which is consistent with the ULK1-K46I data (S3D Fig). However, cells with ULK1-KO had downshifted ATG13 compared with cells exogenously expressing ULK1 (S5D Fig, ATG13 blot of lane 1/2 and lane 3/4) because ULK1 phosphorylates ATG13 in asynchronous conditions [47]. In fact, in vitro kinase assay demonstrates that ATG13 could be directly phosphorylated and upshifted by purified CDK1/cyclin B kinase complex, which is confirmed by the upshifted/phosphorylated ATG13 band using both ATG13 immunoprecipitates and ULK1 coimmunoprecipitates as substrates (Fig 4E and 4F).

### ULK1 and ATG13 phosphorylation sites in mitosis by CDK1/cyclin B

**Fig 5:**
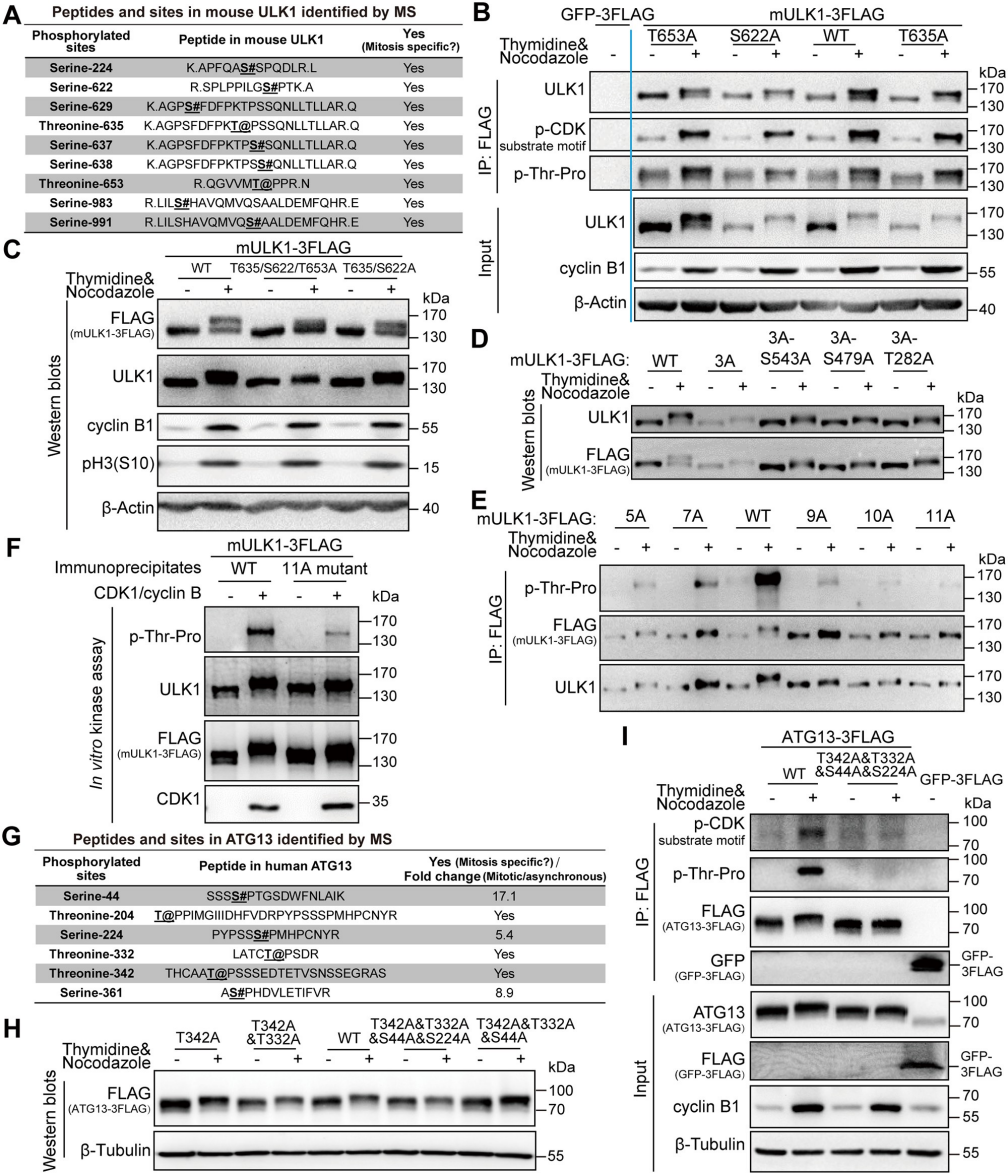
ULK1 and ATG13 phosphorylation sites in mitosis by CDK1/cyclin B. (A) The mitotic-specific phosphorylated sites identified by MS in mitotic mULK1 compared with asynchronous mULK1. (B-C) S622/T635/T653 phosphorylation contributes to ULK1 mobility shift in mitosis. The 293T cells overexpressing FLAG-tagged mULK1-S622/T635/T653 mutants were synchronized with single-thymidine and nocodazole. Then immunoprecipitation for single mutant with FLAG antibody (B) or western blot analysis for double and triple mutant (C) was performed. (D-E) More sites contribute to ULK1 band shift. Based on triple 3A mutant, the other 8 Ser/Thr sites were mutated into Ala. The 293T cells expressing various mutants were synchronized into mitosis with thymidine and nocodazole for immunoprecipitation by FLAG antibody and western blot analysis. (F) ULK1-11A mutant was not upshifted or phosphorylated by CDK1/cyclin B kinase complex in vitro. The ULK1-11A mutant immunoprecipitates from asynchronous 293T cells overexpressing FLAG-tagged mULK1 and purified CDK1/cyclin B complex were tested in an in vitro kinase assay and western blot analysis. (G) The phosphorylated sites identified by MS in mitotic ATG13 compared with asynchronous ATG13. (H-I) ATG13-T342/T332/S44/S224 phosphorylation contributes to ATG13 mobility shift in mitosis. HeLa-ATG13 KO cells (H) or 293T cells (I) overexpressing FLAG-tagged ATG13-T342/T332/S44/S224 mutants were synchronized with single-thymidine and nocodazole. Then western blot analysis for 4-site mutant (H) or immunoprecipitation and input for mutants with FLAG antibody (I) was performed. 3A, S622/T635/T653A; 5A, 3A-S479&S543A; 7A, 5A-S411&S413A; 9A, 5A-S413&T401&S403&S405A; 10A, 9A-T282A; 11A, 10A-T502A; ATG, autophagy-related; CDK, cyclin-dependent kinase; GFP, green fluorescent protein; IP, immunoprecipitation; MS, mass spectrometry; mULK1, mouse ULK1; ULK1, unc-51-like autophagy activating kinase 1; WT, wild type.

To identify the ULK1 phosphorylation sites in mitosis, we combined Scansite prediction and mass spectrometry analysis. We first selected 3 potential sites of mouse-derived ULK1, serine 622, threonine 635, and threonine 653, because they had high scores in Scansite prediction for proline-dependent serine/threonine kinase group (Pro_ST_kin), and their phosphorylation signals were also specifically identified in mitotic cells by mass spectrometry analysis (Fig 5A). Site-directed mutagenesis was used to mutate these sites from serine/threonine to unphosphorylatable alanine in FLAG-mULK1. Using 293T cells stably expressing FLAG-GFP as control, immunoprecipitation for FLAG-tagged mULK1-S622A/T635A/T653A mutants was conducted in both asynchronous and mitotic cells. Although none of the single mutations obviously disrupted ULK1 phosphorylation or band shift in mitosis (Fig 5B), S622A&T635A&T653A triple mutant significantly decreased the electrophoretic mobility shift compared with wild-type (WT) ULK1 (Fig 5C).

Because even the triple mutant did not completely abolish the band shift, we further mutated more predicted sites in the triple mutant (S622/T635/T653A [3A]) background. It shows that the 11A (S622&T635&T653&S479&S543&S413&T401&S403&S405&T282&T502A) mutant collapsed the electrophoretic mobility shift and abolished ULK1 phosphorylation (as indicated by the p-Thr-Pro signal in Fig 5E) in mitosis, whereas the other mutants, including the 3A-S479&S543A (5A), 5A-S411&S413A (7A), 5A-S413&T401&S403&S405A (9A), and 9A-T282A (10A), still have detectable phosphorylation signals and upshift in mitosis (Fig 5D and 5E). Importantly, in vitro kinase assay using purified CDK1/cyclin B proteins also showed that the extent of upshift for ULK1-11A is much less compared with ULK1-WT (Fig 5F). Therefore, besides the 3 major sites (S622A/T635A/T653A), CDK1/cyclin B phosphorylates multiple other sites of ULK1 in mitosis. In addition, using mass spectrometry and site-directed mutagenesis, specific phosphorylation sites of mouse ULK2 in mitosis were also preliminarily identified (S6A–S6C Fig).

As for ATG13, we also combined Scansite prediction and mass spectrometry analysis, which identified 4 sites, ATG13-T342/T332/S44/S224 (Fig 5G). To confirm the ATG13 phosphorylation sites in mitosis, we constructed their alanine mutants in ATG13-KO HeLa cell line (Fig 5H and S7 Fig). Site-directed mutagenesis, western blot analysis, and immunoprecipitation were combined for site verification (Fig 5H and 5I), and we found that the 4-site mutant significantly decreased the electrophoretic mobility shift and the phosphoserine/threonine-proline signals compared with WT ATG13 (Fig 5I).

It should be noted that the phosphorylation sites we identified in both ULK1 and ATG13 are conserved between different isoforms (S8A and S8B Fig). Moreover, because it is well established that mTOR and AMPK regulate autophagy through ULK1/ATG13 in asynchronous cells [18,19], we summarize the known phosphorylation sites of ULK1 and ATG13 (S9 Fig) and further examined whether their known fundamental sites of mTOR and AMPK [18–21] contributed to ULK1/ATG13 band shifts in mitosis. Our results show that ULK1-S757/S637/S555 did not obviously contribute to ULK1 band shift (S10A Fig). For ATG13, we observed some mobility shift changes caused by S224 mutation because S224 is a common site for both CDK1 and AMPK (S10B Fig).

### CDK1 phosphorylates ULK1-ATG13 to increase mitotic autophagy

**Fig 6:**
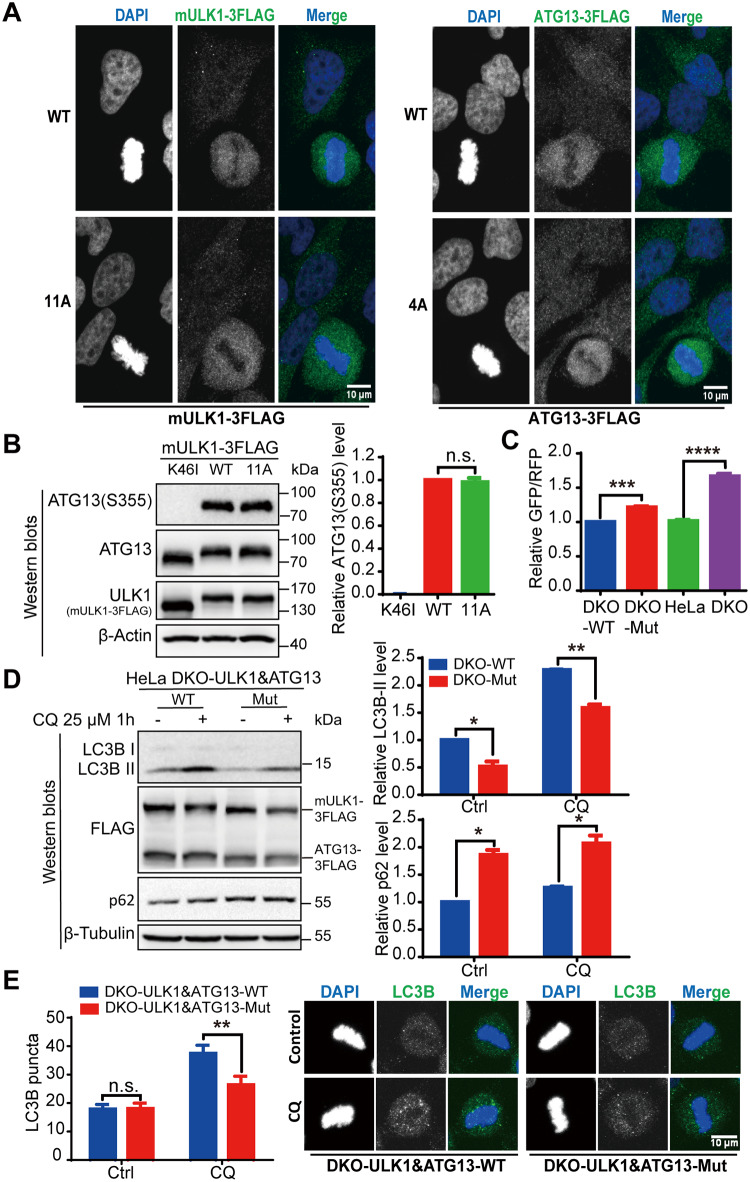
ULK1-ATG13 phosphorylation in mitosis promotes mitotic autophagy. (A) The localization of mutant ULK1-11A and ATG13-4A in mitosis. Cells released from thymidine block for 10 hours were fixed with 3.7% formaldehyde for 20 minutes at room temperature and then for blocking and FLAG antibody, Alexa-488 conjugated secondary antibody, DAPI staining. Scale bar, 10 μm. (B) The ULK1 kinase activity is not affected by the 11A mutation. ATG13-Ser355 is the substrate of ULK1, which corresponds to Ser318 of ATG13 isoform 2. ULK1-KO 293T cells reconstituted with ULK1-WT, ULK1-11A, or ULK1-K46I were plated and lysed with M-PER. The western blot for ATG13-Ser355 indicated that ULK1 activity was not affected by 11A mutation. *n* = 3. (C) Autophagy activity was examined in ULK1&ATG13 double WT or mutant (“Mut”) cells expressing GFP-LC3-RFP by flow cytometry. ULK1&ATG13 double WT or mutant cells, HeLa, or HeLa- ULK1&ATG13 DKO cells stably expressing GFP-LC3-RFP were established by infection of retrovirus packaged by pMRX-IP-GFP-LC3-RFP and helper plasmids Vsvg and pMLV. Mitotic cells were collected by shake-off from thymidine and nocodazole synchronized cells for flow cytometry detection. The GFP/RFP ratio inversely correlated with autophagy activity. *n* = 4, ***p < 0.001, ****p < 0.0001. (D) The autophagic flux of HeLa-DKO cells stably overexpressing double WT or mutant FLAG-tagged mULK1 and ATG13. Cells synchronized to mitosis with thymidine and nocodazole were shaken off and treated with or without autophagy inhibitor 25 μM CQ for 1 hour. The left panel is a representative western blot, and the right panel is the statistical result for LC3B-II and p62. *n* = 3, *p < 0.05, **p < 0.01. (E) LC3B puncta number was counted, and the micrographs were captured by Zeiss LSM710 confocal microscope. Scale bar, 10 μm. *n* = 43, **p < 0.01. Numerical data underlying the figure panels are available in S1 Data. 4A, T342/T332/S44/S224A; 11A, S622&T635&T653&S479&S543&S413&T401&S403&S405&T282&T502A; ATG, autophagy-related; CQ, chloroquine; Ctrl, control; DAPI, 4 DAPdiamidino-2-phenylindole; DKO, double knockout; GFP, green fluorescent protein; KO, knockout; M-PER, Mammalian Protein Extraction Reagent; mULK1, mouse ULK1; n.s., not significant; RFP, red fluorescent protein; ULK1, unc-51-like autophagy activating kinase 1; WT, wild type.

Although ULK1-ATG13 complex is a key autophagy regulator, its function has only been investigated in asynchronous cells. To unravel the function of these phosphorylation events that occur specifically in mitosis, we constructed ULK1 and ATG13 double WT or mutant cell line in HeLa ULK1&ATG13 double knockout (DKO) parental cell line (S11 Fig). We found that ULK1 or ATG13 was diffusely distributed in mitotic cells, which were not affected by ULK1/ATG13 alanine mutations (Fig 6A). Given that ATG13-Ser355 phosphorylation was used to evaluate ULK1 kinase activity [47], which is verified by the lack of phospho-signal in cells expressing kinase-dead K46I ULK1 (Fig 6B, lane 1), we found that the 11A mutation did not affect ULK1 kinase activity (Fig 6B, lanes 2 and 3).

Next, we examined the effects of these alanine mutations on autophagy. First, we constructed GFP-LC3-red fluorescent protein (RFP) stable cell lines in which the GFP/RFP signal ratio reciprocally correlates with autophagic activity [48]. According to both the relative GFP/RFP ratio and LC3-II turnover [3], the autophagy inhibition was confirmed for K46I kinase-dead mULK1 and ULK1-KO (S12A and S12B Fig), which is consistent with the known function of ULK1. Furthermore, we found that the autophagy activity was decreased in both ULK1&ATG13 double mutant cells and DKO cells (Fig 6C and S12C Fig). However, for ULK1 or ATG13 alone, the autophagy level was not affected in ULK1-11A mutant cells or only slightly decreased in ATG13-T342/T332/S44/S224A (4A) mutant cells (S12D and S12E Fig). Furthermore, we used autophagic flux to examine the amount of autophagic degradation to indicate autophagy activity. Autophagic flux inhibitors such as chloroquine (CQ) could cause autophagy marker LC3-II accumulation because of blocked LC3-II degradation [3]. We found that LC3B-II accumulation induced by CQ was decreased and p62 level was increased in ULK1&ATG13 mutant cell lines compared with the WT (Fig 6D). To get complementary information, LC3B puncta number was also counted by microscopy, which also showed that autophagy was decreased in ULK1&ATG13 mutant cells (Fig 6E). These results indicate that these alanine mutations of ULK1&ATG13 decreased mitotic autophagy activity.

We also observed that CDK1 inhibition by RO-3306 induced higher autophagic activity in both asynchronous and mitotic cells (S12F Fig). This is probably due to the fact that CDK1 has multiple substrates, and the RO-3306 inhibition results reflected the integrated effects of dephosphorylation of all the substrates on autophagy. For example, VPS34, AMPK, p62, and Raptor are all substrates of CDK1 [15,27,49,50] and also key autophagy regulators. Inhibition of CDK1 will release its inhibition on VPS34, which led to increased mitotic autophagic activity in a VPS34-dependent way [15]. In addition, RO-3306 could also inhibit other targets, including protein kinase C-delta (PKCδ) and serum/glucocorticoid-induced kinase (SGK), that inhibit autophagy [22,51,52], which could also lead to increased autophagic activity.

### ULK1-ATG13 is required for cell cycle progression

Although ULK1-KO cells had similar cell cycle distribution compared with WT HeLa cells in asynchronized cellular experiments (S13A Fig), the S/G2 transition in ULK1-KO cells was slightly delayed in synchronized experiment (S13B Fig). Given that the G2 and M phases are not distinguishable by propidium iodide (PI) staining alone, we further used pH3(S10) to examine whether ULK1 functions in G2/M transition. Although no differences were detected for the G2/M percentage in asynchronous WT and ULK1-KO cells, the mitotic progression is significantly inhibited in ULK1-KO cells synchronized with thymidine and nocodazole. It was shown by the percentage of pH3(S10) positive cells using flow cytometry (S14A Fig) or western blot analysis for cell cycle markers (S14B Fig). The antibody for p-CDK Substrate Motif [30] recognizes the substrate of CDK, whose phosphorylation level reflects the CDK activity and is used as a mitotic marker. Besides, either the upshifted band of myelin transcription factor 1 (Myt1) or the lower phosphorylation level of Cdc2-Y15 in mitosis [53] could also be used as cell cycle progression markers. Given that ULK1 is a serine/threonine protein kinase, the contribution of ULK1 kinase activity to mitotic entry was examined in cell lines expressing WT ULK1 or kinase-dead ULK1-K46I mutant. Our results show that the ULK1 kinase activity has little effect on mitotic entry (S14C Fig).

To further confirm the role of autophagy in cell cycle, ATG7-KO or ATG9A-KO cells were also constructed by CRISPR/Cas9. The mitotic entry delay was also detected in ATG7-KO or ATG9A-KO cells, which indicates that such effect is related to ATG7/ATG9A/ULK1-dependent autophagy but not ULK1 kinase activity–dependent autophagy (S14D and S14E Fig).

ATG13 was reported to function in mitotic catastrophe [54]. Cell cycle analysis indicated that ATG13-KO inhibited G2/M transition and decreased the mitotic index (S15A and S15B Fig), indicating that ATG13 is involved in cell cycle regulation. Although it has been reported that ATG13 is required for the kinase activity of ULK1 [6], but the ULK1 kinase activity is not necessary for mitotic progression (S14C Fig). Therefore, the phenotype of the ATG13-KO cells is likely due to the lack of ATG13 rather than the ULK1 kinase activity impairment.

**Fig 7:**
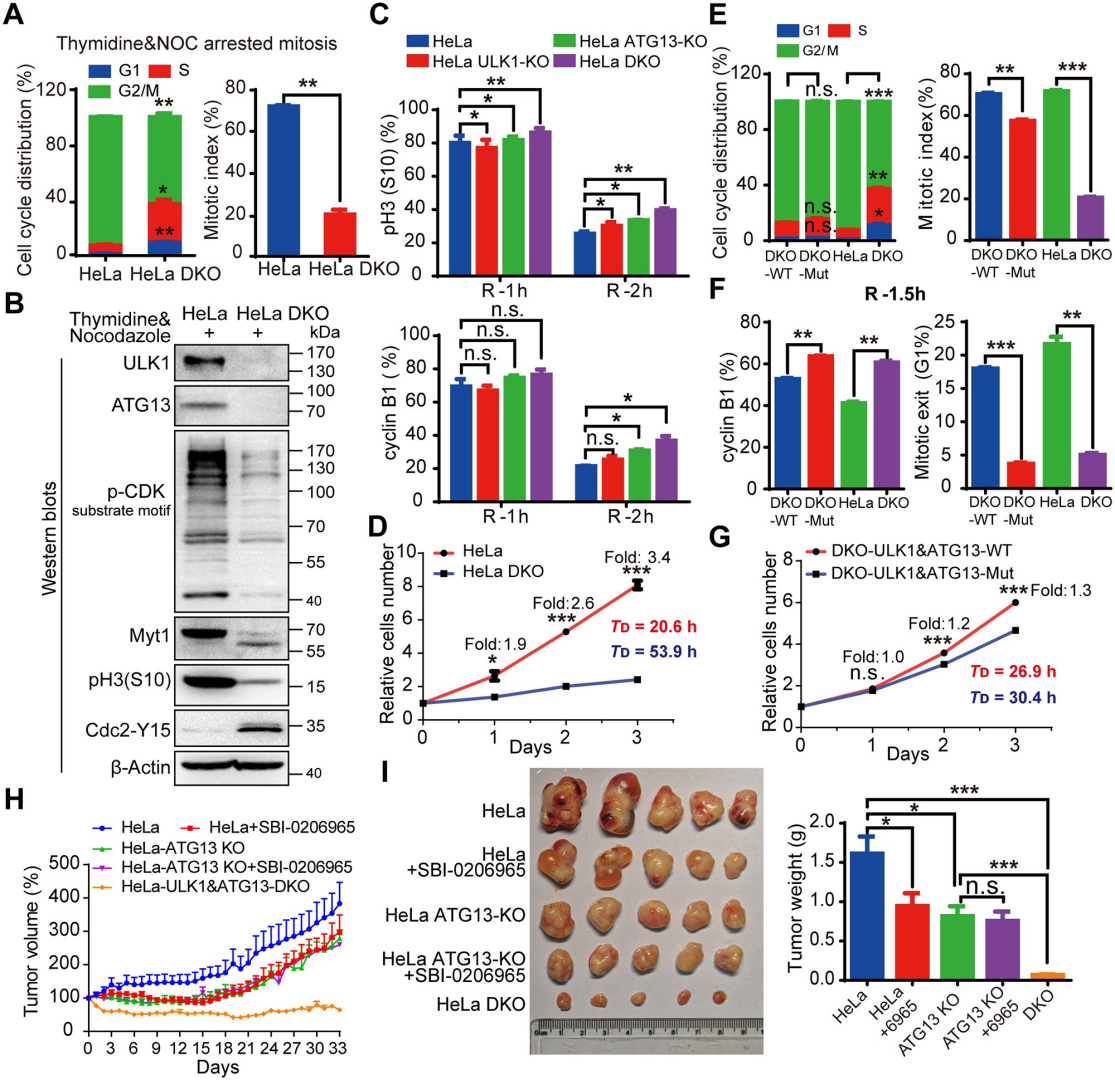
ULK1-ATG13 and their mitotic phospho-regulation by CDK1 are required for cell cycle progression. (A) ULK1-ATG13 DKO inhibits S/G2 and G2/M transitions. HeLa WT or ULK1&ATG13-DKO cells synchronized into mitosis were subjected to PI and pH3(S10) co-staining for cell cycle and mitotic index analysis by flow cytometry. *n* = 3, *p < 0.05, **p < 0.01. (B) Representative western blot analysis suggests that ULK1&ATG13 DKO inhibits mitotic entry, which is shown by mitotic markers and CDK1 substrate phosphorylation. (C) Mitotic exit of ULK1, ATG13, or ULK1&ATG13 KO cells. Cells were synchronized into mitosis with thymidine and NOC and released into NOC-free complete DMEM medium for different timepoints and then subjected to either PI and pH3(S10) co-staining or cyclin B1 staining for cell cycle, mitotic index, and cyclin B1 level analysis by flow cytometry. *n* = 3, *p < 0.05, **p < 0.01. (D) ULK1&ATG13 DKO inhibits cell proliferation. HeLa WT or ULK1&ATG13 DKO cells were plated at 1 × 10^5^ cells/mL and cultured for 1, 2, or 3 days. The cell number was counted by flow cytometry and the doubling time was calculated. *T*_D_ indicates the average cell doubling time and is calculated as: *T*_D_ = t_*_[lg2/(lgNt − lgN0)], where t is the culture time, Nt is the cell number after culturing, and N0 is the original cell number plated. *n* = 3, *p < 0.05, ***p < 0.001. (E-G) The role of ULK1-ATG13 phosphorylation in cell cycle progression and cell proliferation. ULK1&ATG13-DKO cells reconstituted with WT mULK1-3FLAG and ATG13-3FLAG or mutant (“Mut”) mULK1-11A and ATG13-4A, HeLa, or ULK1&ATG13-DKO cells were treated as (A)/(B) and (D). The mitotic exit (G1%) is the percentage of cells in G1 phase when released for indicated time. *n* = 3, **p < 0.01, ***p < 0.001. (H) The relative tumor volume growth curve of nude mice bearing different tumors with or without SBI-0206965. The nude mice were injected with cells (1 × 10^7^) in 100 μL PBS/Matrigel Matrix (1:1). Seven days postimplantation, 5 mice in each group were injected with 0.5% (M/V) methyl cellulose or SBI-0206965 in 0.5% methyl cellulose (20 mg/kg/d) every day for 33 days. Tumor growth was evaluated every day and tumor volume was calculated as: volume = 1/2 (length × width^2^). (I) Tumor growth in nude mice bearing WT or KO cells with or without ULK1 kinase inhibitor SBI-0206965. The protocols were indicated as Fig 7H and the mice were sacrificed, and tumors were harvested and weighed up at the end of the experiment. *n* = 5, *p < 0.05, ***p < 0.001. Numerical data underlying the figure panels are available in S1 Data. 4A, T342/T332/S44/S224A; 11A, S622&T635&T653&S479&S543&S413&T401&S403&S405&T282&T502A; ATG, autophagy-related; CDK, cyclin-dependent kinase; DKO, double knockout; DMEM, Dulbecco’s Modified Eagle Medium; KO, knockout; mULK1, mouse ULK1; Myt1, myelin transcription factor 1; NOC, nocodazole; n.s., not significant; pH3(S10), phospho histone H3 serine 10; PI, propidium iodide; ULK1, unc-51-like autophagy activating kinase 1; WT, wild type.

To further investigate the role of ATG13 and ULK1 in cell cycle regulation, we combined ULK1-KO with ATG13 guide RNA (gRNA) transient transfection and found that the cell cycle progression was inhibited (S15C and S15D Fig). Further, ULK1&ATG13-DKO cell line showed significant delays in cell cycle progression (Fig 7A and S15E Fig) compared with ULK1 or ATG13 KO alone (S14A and S15A Figs). The immunoblotting of cell cycle markers verified the mitotic index and cell cycle distribution data (Fig 7B). To rule out the off-target effect, rescue assays were performed in KO cells with exogenously expressed ULK1-ATG13 for mitotic entry and cell doubling time measurement, which confirmed the specificity of ULK1 and ATG13 KO (S16A and S16B Fig). Furthermore, DKO interfered with cyclin B1 and pH3(S10) decrease more significantly than ULK1 or ATG13 single KO (Fig 7C), and the growth rate of HeLa-DKO cells was significantly slowed down compared with HeLa cells (Fig 7D), which indicates that ULK1-ATG13 work together as a complex to regulate cell cycle.

Next, we investigated the role of ULK1-ATG13 phosphorylation in cell cycle progression. Although ULK1 and/or ATG13 mutant did not affect cell cycle distribution, the mitotic entry was delayed (Fig 7E, S17A and S17D Fig). Although ULK1 or ATG13 mutant alone did not obviously affect mitotic exit (S17B and S17E Fig), ULK1-ATG13 double mutant could significantly decrease mitotic exit (Fig 7F). The cell proliferation was decreased by ULK1-ATG13 double mutant but not ULK1 or ATG13 mutant alone (Fig 7G, S17C and S17F Fig). These data also indicate that phosphorylated ULK1-ATG13 works together as a complex.

To further test the effect of ULK1-ATG13 in vivo, nude mice bearing ULK1 and ATG13 single or double KO cells were established (S18A and S18B Fig). The tumor weight and volume of DKO group were significantly lower than all the other groups (Fig 7H and 7I). In contrast, ULK1 inhibitor SBI-0206965 [55] and/or ATG13-KO alone were not strong enough to inhibit tumor growth as efficiently as DKO (Fig 7H and 7I), indicating that targeting ULK1 and ATG13 together might be a potential anticancer strategy.

## Discussion

ULK1-ATG13 complex is mainly phosphorylated by AMPK and mTORC1 in asynchronous conditions [18–21], but little was known about its regulation in mitosis. Here we found that the master cell cycle kinase CDK1 phosphorylates ULK1-ATG13 complex to regulate its function in autophagy and cell cycle. Besides their known function in autophagy, we also found that ULK1 and ATG13 coordinate to orchestrate cell cycle progression in both cell line and mouse models.

### Kinases involved in both autophagy and mitosis

Recent literature indicates that there are some kinases that are involved in both autophagy and mitosis, which could bridge the autophagy and cell cycle regulation. Cell cycle kinases such as CDK1, Aurora A, and PLK-1 were found to regulate autophagy, whereas kinases originally found in autophagy control were shown to regulate mitosis as well, such as mTORC1 and AMPK [11,13]. In addition, increasing evidence shows that some other autophagic proteins such as Beclin-1, sequestosome 1 (SQSTM1)/p62, and gamma-aminobutyric acid receptor-associated protein (GABARAP) are related to mitotic events [10,50,56,57]. Although it has been reported that ULK3 (a member of ULK1 kinase family) could regulate cytokinesis and ATG13 could regulate colchicinamide-induced mitotic catastrophe [54,58], the roles of autophagy kinase complex ULK1-ATG13 in mitotic regulation were still unclear. Although previous reports implicated possible links between ULK1 and CDK1 [59,60], our study here is the first report that demonstrates CDK1 phosphorylates ULK1-ATG13 to promote mitotic autophagy and cell cycle progression.

Although our results show that ULK1 and ATG13 function downstream of CDK1 as kinase and substrates, the data about ULK1-ATG13 DKO affecting cell cycle progression and the signal changes of “p-CDK substrate motif” in ULK1-ATG13 DKO cells raised the possibility that ULK1 and ATG13 might function upstream of CDK1. However, we found that the kinase-dead mutant of ULK1-K46I did not affect cell cycle, which indicates that ULK1 kinase activity is unlikely to affect CDK1 to influence cell cycle. The signal change of “p-CDK substrate motif” in ULK1-ATG13 DKO cells is possibly due to the cell cycle change, which has been confirmed by various mitotic markers such as pH3(S10), p-CDK substrate motif recognition, upshifted band of Myt1, and phosphorylation level of Cdc2-Y15. But we cannot exclude the possibility that ULK1-ATG13 could function upstream of CDK1 in a kinase activity–independent way, which remains to be investigated.

### VPS34- and ULK1-ATG13 complex-dependent mitotic autophagy regulation

Although increasing evidence indicates that autophagy remains active in mitosis, the regulation mechanism was still unclear. Yuan’s group showed that activated CDK1 phosphorylates VPS34-Thr159 to inhibit VPS34-dependent autophagy in mitosis [15]. Our finding here demonstrates that CDK1 phosphorylates ULK1-ATG13 in mitosis to promote ULK1-ATG13-dependent autophagy, which at least partially contributed to the active autophagy state in mitosis, as reported in our previous study [14]. Therefore, it is likely that multiple autophagy regulators, not limited to the VPS34 complex and ULK1-ATG13 complex, contribute to the mitotic autophagy regulation, which certainly needs further investigations.

In addition, by using ATG7- and ATG9A-KO cells, we found that autophagy is important for cell cycle progression in general. However, our data also show that ULK1-kinase activity–dependent autophagy is not essential for such effects, which implicates the dual roles of mammalian ULK1 as its homolog in *Saccharomyces cerevisiae*. Atg1 (the ULK1 homolog in *S*. *cerevisiae*) has kinase-dependent and kinase-independent dual roles in autophagy [61,62]. On the one hand, Atg1 protein itself plays a structural role in recruiting Atg proteins to the pre-autophagosomal structure (PAS), which does not require its kinase activity. On the other hand, Atg1 kinase activity is responsible for dissociation of Atg proteins from the PAS during autophagosome formation. The mammalian ULK1 may also have kinase activity–dependent and kinase activity–independent roles in autophagy. However, although ULK1 protein itself and its kinase activity are both important for autophagy, which are confirmed by decreased autophagic flux in ULK1-KO and ULK1-K46I cells using western blot and relative GFP/RFP level in GFP-LC3-RFP-expressing cells (S12A and S12B Fig), our cell cycle analysis indicates that the ULK1 kinase activity–dependent autophagy is dispensable for cell cycle regulation, whereas the structural role of ULK1 in autophagy is likely not.

### ULK1/ATG13 phosphorylation by mTOR, AMPK, and CDK1

We found that the fundamental sites of mTOR and AMPK (S9 Fig) did not significantly contribute to ULK1/ATG13 band shifts in mitosis except S224 of ATG13, which is a common site of AMPK and CDK1. Although ATG13-S259 does not influence the extent of ATG13 band shift in mitosis, it affects ATG13 electrophoretic mobility in asynchronous cells, indicating that S259 is an important site of ATG13 in asynchronous cells, which is consistent with the previous report [20].

Although it is well known that mTOR and AMPK phosphorylate ULK1/ATG13 in asynchronous cells [18,19], we found that mTOR or AMPK inhibition did not affect ULK1/ATG13 band shift in mitosis. In fact, it should be mentioned that although our preprint manuscript (BioRxiv on May 2019) was in revision in this journal, Odle and colleagues published a work also showing that CDK1 could phosphorylate ULK1 and ATG13 in mitosis [63]. They performed in vitro assays and identified phosphorylation sites using a short truncation of ULK1 or ATG13 and proposed that CDK1 could substitute mTOR to phosphorylate ULK1/ATG13. However, our data show that those phosphorylation sites did not contribute to the mitotic band shifts of ULK1/ATG13, and their physiological functions are worth investigation in the future.

In conclusion, we have revealed that CDK1/cyclin B could phosphorylate ULK1/ATG13 on multiple sites to cause a significant electrophoretic shift in mitosis. ULK1/ATG13 works as a complex to regulate mitotic autophagy and cell cycle progression, which provides molecular mechanisms not only for maintaining mitotic autophagy but also for linking autophagy to cell cycle regulation.

## Materials and methods

### Antibodies and reagents

The autophagy antibody sampler kit (#4445), ULK1 Antibody Sampler Kit (#8359), Autophagy Induction (ULK1 Complex) Antibody Sampler Kit (#46486), the cell cycle regulation antibody sampler kit II (#9870), Phospho-(Ser) Kinase Substrate Antibody Sampler Kit (#9615), Phospho-Threonine-Proline Mouse mAb (P-Thr-Pro-101) (#9391), anti-ULK1 (#4776) antibody, AMPK and ACC Antibody Sampler Kit (#9957), anti-p62 (#5114) antibody, anti-ATG13(S355) (#26839) antibody, anti-ATG7 (#8558) antibody, anti-ATG9A (#13509) antibody, the HRP-linked anti-rabbit, and anti-mouse IgG antibodies were all from Cell Signaling Technology. The anti-FLAG (F3165) antibody was acquired from Sigma and anti-β-Tubulin, anti-GAPDH, and anti-β-Actin antibodies from Beijing TransGen Biotech (Beijing, China). The plasmid pMRX-IP-GFP-LC3-RFP (#84573) was purchased from Addgene. The GlutaMAX supplement and puromycin dihydrochloride were from Gibco. The secondary fluorescently conjugated antibodies and anti-fade prolong Gold with DAPI were from Molecular Probes. Prestained Protein Ladder (26616) and M-PER buffer were from Thermo Pierce. RO-3306 and Thymidine were from Sigma. Compound C, Aurora A inhibitor I, nocodazole, and SBI-0206965 were from Selleckchem. MLN8054 was from MedChemExpress. Methyl cellulose (#69016260) was from Sinopharm Chemical Reagent Co. Matrigel Matrix (#354234) was from BD. Protease inhibitor and phosphatase inhibitor cocktails were from Roche and the PVDF membrane from Millipore.

### Cell culture and stable cell lines establishment

HeLa, HCT 116, RPE1, and HEK-293T cells were all cultured in DMEM medium (without L-Glutamine) supplemented with 10% FBS, 2 mM GlutaMAX, and 1% penicillin/streptomycin (P/S). The plasmid for pBobi-FLAG-mULK1 contains 1 FLAG tag, and the affinity for FLAG antibody was lower than ULK1 antibody. Therefore, in order to enhance its affinity to FLAG antibody, 3×FLAG was added to mULK1 C-terminus [5]. Stable cell lines were constructed as described previously [64]. HEK-293T or HeLa cells stably expressing mULK1-3×FLAG were maintained in DMEM complete medium containing 1 μg/mL puromycin.

### Autophagic probe GFP-LC3-RFP for autophagy activity detection

The GFP-LC3-RFP is inserted into the pMRX-IP vector [48] for retrovirus package with the helper plasmid Vsvg and pMLV. Cells were infected with retrovirus for stable cell line establishment as previously described [14]. For flow cytometry, cells were collected with trypsin treatment and placed on ice before GFP and RFP fluorescence intensity analysis. The relative GFP/RFP ratio is reciprocally correlated to the autophagy activity [48].

### Immunofluorescence

The procedure was performed as described before [14]. HeLa ULK1-KO cells stably expressing FLAG-tagged mutant ULK1-11A and HeLa ATG13-KO cells stably expressing FLAG-tagged mutant ATG13-4A were grown on coverslips and released from thymidine block for 11 hours. Then cells were fixed with 3.7% formaldehyde at room temperature for 20 minutes and subsequently subjected to immunofluorescence using FLAG antibody and Alexa-488 conjugated anti-mouse IgG. HeLa-DKO cells stably expressing WT or mutant ULK1-ATG13 were released from thymidine block for 11 hours and treated with 25 μM CQ for another 1 hour. Then cells were fixed with −20 °C methanol for 5 minutes and subsequently subjected to immunofluorescence using LC3B antibody and Alexa-488 conjugated anti-rabbit IgG. Finally, cells were stained with 300 nM DAPI at room temperature for 2 minutes and then mounted with anti-fade prolong Gold for microscopy. Images were taken using the Zeiss confocal microscope LSM710, and representative micrographs are shown.

### CRISPR/Cas9 technology

The gRNA targeted to human ULK1/ATG13 was designed with CRISPR Design (http://crispr.mit.edu), and the gRNA targeted to human ATG7/ATG9A was designed according to the literature [65,66]. The sequence (human ULK1: 5’-GCCCTTGAAGACCACCGCGA-3′; human ATG13: 5’-CACATGGACCTCCCGACTGC-3′; human ATG9A: 5’-CCGTTTCCAGAACTACATGG-3′; human ATG7: GCTGCCAGCTCGCTTAACAT) was selected and subcloned into PX458 vector. HeLa and HEK-293T cells were transiently transfected with PX458-ULK1/ATG13-gRNA with Fugene 6. The cells were diluted into 0.5 cell/100 μL at 96-well plate after 12 hours of transfection. Single cell in 96-well-plate was cultured in DMEM complete medium to form single-cell clone that was cultured in 24-well plate and subjected to immunoblotting analysis using ULK1-, ATG13-, ATG7-, or ATG9A-specific antibody. One single-cell clone that could not be detected with ULK1/ATG13/ATG7/ATG9A antibody was selected as ULK1/ATG13/ATG7/ATG9A-KO cell. ULK1 and ATG13 DKO cells were established based on ULK1-KO cells using PX458- ATG13-gRNA.

### Immunoprecipitation and western blot analysis

The procedure was instructed as previously [64]. Most immunoprecipitation experiments were conducted in HEK-293T-derived cell lines because of a higher expression level for the exogenous protein. Briefly, HEK-293T cells stably expressing GFP-3FLAG or mULK1-3FLAG were lysed with M-PER supplemented with protease inhibitors and phosphatase inhibitors and centrifuged at 4 °C 14,000*g* for 10 minutes. The supernatant mixed with preincubated Protein G Dynabeads and FLAG antibody at 4 °C for 12 hours and washed 3 times with lysis buffer. Then the immunoprecipitate was denatured in 1×SDS-PAGE buffer at 95 °C for 7 minutes and subjected to SDS-PAGE and immunoblotting or Coomassie brilliant blue staining.

### Cell cycle synchronization

Various cell synchronization methods are used in this paper, which have been used previously. Briefly, a double-thymidine (2.5 μM) block arrested cells in G1/S border and cells progress through S, G2, and M phase after release. A double-thymidine or single-thymidine block in combination with nocodazole (100 ng/mL) or STLC (5 μM) treatment arrested cells in prometaphase or prophase. A double-thymidine block in combination with RO-3306 (10 μM) treatment arrested cells in late G2 phase and progressed into mitosis after RO-3306 washout 3 times with prewarmed PBS.

### Cell cycle analysis by flow cytometry/FACS

Cells for cell cycle analysis were trypsinized with 0.25% Trypsin/EDTA. After washing with ice-cold PBS twice, cells were fixed with −20 °C 75% ethanol overnight and then stained with PI/RNase staining buffer (BD Pharmingen) for 15 minutes at room temperature and analyzed with flow cytometry (Beckman Coulter, Cytoflex). Alternatively, for mitotic index analysis, cells fixed were stained with phospho-Histone H3 (S10) at 1:1,600 for 2 hours at room temperature and washed twice before Alexa-488 conjugated anti-rabbit IgG staining. After washing twice, PI/RNase staining was conducted as described previously before flow cytometry analysis. The data were analyzed by ModFit LT 4.1 and Flow Jo 7.6 software.

### Lambda phosphatase treatment

IP products from mULK1-3FLAG-overexpressing HEK-293T cells were aliquoted and treated with reaction buffer, reaction buffer containing 1 μL (400 units) lambda phosphatase (P0753S, NEB), or reaction buffer containing 1 μL lambda phosphatase plus 1×phosphatase inhibitors cocktail (Roche) at 30 °C for 30 minutes with gentle shaking. Then the reaction products were denatured at 95 °C for 7 minutes and subjected to immunoblotting with FLAG or Serine/Threonine-specific antibody.

### Mass spectrometry

For mass spectrometry assays, immunoprecipitates using FLAG antibody from asynchronous or mitotic 293T cells expressing FLAG-tagged mULK1/ATG13/mULK2 were separated by SDS-PAGE, the gel was stained with Coomassie brilliant blue, and the FLAG-tagged mULK1/ATG13/mULK2 band in each lane was excised. Samples were subjected to mass spectrometry analysis for mULK1/ATG13/mULK2 phosphorylation by Core Facility Center for Life Sciences, University of Science and Technology of China, School of Life Science and Technology, ShanghaiTech University. The identified phosphorylation sites specific in mitotic cells or with higher intensity were selected as the candidates for ULK1/ATG13/mULK2 phosphorylation sites in mitosis.

### In vitro kinase assay

Anti-FLAG immunoprecipitates from asynchronous 293T cells overexpressing FLAG-tagged mULK1 (WT or K46I kinase-dead) or ATG13 were washed 3 times with M-PER and then resuspended in ice-cold kinase buffer (50 mM Tris-HCl [pH 7.5] at 25 °C, 10 mM MgCl_2_, 0.1 mM EDTA, 2 mM DTT, 0.01% Brij 35). The immunoprecipitates were then incubated with or without 270 ng purified CDK1/cyclin B (Life technologies, Part Number: PV3292, Lot Number: 1816161K) in 20-μL reaction mix (kinase buffer and 20 μM ATP) pretreated with or without 10 μM CDK1 inhibitor RO-3306 at 30 °C with constant shaking for 30 minutes. The reaction was quenched by mixing with 5 μL 5×SDS-sample buffer and boiling at 95 °C for 7 minutes.

### Mouse model

Four-week-old female BALB/c nude mice were purchased from Nanjing Biomedical Research Institute of Nanjing University (Nanjing, China). All mice were kept in an animal room under the specific-pathogen-free (SPF) condition. The mice were fed with sterilized food and autoclaved tap water freely. The protocol involving animals was approved by the ethical and humane committee of Hefei Institutes of Physical Science, Chinese Academy of Sciences and carried out strictly in accordance with the related regulations (Hefei, China). After 1 week, HeLa/HeLa-ATG13-KO/HeLa-ULK1&ATG13-DKO cells (1 × 10^7^) suspended in 100 μL PBS/Matrigel Matrix (1:1) were injected into the subcutaneous space on the right flank of BALB/c nude mice. The mice bearing HeLa/HeLa-ATG13-KO cells were randomly divided into 2 groups of 5 mice each 7 days postimplantation. Mice were intraperitoneally injected with 0.5% (M/V) methyl cellulose used as the vehicle solution or SBI-0206965 in 0.5% methyl cellulose (20 mg/kg/d) every day for 33 days. Tumor growth was evaluated every day and tumor volume was calculated as: volume = 1/2 (length × width^2^). At the end of the experiment, the mice were killed by cervical dislocation, and tumors were harvested and weighed up.

### Quantification and statistical analysis

ImageJ software was used to quantify the relative protein value for western blot band, and Graphpad prism 6 was used to analyze the data using Student *t* test for 2 groups. All data are shown as mean ± SEM. *p*-values < 0.05 were considered as statistically significant.

## Supporting information

S1 Data In separate sheets, the excel spreadsheet contains the numerical data and statistical analysis for Figs 1C, 1E, 6B, 6C, 6D, 6E, 7A, 7C, 7D, 7E, 7F, 7G, 7H and 7I; S12A, S12B, S12D, S12E, S12F, S13A, S13B, S14A, S14C, S14E, S15A, S15C, S15E, S16A, S16B, S17A, S17B, S17C, S17D, S17E, S17F and S18B Figs. (XLSX)

S1 Raw Images All the original western blot images. (PDF)

S1 Fig ULK1 is phosphorylated and upshifted in mitosis. (A-B) Both endogenous human and exogenous mouse ULK1 are upshifted in thymidine and nocodazole-arrested mitosis. 293T and HeLa cells with or without FLAG-tagged mULK1 overexpression were synchronized into mitosis by single-thymidine and nocodazole for western blot analysis. (C) ULK1 phosphorylation in mitosis interferes with ULK1 antibody recognition. The ULK1 antibody (Cell Signaling Technology, #8054) could not recognize the upshifted band for mitotic ULK1 but could recognize when the PVDF membrane was treated with lambda phosphatase for 1 hour. PVDF, polyvinylidene fluoride; ULK1, unc-51-like autophagy activating kinase 1.
(TIF)

S2 Fig Aurora A is less likely to be responsible for ULK1 band shift in mitosis. (A-B) HeLa cells synchronized and treated as Fig 3A were subjected to western blot analysis. However, we found that the other 2 Aurora A inhibitors, MLN8054 and Aurora A inhibitor I, did not affect ULK1 band shift (A). In addition, MLN8237 treatment for a shorter time (1 hour or 0.5 hours) did not cause ULK1 band shift change as 1.5-hour treatment (B). MLN8237, MLN8054, and Aurora A inhibitor I were Aurora A inhibitor. CDK, cyclin-dependent kinase; ULK1, unc-51-like autophagy activating kinase 1.
(TIF)

S3 Fig CDK1 regulates ULK1 phosphorylation independent of ULK1 kinase activity. (A-B) ULK1-KO cells were established. HeLa and 293T cells transiently transfected with the CRISPR/Cas9 plasmid subcloned gRNA for human ULK1 were screened by western blot analysis and the ULK1-KO clones were identified. The red circles indicate the ULK1-KO clones for the following assay. (C-D) K46I kinase-dead ULK1 also underwent significant electrophoretic mobility shift and phosphorylation in mitosis as WT ULK1. HeLa ULK1-KO cells reconstituted with FLAG-tagged mULK1-K46I were treated as Figs 1C and 3B (the lower panel) and then analyzed by western blot analysis and immunoprecipitation, respectively. (E) In vitro kinase assay indicated that purified CDK1/cyclin B could induce K46I kinase-dead ULK1 to undergo significant electrophoretic mobility shift and phosphorylation. CDK, cyclin-dependent kinase; gRNA, guide RNA; KO, knockout; mULK1, mouse ULK1; ULK1, unc-51-like autophagy activating kinase 1; WT, wild type.
(TIF)

S4 Fig ULK2 is also phosphorylated and upshifted in mitosis. The 293T cells transiently transfected with FLAG-tagged mULK2 treated as Fig 3A were analyzed by western blot analysis and immunoprecipitation. mULK2, mouse ULK2; ULK2, unc-51-like autophagy activating kinase 2.
(TIF)

S5 Fig ATG13 is upshifted in mitosis. (A) HeLa cells were treated as Fig 4C for ATG13 mobility shift analysis. (B) ATG13 mobility shift in mitosis is decreased by CDK1 inhibitor RO-3306, similarly to ULK1, although to a lesser extent. The 293T cells overexpressing FLAG-tagged mULK1 were synchronized by single-thymidine in the presence or absence of nocodazole, treated with 10 μM RO-3306 for 5 or 30 minutes. The coimmunoprecipitate by FLAG antibody was subjected to immunoblotting with ATG13, FIP200 antibodies. (C-D) ULK1 expression level does not affect ATG13 mobility shift in mitosis. HeLa ULK1-KO cells with or without FLAG-tagged mULK1 expression synchronized into mitosis with thymidine and nocodazole (C) or in asynchronous condition (D) were treated with 10 μM RO-3306 for 30 minutes for western blot analysis. ATG, autophagy-related; CDK, cyclin-dependent kinase; FIP200, FAK family-interacting protein of 200 kDa; KO, knockout; mULK1, mouse ULK1; ULK1, unc-51-like autophagy activating kinase 1.
(TIF)

S6 Fig Identification of ULK2 phosphorylation sites in mitosis. (A-B) The preliminary identification of ULK2 phosphorylation sites in mitosis. The immunoprecipitate with FLAG antibody in asynchronous or mitotic 293T cells transfected with mULK2-3FLAG was subjected to SDS-PAGE and Coomassie brilliant blue staining (A) and mass spectrometry analysis of phosphorylation sites for mitotic mULK2 compared with asynchronous mULK2 (B). (C) The contribution of the potential residues to mitotic ULK2 band shift. HeLa cells were transfected with the mutant mULK2-3FLAG plasmid in indicated sites and analyzed by cell cycle synchronization and western blot. mULK2, mouse ULK2; ULK2, unc-51-like autophagy activating kinase 2.
(TIF)

S7 Fig ATG13-KO cells establishment. HeLa cells were transiently transfected with the CRISPR/Cas9 plasmid that subcloned gRNA for human ATG13. The ATG13-KO clones were screened by western blot and identified. The red circle indicates the ATG13-KO clones for the following assay. ATG, autophagy-related; gRNA, guide RNA; KO, knockout.
(TIF)

S8 Fig The phosphorylation sites we identified in both ULK1 and ATG13 are conserved between different isoforms. (A) The difference between the construct we used in this paper (Q6PB82 in Uniprot, BC059835 in GenBank) with the ULK1 used in some other studies (O70405 in Uniprot) is that there are 6 additional amino acids at position 507–512 (ATLFLP) of Q6PB82 and a conversion from S to T at position 469 of Q6PB82. (B) The human ATG13 cloned from the cDNA of HeLa cells, isofom2 (O75143-2 in Uniprot, BC002378 in GenBank), is used in this paper. It differs from O75143 (isoform 1) used in other studies with the missing region in the amino acids at position 263–299. The phosphorylation sites we identified in both ULK1 and ATG13 are conserved between different isoforms (A-B), which were aligned in Uniprot. ATG, autophagy-related; ULK1, unc-51-like autophagy activating kinase 1.
(TIF)

S9 Fig ULK1/ATG13 identified phosphorylation sites by kinases are shown as human/mouse. The identified phosphorylated residues of the mouse ULK1 and human ATG13 from “current study” and literature [18,20,21,47,63,67–70] are summarized. ATG, autophagy-related; ULK1, unc-51-like autophagy activating kinase 1.
(TIF)

S10 Fig The effects of ULK1-ATG13 sites at mTOR and AMPK on mitotic mobility shift. (A-B) The alanine mutants of ULK1 and ATG13 at fundamental mTOR/AMPK phosphorylation sites were constructed in ATG13-KO or ULK1-KO cells and examined their contribution to mitotic ULK1 (A) or ATG13 (B) band shift in mitosis. AMPK, AMP-activated protein kinase; KO, knockout; mTOR, mammalian target-of-rapamycin; ULK1, unc-51-like autophagy activating kinase 1.
(TIF)

S11 Fig Establishment of ULK1 and ATG13 DKO, double WT or mutant cell line. (A) ULK1 and ATG13 DKO cells establishment. HeLa ULK1-KO cells were transiently transfected with the CRISPR/Cas9 plasmid that subcloned gRNA for human ATG13. The ATG13-KO clones were screened by western blot analysis and identified as ULK1 and ATG13 DKO cells. The red circles indicate the ULK1 and ATG13 DKO clones for the following assays. (B-C) The ULK1 and ATG13 double WT or mutant cell lines were established by ATG13-WT/4A MSCV infection based on HeLa-DKO cell reconstituted with FLAG-tagged WT or 11A mutant mULK1. (D) The expression of ATG13 in ULK1 and ATG13 double WT or mutant cell lines and HeLa cells. 11A, S622&T635&T653&S479&S543&S413&T401&S403&S405&T282&T502A; ATG, autophagy-related; DKO, double knockout; gRNA, guide RNA; KO, knockout; MSCV, murine stem cell virus; mULK1, mouse ULK1; ULK1, unc-51-like autophagy activating kinase 1; WT, wild type.
(TIF)

S12 Fig The effects of ATG13 or ULK1 mutant, ULK1-ATG13 DKO, CDK1 inhibition on autophagy. (A-B) The autophagy inhibition was compared between FLAG-tagged wild-type and K46I kinase dead mULK1, HeLa and HeLa ULK1-KO, respectively. Cells were treated with EBSS starvation for 1.5 hours with or without Baf A1 and examined by western blot. GFP-LC3-RFP was stably expressed in indicated cell lines. The autophagy activity was detected by flow cytometry for GFP and RFP fluorescence intensity. (C) The autophagic activity of HeLa and HeLa-DKO cells in mitosis. Cells were synchronized into mitosis by thymidine release and nocodazole arrest. Mitotic cells were collected by shake-off and treated by 25 μM CQ for 1 hour. (D-E) The mitotic autophagy activity in ULK1-11A mutant or ATG13-4A mutant cells was determined by relative GFP/RFP ratio. Cells were treated as Fig 6C. (F) The effect of CDK1 inhibition by RO-3306 on autophagy. HeLa cells stably expressing GFP-LC3-RFP were treated by 10 μM RO-3306 for 4 hours in asynchronous condition and 20 minutes in mitotic condition synchronized by thymidine release and nocodazole arrest. Cells in (D, E, and F) were collected by flow cytometry for the relative GFP/RFP ratio. *n* = 3, *p < 0.05, **p < 0.01. Numerical data underlying the figure panels are available in S1 Data. 11A, S622&T635&T653&S479&S543&S413&T401&S403&S405&T282&T502A; ATG, autophagy-related; Baf A1, bafilomycin A1; CDK, cyclin-dependent kinase; CQ, chloroquine; DKO, double knockout; EBSS, Earle’s balanced salt solution; GFP, green fluorescent protein; mULK1, mouse ULK1; n.s., not significant; RFP, red fluorescent protein; ULK1, unc-51-like autophagy activating kinase 1.
(TIF)

S13 Fig ULK1-KO slightly delays S/G2 transition. (A) ULK1-KO does not affect cell cycle distribution in HeLa and 293T cells. Cell cycle distribution was analyzed by flow cytometry in asynchronous WT and ULK1-KO HeLa or 293T cells. (B) ULK1-KO slightly delays S/G2 transition. HeLa WT or ULK1-KO cells synchronized with double-thymidine and nocodazole were subjected to cell cycle analysis by flow cytometry. Numerical data underlying the figure panels are available in S1 Data. KO, knockout; ULK1, unc-51-like autophagy activating kinase 1; WT, wild type.
(TIF)

S14 Fig Mitotic entry was delayed in ULK1-KO, ATG7/ATG9A-KO but not K46I kinase-dead ULK1 cell lines. (A-B) Mitotic index is decreased in ULK1-KO cells synchronized by single-thymidine and nocodazole. HeLa WT or ULK1-KO cells synchronized into mitosis released from thymidine for 5 hours and nocodazole for another 7 hours were subjected to either PI and pH3(S10) co-staining for cell cycle and mitotic index analysis by flow cytometry (A) or western blot analysis for cell cycle markers (B). (C) Mitotic progression was not affected by K46I kinase-dead ULK1. HeLa ULK1-KO cells reconstituted with FLAG-tagged WT or K46I kinase-dead mULK1 were synchronized into mitosis and subjected to pH3(S10) staining for mitotic index analysis by flow cytometry. (D-E) The effect of ATG7/ATG9A-KO on mitotic entry. HeLa cells with ATG7 or ATG9A-KO were established by CRISPR/Cas9 (D) and treated as above for the detection of mitotic index analyzed by 1-way ANOVA followed by Tukey’s multiple comparison test (E). *n* = 3, *p < 0.05, ***p < 0.001, ****p < 0.0001. Numerical data underlying the figure panels are available in S1 Data. ATG, autophagy-related; KO, knockout; mULK1, mouse ULK1; n.s., not significant; PI, propidium iodide; ULK1, unc-51-like autophagy activating kinase 1; WT, wild type.
(TIF)

S15 Fig ULK1 and ATG13 coordinate to regulate cell cycle progression. (A-B) Mitotic index was decreased in ATG13-KO cells synchronized by single-thymidine and nocodazole. HeLa WT or ATG13-KO cells synchronized into mitosis were subjected to PI and pH3(S10) co-staining for cell cycle and mitotic index analysis by flow cytometry (A) or western blot analysis for cell cycle markers (B). (C) ULK1-KO combined with ATG13 “knockdown” inhibits S/G2 transition. HeLa ULK1-KO cells transiently transfected with the CRISPR/Cas9 vector control or plasmid subcloned gRNA for human ATG13 were synchronized with thymidine and nocodazole for cell cycle analysis by flow cytometry. (D) The cell lysate collected from (C) was subjected to western blot analysis by indicated antibodies. (E) ULK1 and ATG13 DKO decreases mitotic index. HeLa WT or ULK1&ATG13-DKO cells were subjected to pH3(S10) staining for mitotic index analysis by flow cytometry. *n* = 3, *p < 0.05, **p < 0.01. Numerical data underlying the figure panels are available in S1 Data. ATG, autophagy-related; DKO, double knockout; gRNA, guide RNA; KO, knockout; n.s., not significant; PI, propidium iodide; ULK1, unc-51-like autophagy activating kinase 1; WT, wild type.
(TIF)

S16 Fig Rescue efficiency determined by relative mitotic index and doubling time. (A) Cells synchronized by thymidine and nocodazole were subjected to PI and pH3(S10) co-staining for cell cycle and mitotic index analysis by flow cytometry. (B) Doubling time of various cell lines. The statistical analysis (1-way ANOVA followed by Tukey’s multiple comparison test) was done by comparing indicated cells to HeLa cells. The doubling time is calculated as Fig 7D. *n* = 3, *p < 0.05, **p < 0.01, ****p < 0.0001. Numerical data underlying the figure panels are available in S1 Data. n.s., not significant; PI, propidium iodide.
(TIF)

S17 Fig The role of ULK1-11A/ATG13-4A unphosphorylatable mutant in cell cycle progression. (A, D) The cell cycle distribution and mitotic entry of HeLa ULK1-KO cells stably overexpressing WT or mutant 11A FLAG-tagged mULK1 and HeLa ATG13-KO cells stably overexpressing WT or mutant 4A FLAG-tagged ATG13. Cells released from thymidine for 11 hours were subjected to PI and pH3(S10) co-staining for cell cycle and mitotic index analysis by flow cytometry. (B, E) Mitotic exit of ULK1 or ATG13 WT or mutant cells. Cells were synchronized into mitosis with thymidine and nocodazole and released into nocodazole-free complete DMEM medium for different time points and then subjected to either PI or cyclin B1 staining for cell cycle, and cyclin B1 level analysis by flow cytometry. (C, F) ULK1 or ATG13 WT or mutant does not affect cell proliferation. Cells were plated at 1 × 10^5^ cells/mL and cultured for 1, 2, or 3 days. The cell number was counted by flow cytometry, and the doubling time was calculated. *T*_D_ indicates the average cell doubling time and is calculated as: *T*_D_ = t_*_[lg2/(lgNt − lgN0)], where t is the culture time, Nt is the cell number after culturing, and N0 is the original cell number plated. *n* = 3, *p < 0.05, **p < 0.01. Numerical data underlying the figure panels are available in S1 Data. 11A, S622&T635&T653&S479&S543&S413&T401&S403&S405&T282&T502A; ATG, autophagy-related; DMEM, Dulbecco’s Modified Eagle Medium; KO, knockout; mULK1, mouse ULK1; n.s., not significant; PI, propidium iodide; ULK1, unc-51-like autophagy activating kinase 1; WT, wild type.
(TIF)

S18 Fig Mouse model. (A) The mouse model in nude mice bearing WT or KO cells treated with or without ULK1 kinase inhibitor SBI-0206965 was established. (B) Time course of the body weight for nude mice in (A). Numerical data underlying the figure panels are available in S1 Data. KO, knockout; ULK1, unc-51-like autophagy activating kinase 1; WT, wild type.
(TIF)

## Abbreviations

3A: S622/T635/T653A
4A: T342/T332/S44/S224A
5A: 3A-S479&S543A
7A: 5A-S411&S413A
9A: 5A-S413&T401&S403&S405A
10A: 9A-T282A
11A: S622&T635&T653&S479&S543&S413&T401&S403&S405&T282&T502A
AMPK: AMP-activated protein kinase
ATG: autophagy-related
CDK: cyclin-dependent kinase
CQ: chloroquine
DKO: double knockout
EBSS: Earle’s balanced salt solution
FIP200: FAK family-interacting protein of 200 kDa
GABARAP: gamma-aminobutyric acid receptor-associated protein
GAPDH: glyceraldehyde 3-phosphate dehydrogenase
GFP: green fluorescent protein
gRNA: guide RNA
HCT 116: human colorectal cancer cell
HEK-293T: human embryonic kidney 293T
IP: immunoprecipitation
KO: knockout
MAPK: mitogen-activated protein kinase
MSCV: murine stem cell virus
mTOR: mammalian target-of-rapamycin
mTORC1: mammalian-target-of rapamycin complex 1
mULK1: mouse ULK1
Myt1: Myelin transcription factor 1
NOC: nocodazole
n.s.: not significant
PAS: pre-autophagosomal structure
pH3(S10): phospho histone H3 serine 10
PI: propidium iodide
PKCδ: protein kinase C-delta
PPI: phosphatase inhibitor
Pro_ST_kin: proline-dependent serine/threonine kinase group
PtdIns3P: phosphatidylinositol-3-phosphate
PVDF: polyvinylidene fluoride
RFP: red fluorescent protein
RPE1: human retinal pigmented epithelial cells
SGK: serum/glucocorticoid-induced kinase
SQSTM1: sequestosome 1
ULK1: unc-51 like autophagy activating kinase 1
WT: wild type
λPP: lambda phosphatase
