# Supplementary material for: ULK1-ATG13 and their mitotic phospho-regulation by CDK1 connect autophagy to cell cycle
Source: PLoS Biol. 2020 Jun 9;18(6):e3000288. doi: 10.1371/journal.pbio.3000288 (PMC7282624; doi:10.1371/journal.pbio.3000288)
Supplement: S1 Raw Images — (PDF) [file pbio.3000288.s002.pdf]

Main Figures

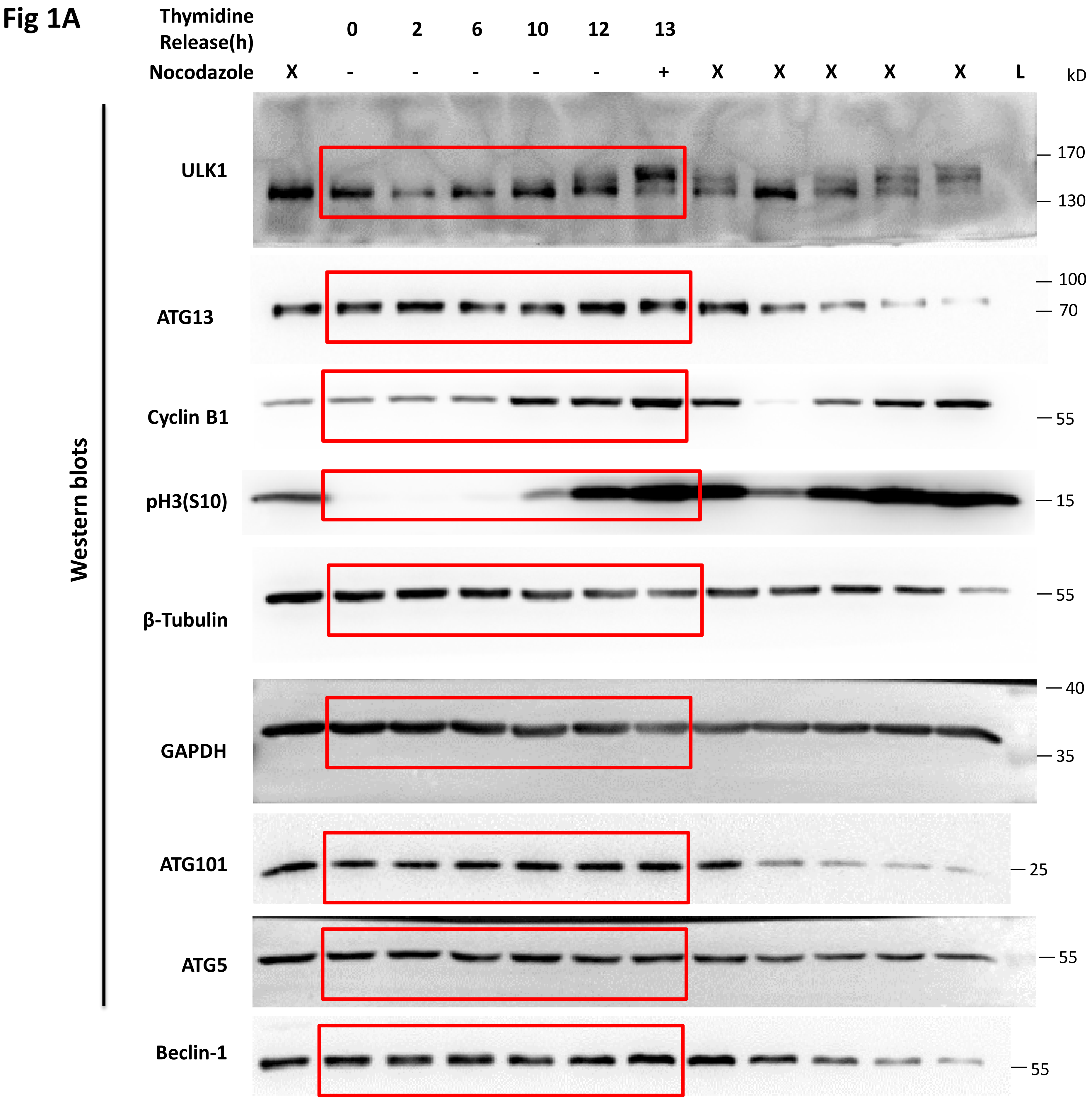

Images were acquired by chemiluminescence CCD camera-based digital imaging instruments (Tanon Fine-do X6).
Approx. molecular weight ladder (L) was indicated.
PVDF Membranes were cut for immunoblotting of more than one protein.

Fig 1B

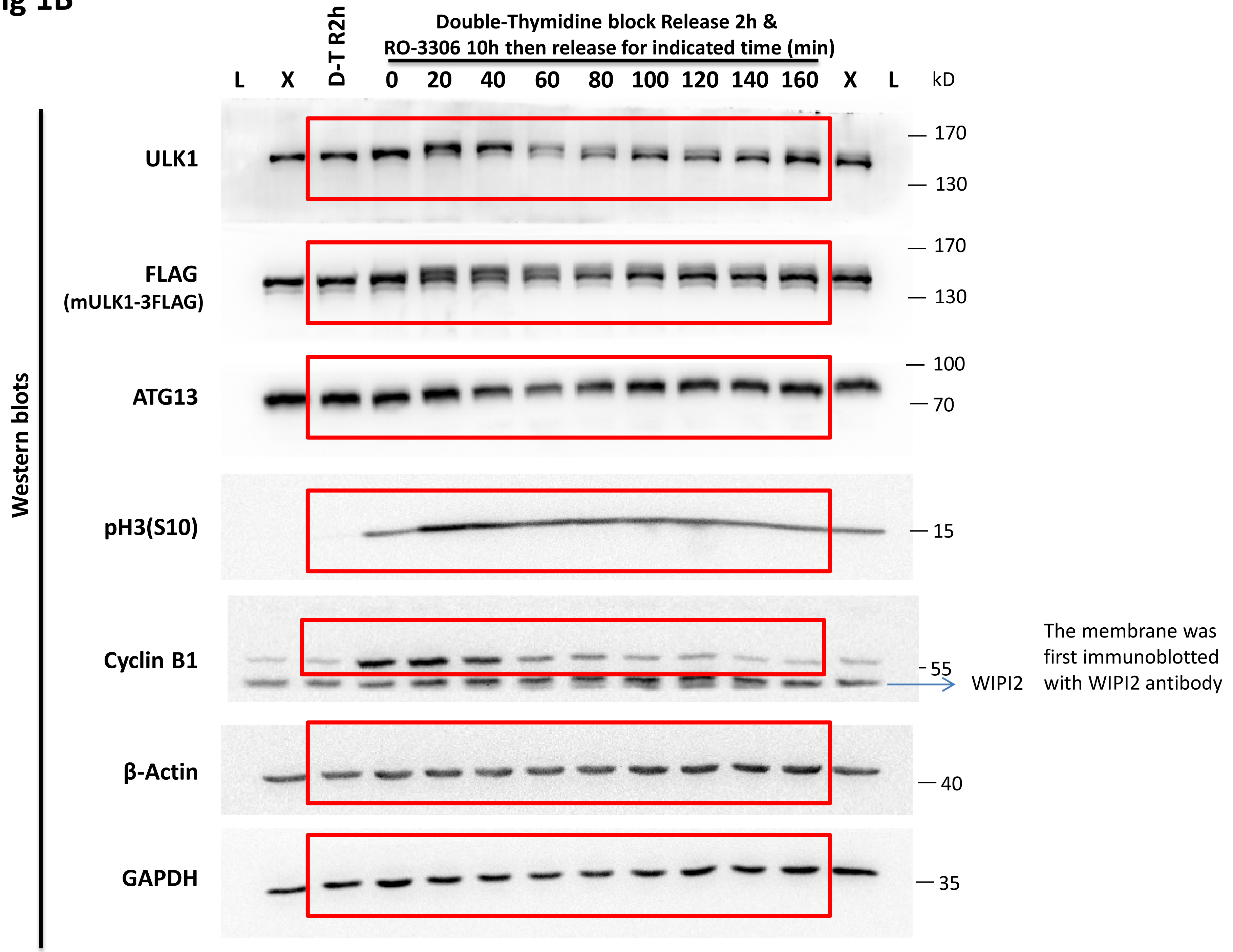

Images were acquired by chemiluminescence CCD camera-based digital imaging instruments (Tanon Fine-do X6).
Approx. molecular weight ladder (L) was indicated.
PVDF Membranes were cut for immunoblotting of more than one protein.

Fig 1C

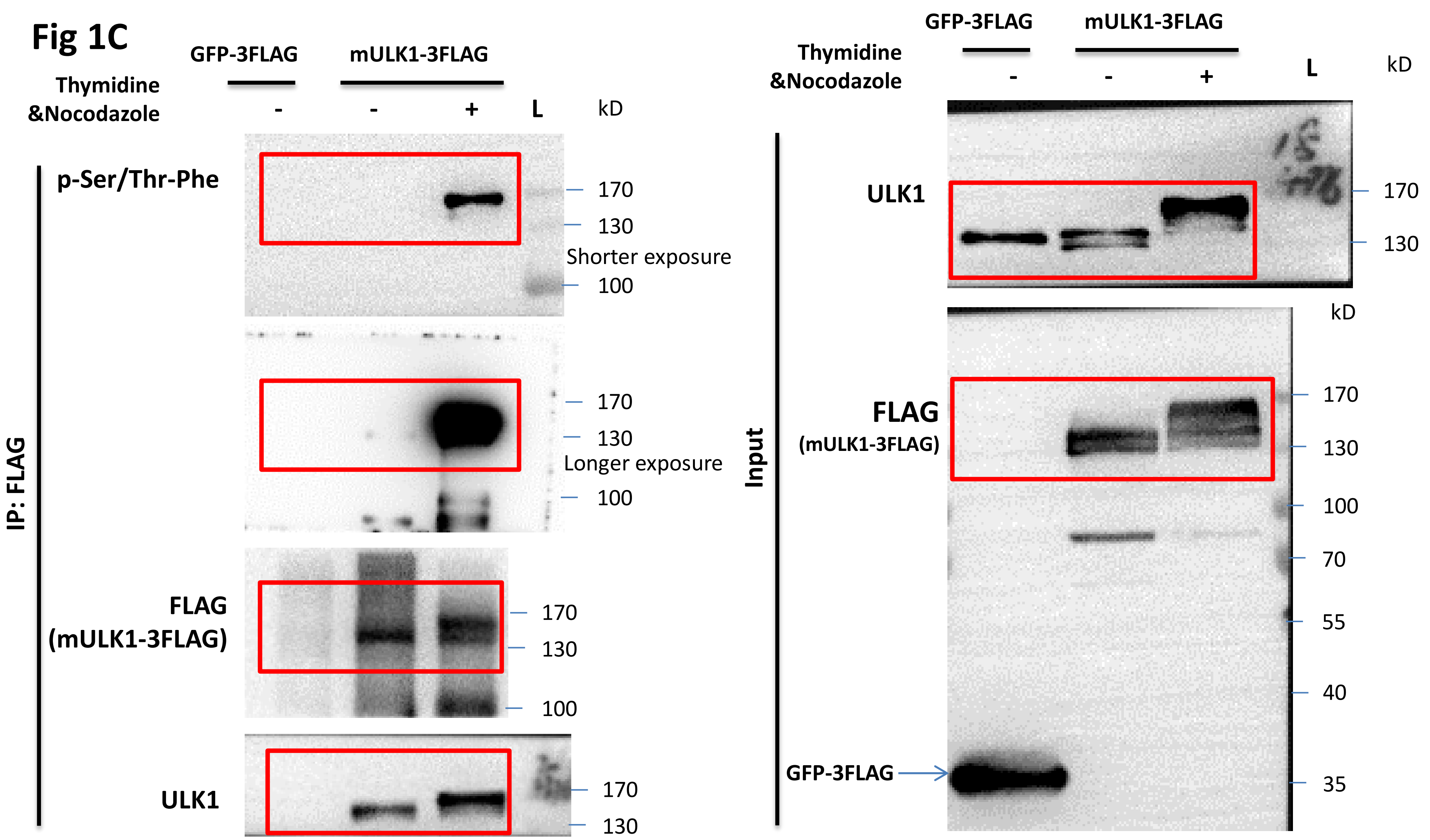

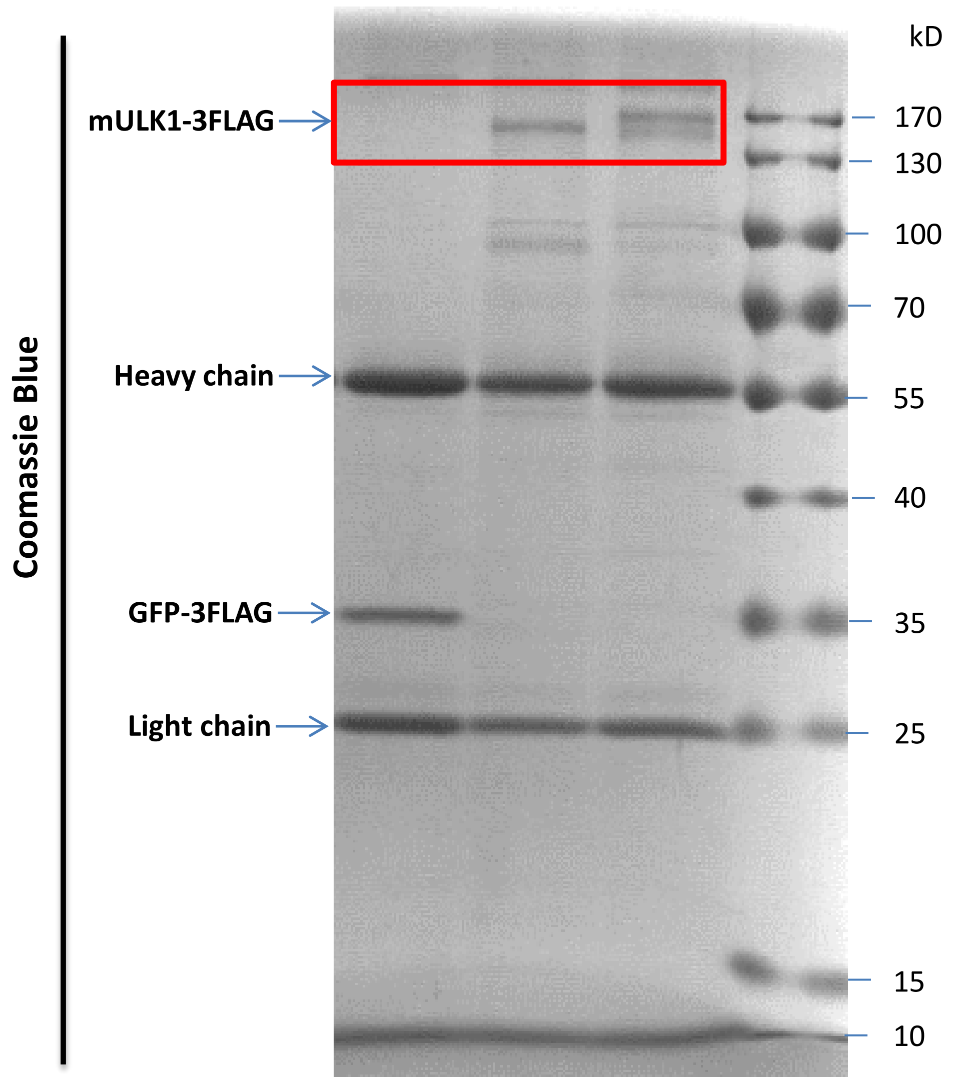

Images were acquired by chemiluminescence CCD camera-based digital imaging instruments (Tanon Fine-do X6). Approx. molecular weight ladder (L) was indicated. PVDF Membranes were cut for immunoblotting of more than one protein.

Fig 1D

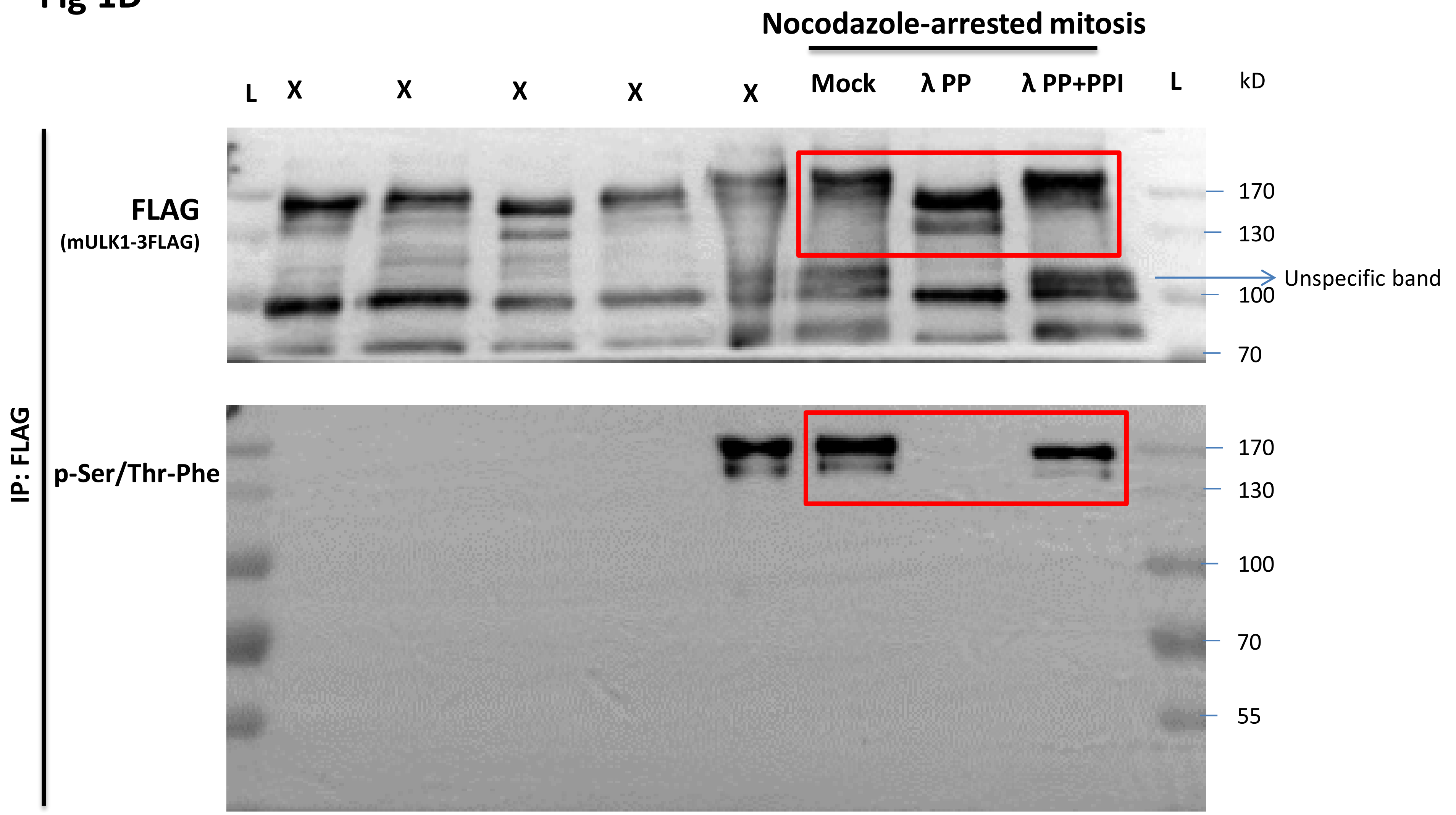

Images were acquired by chemiluminescence CCD camera-based digital imaging instruments (Tanon Fine-do X6).
Approx. molecular weight ladder (L) was indicated.
PVDF Membranes were cut for immunoblotting of more than one protein.

Fig 1E

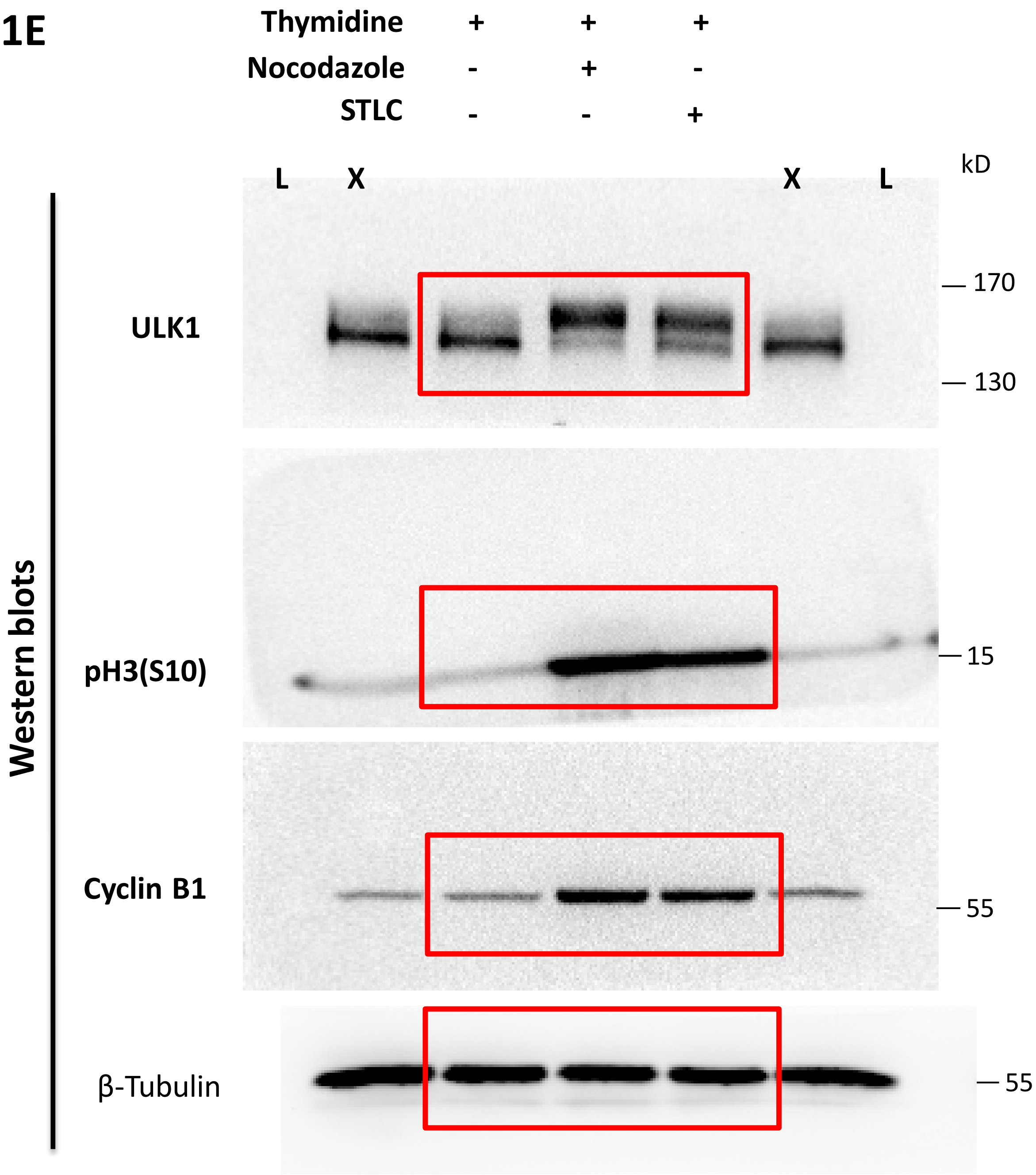

Images were acquired by chemiluminescence CCD camera-based digital imaging instruments (Tanon Fine-do X6).
Approx. molecular weight ladder (L) was indicated.
PVDF Membranes were cut for immunoblotting of more than one protein.

Fig 1F

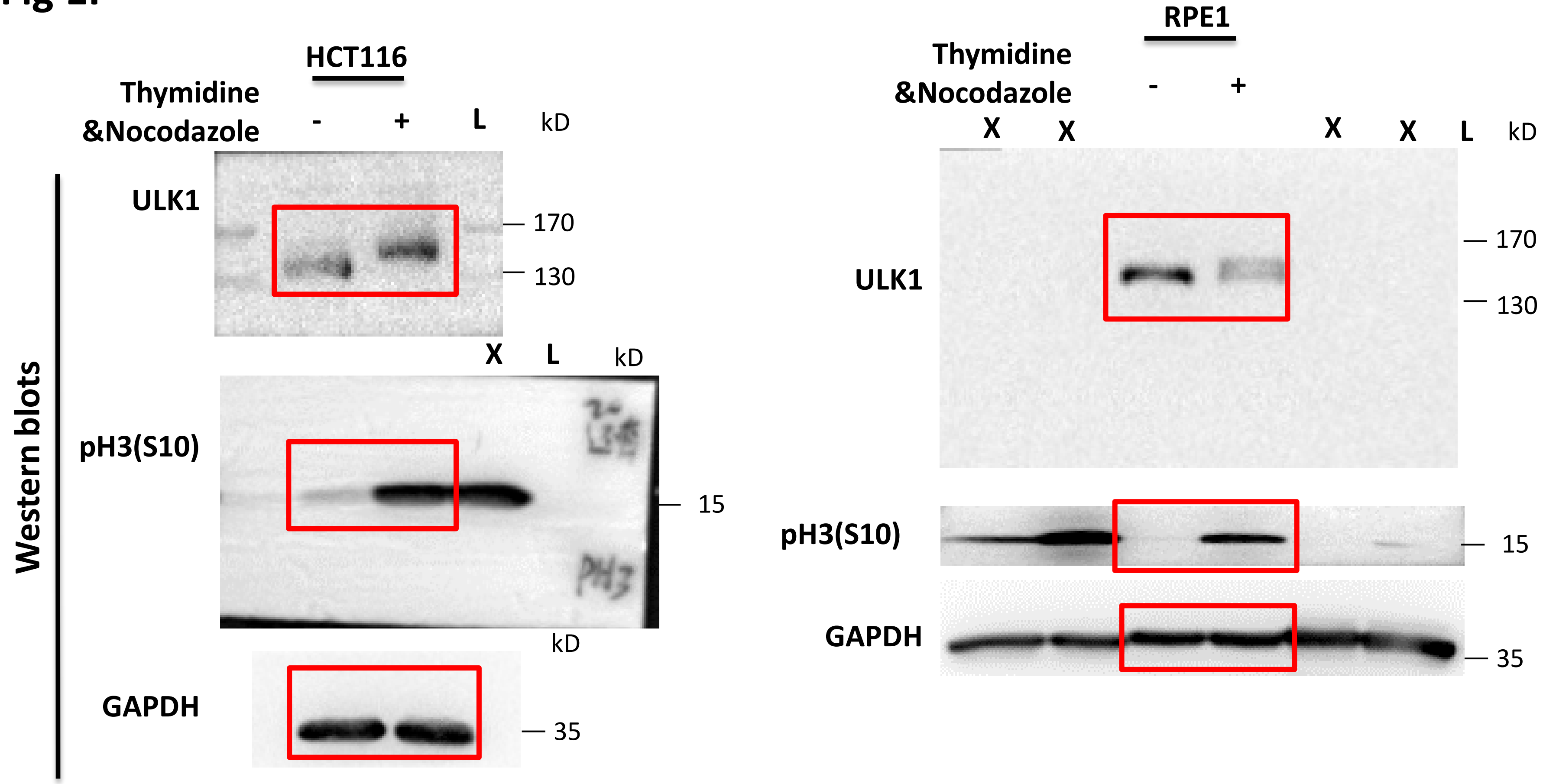

Images were acquired by chemiluminescence CCD camera-based digital imaging instruments (Tanon Fine-do X6).
Approx. molecular weight ladder (L) was indicated.
PVDF Membranes were cut for immunoblotting of more than one protein.

Fig 2A

Upper panel

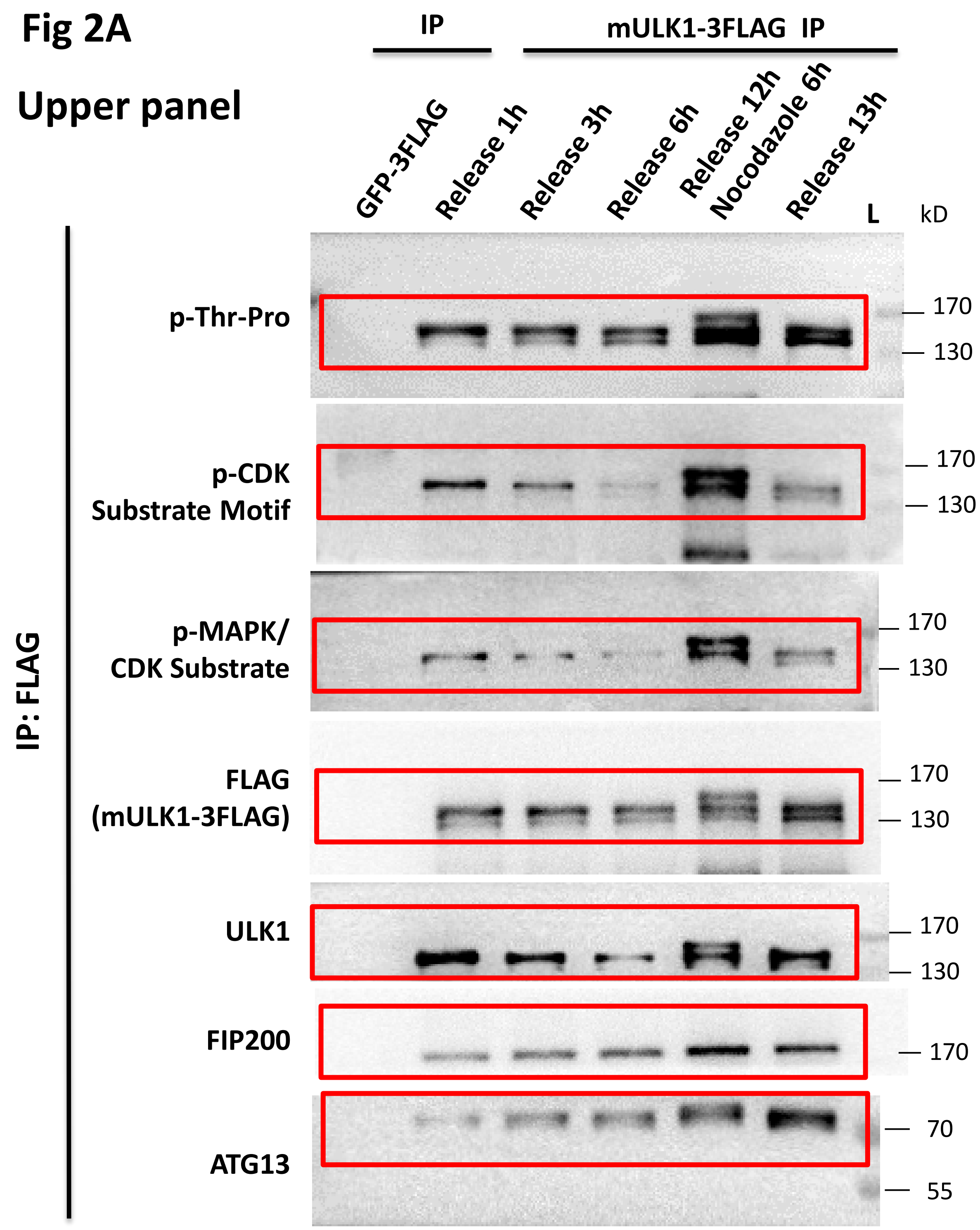

Images were acquired by chemiluminescence CCD camera-based digital imaging instruments (Tanon Fine-do X6).
Approx. molecular weight ladder (L) was indicated.
PVDF Membranes were cut for immunoblotting of more than one protein.

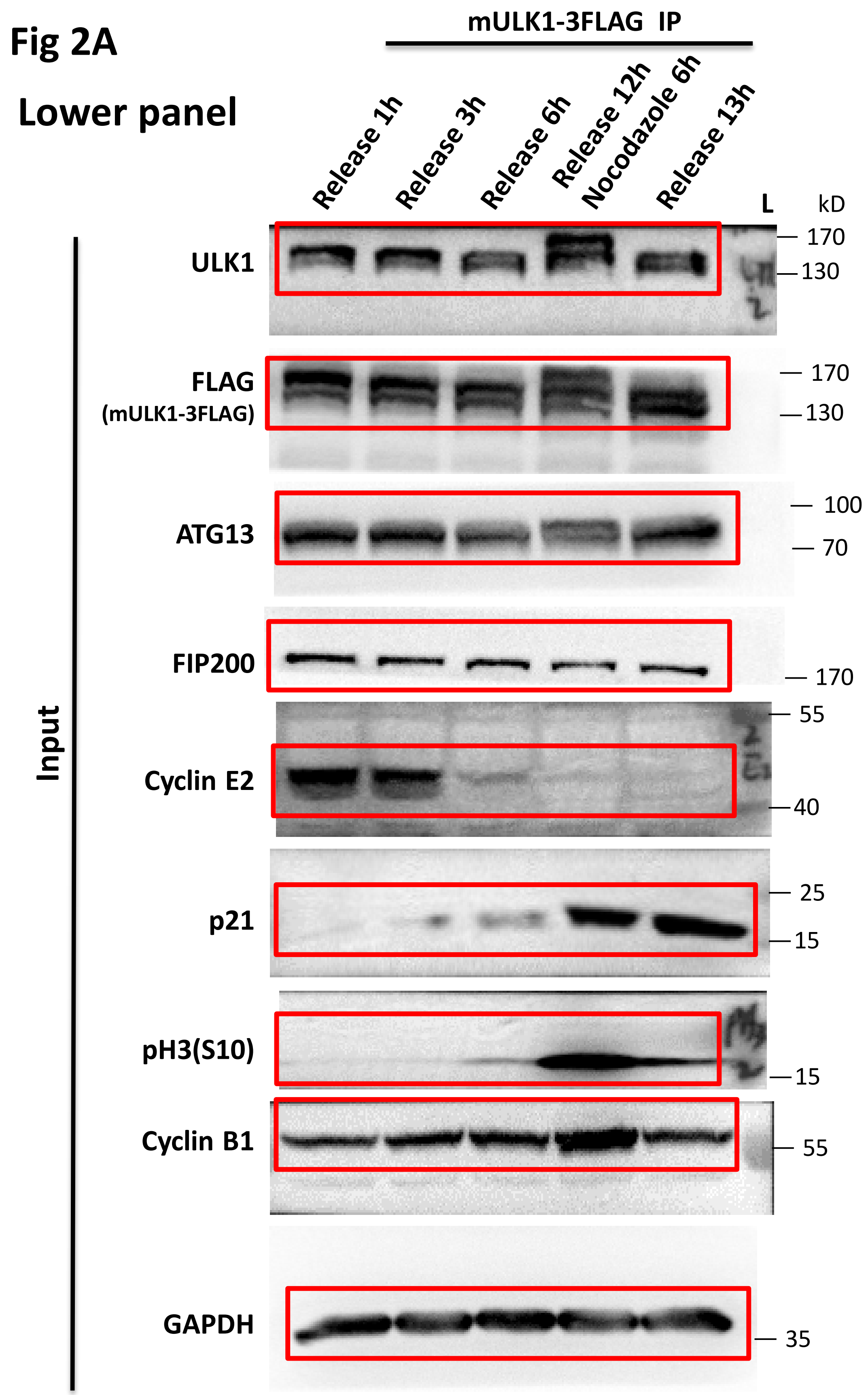

Images were acquired by chemiluminescence CCD camera-based digital imaging instruments (Tanon Fine-do X6).
Approx. molecular weight ladder (L) was indicated.
PVDF Membranes were cut for immunoblotting of more than one protein.

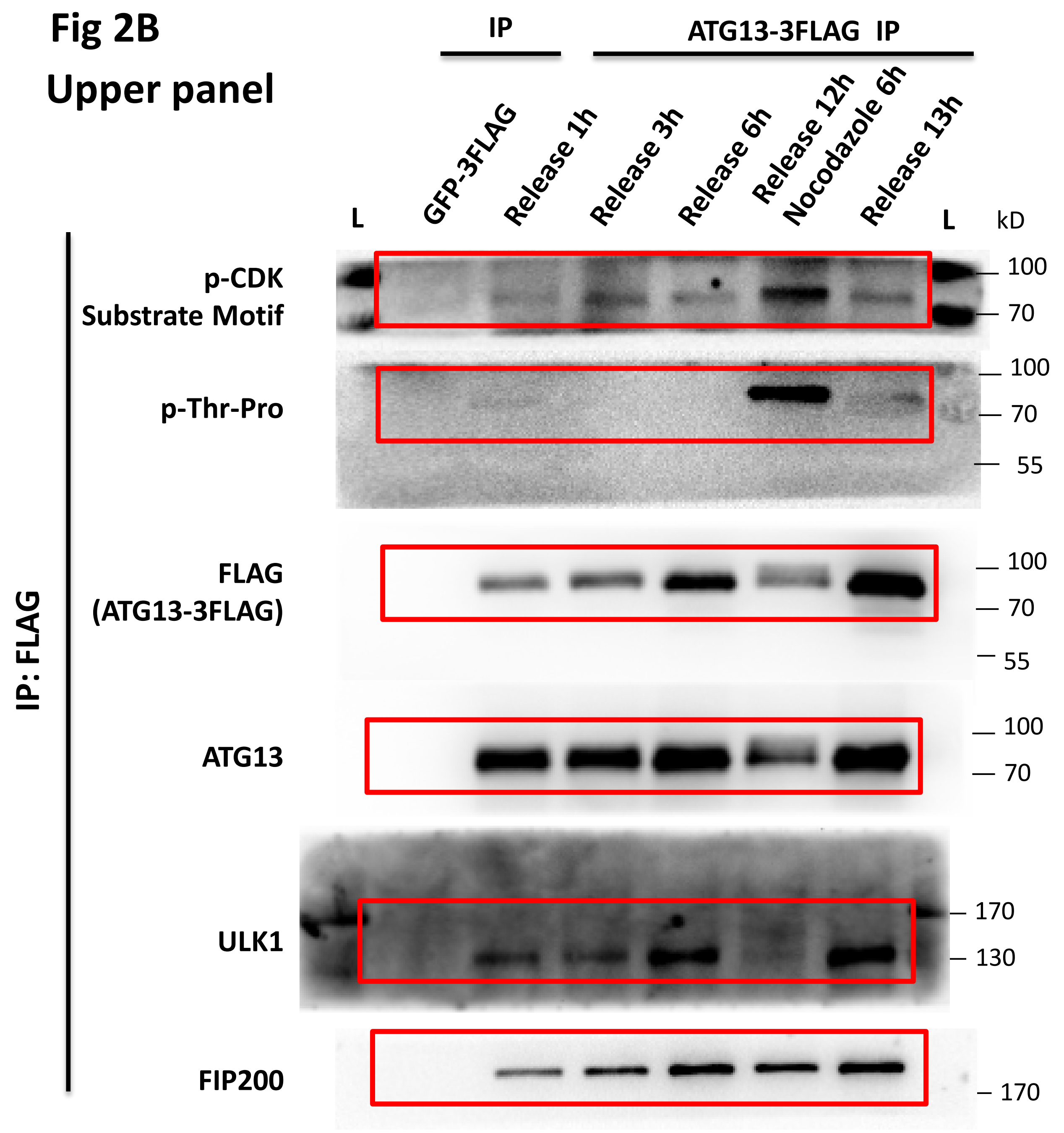

Images were acquired by chemiluminescence CCD camera-based digital imaging instruments (Tanon Fine-do X6).
Approx. molecular weight ladder (L) was indicated.
PVDF Membranes were cut for immunoblotting of more than one protein.

Fig 2B

Lower panel

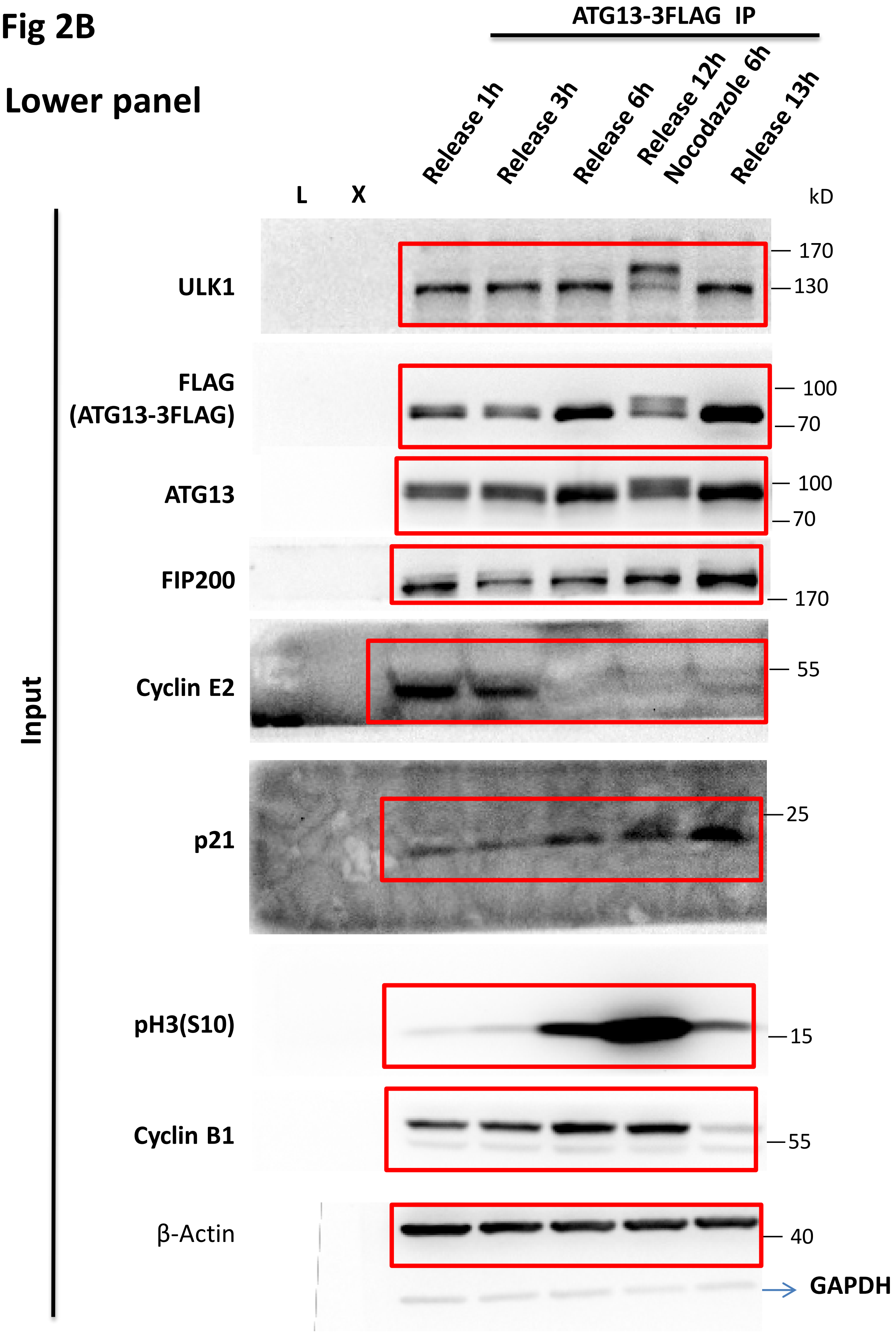

Images were acquired by chemiluminescence CCD camera-based digital imaging instruments (Tanon Fine-do X6).
Approx. molecular weight ladder (L) was indicated.
PVDF Membranes were cut for immunoblotting of more than one protein.

Fig 3B

Upper panel

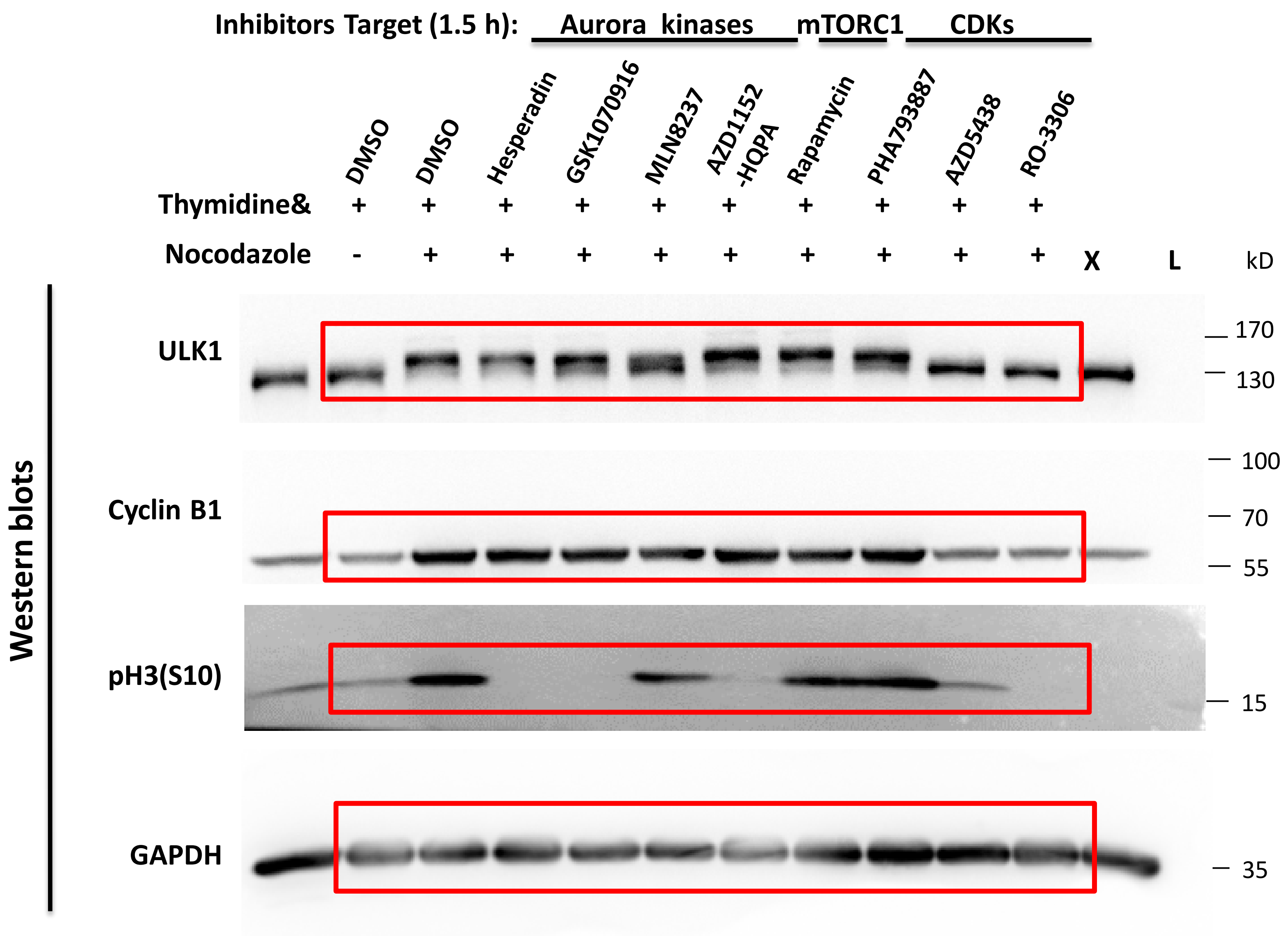

Images were acquired by chemiluminescence CCD camera-based digital imaging instruments (Tanon Fine-do X6).
Approx. molecular weight ladder (L) was indicated.
PVDF Membranes were cut for immunoblotting of more than one protein.

Fig 3B

Lower panel

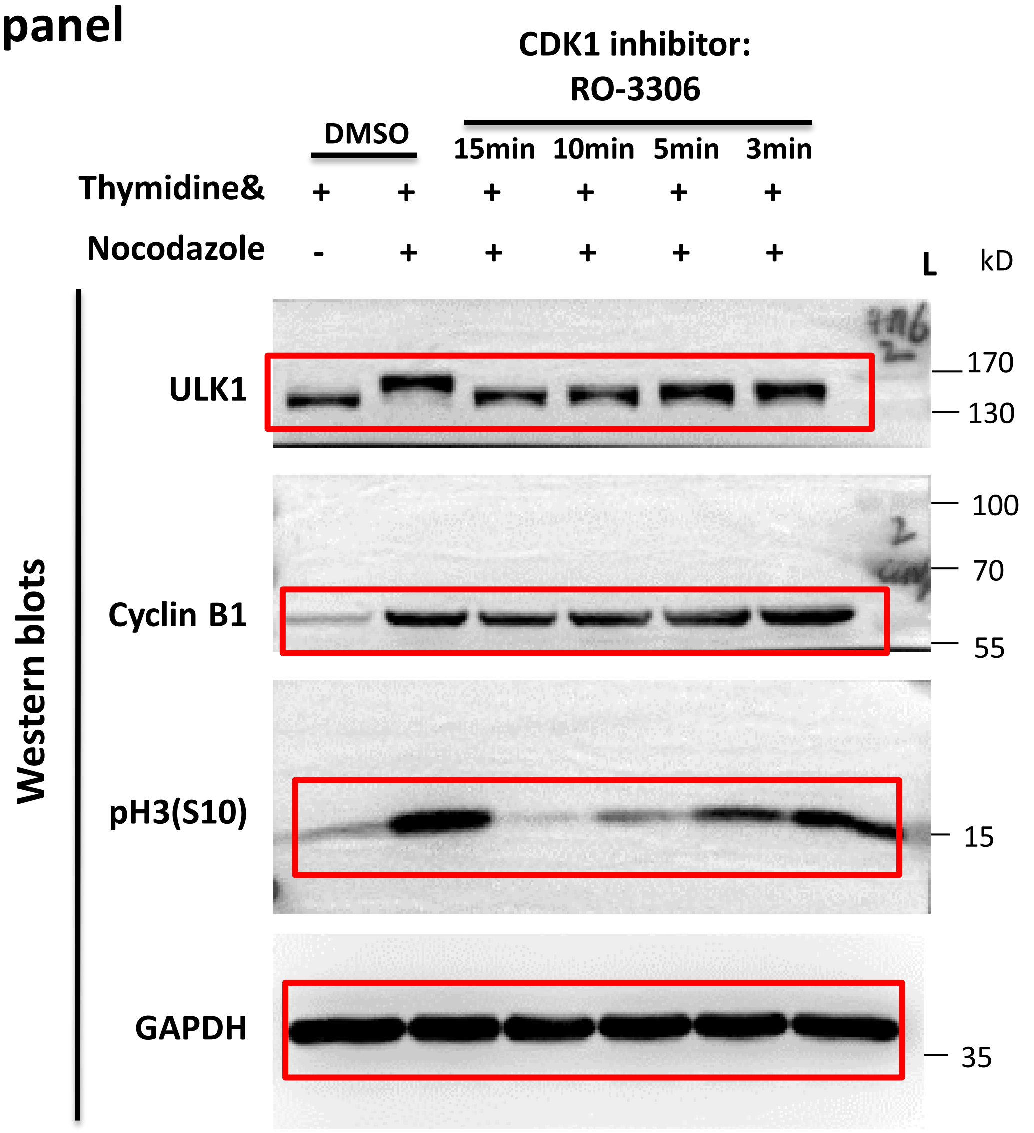

Images were acquired by chemiluminescence CCD camera-based digital imaging instruments (Tanon Fine-do X6).
Approx. molecular weight ladder (L) was indicated.
PVDF Membranes were cut for immunoblotting of more than one protein.

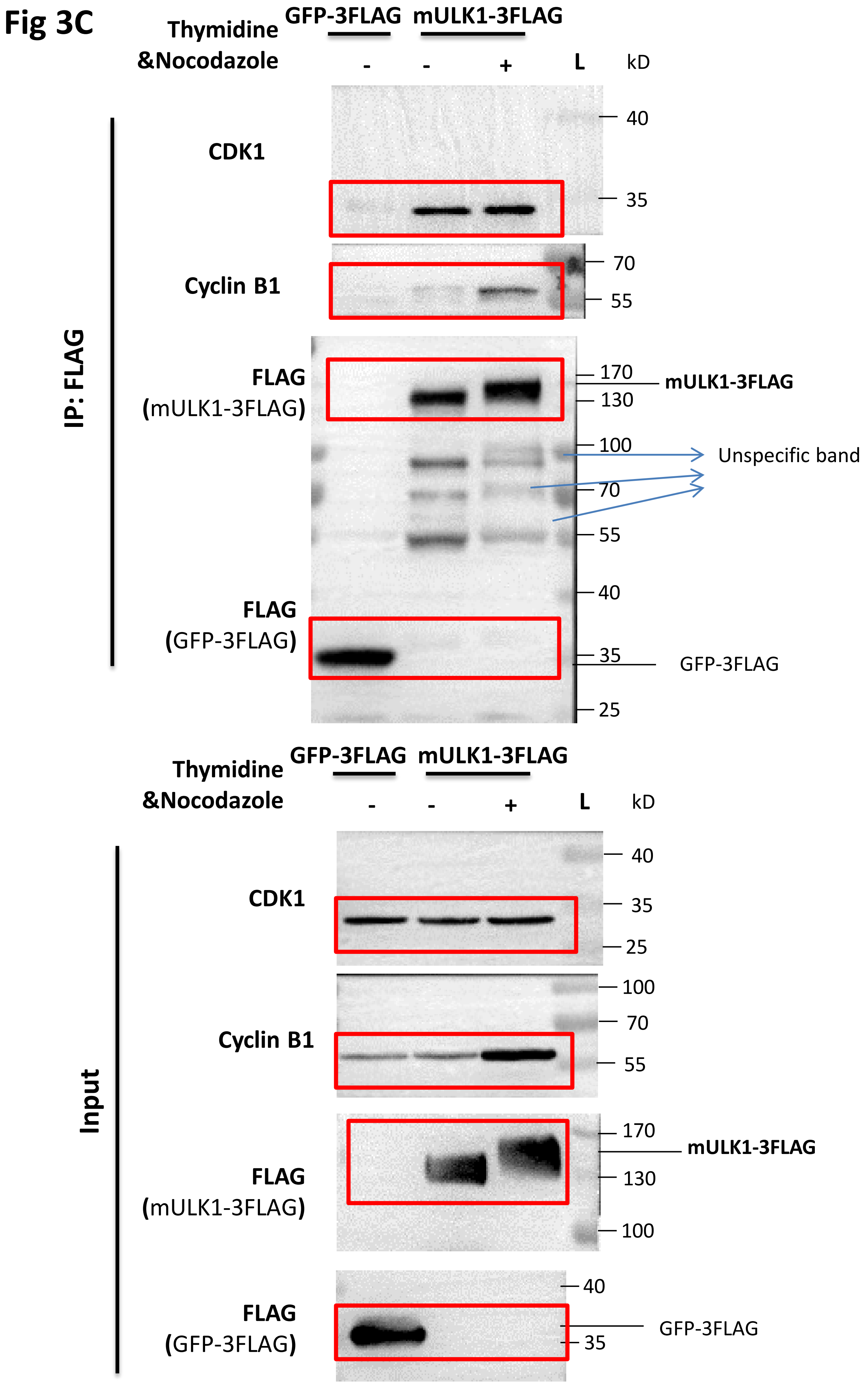

Images were acquired by chemiluminescence CCD camera-based digital imaging instruments (Tanon Fine-do X6).
Approx. molecular weight ladder (L) was indicated.
PVDF Membranes were cut for immunoblotting of more than one protein.

Fig 3D

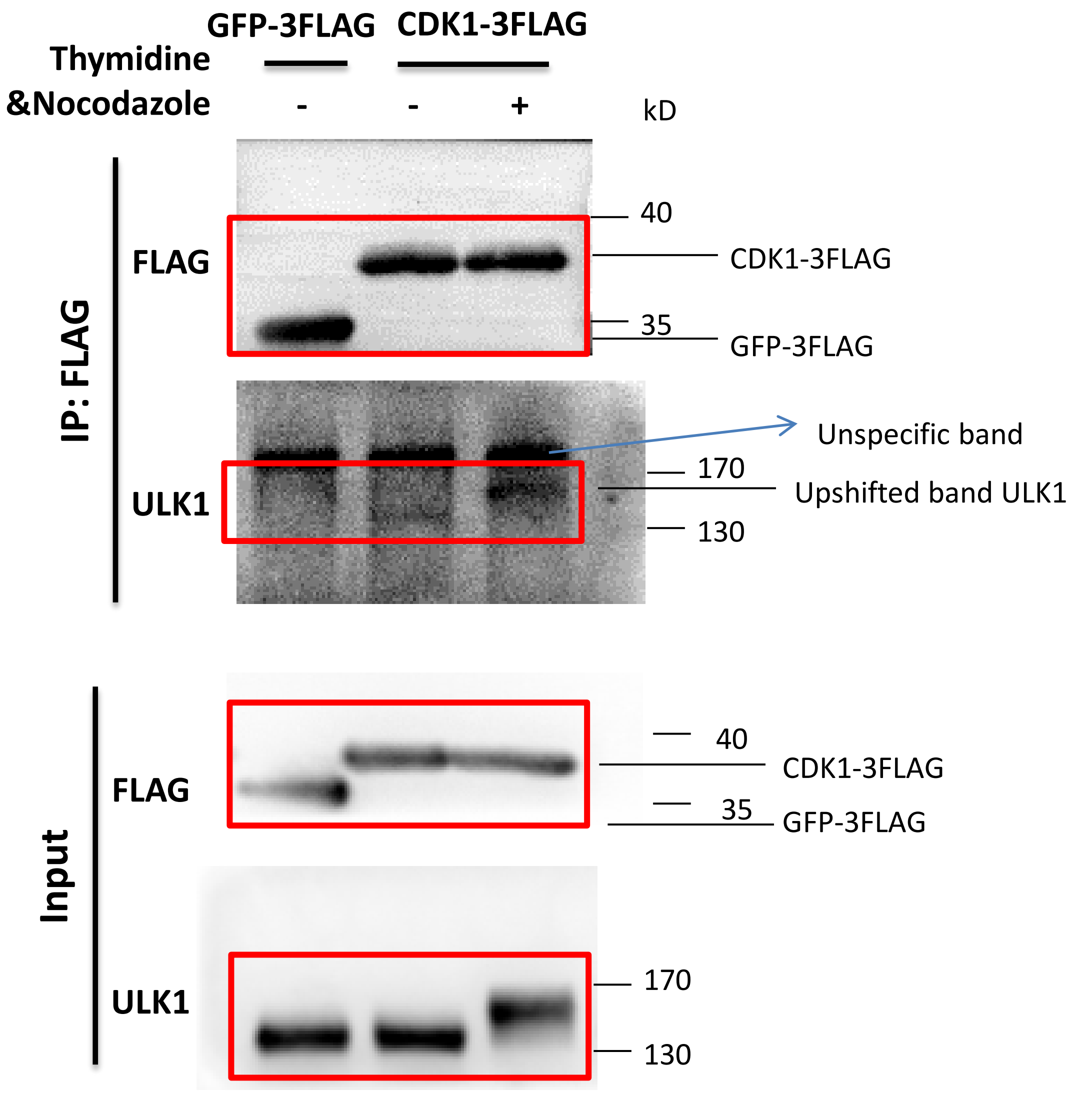

Images were acquired by chemiluminescence CCD camera-based digital imaging instruments (Tanon Fine-do X6).
Approx. molecular weight ladder (L) was indicated.
PVDF Membranes were cut for immunoblotting of more than one protein.

Fig 3E

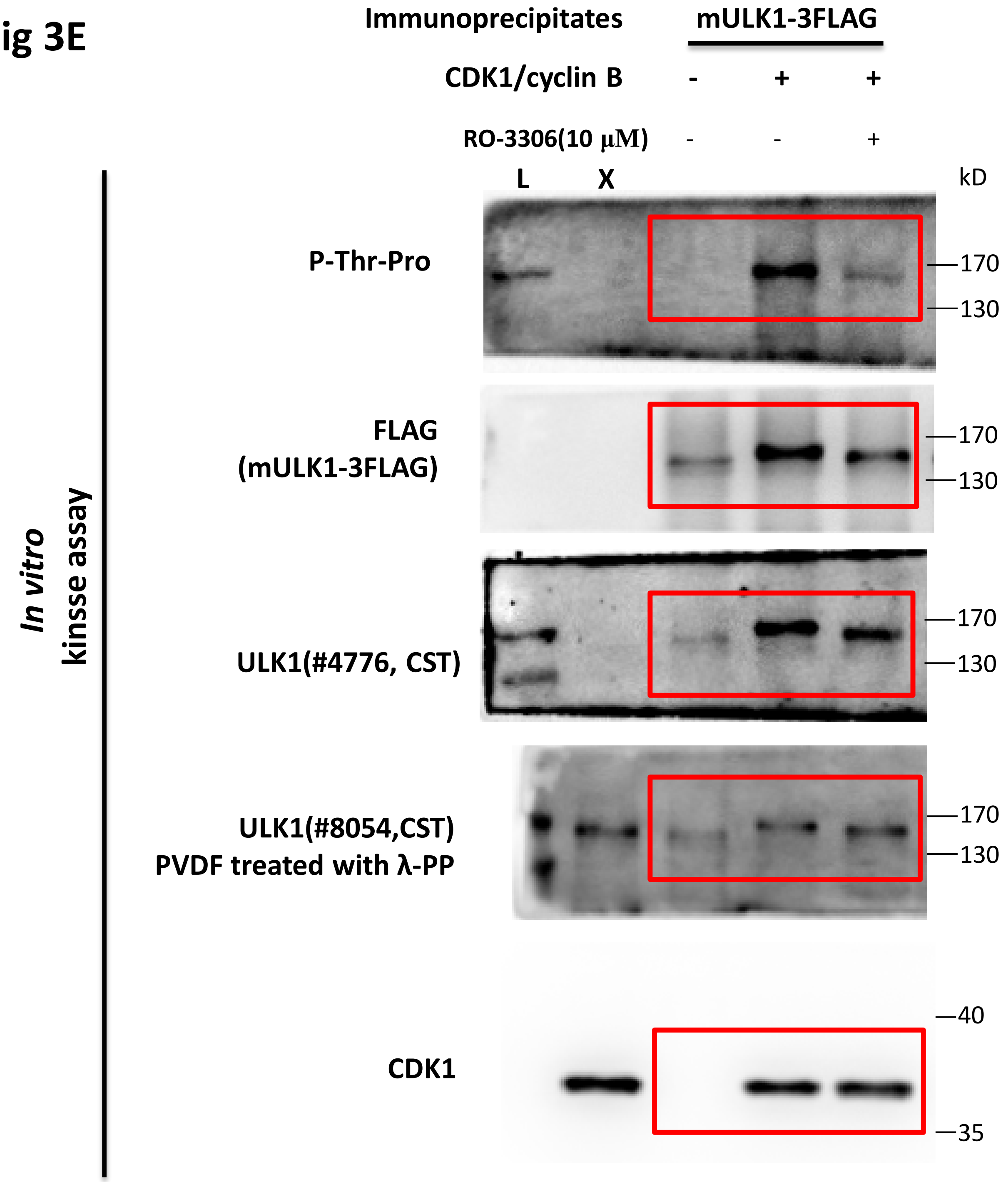

Images were acquired by chemiluminescence CCD camera-based digital imaging instruments (Tanon Fine-do X6).
Approx. molecular weight ladder (L) was indicated.
PVDF Membranes were cut for immunoblotting of more than one protein.

Fig 4A

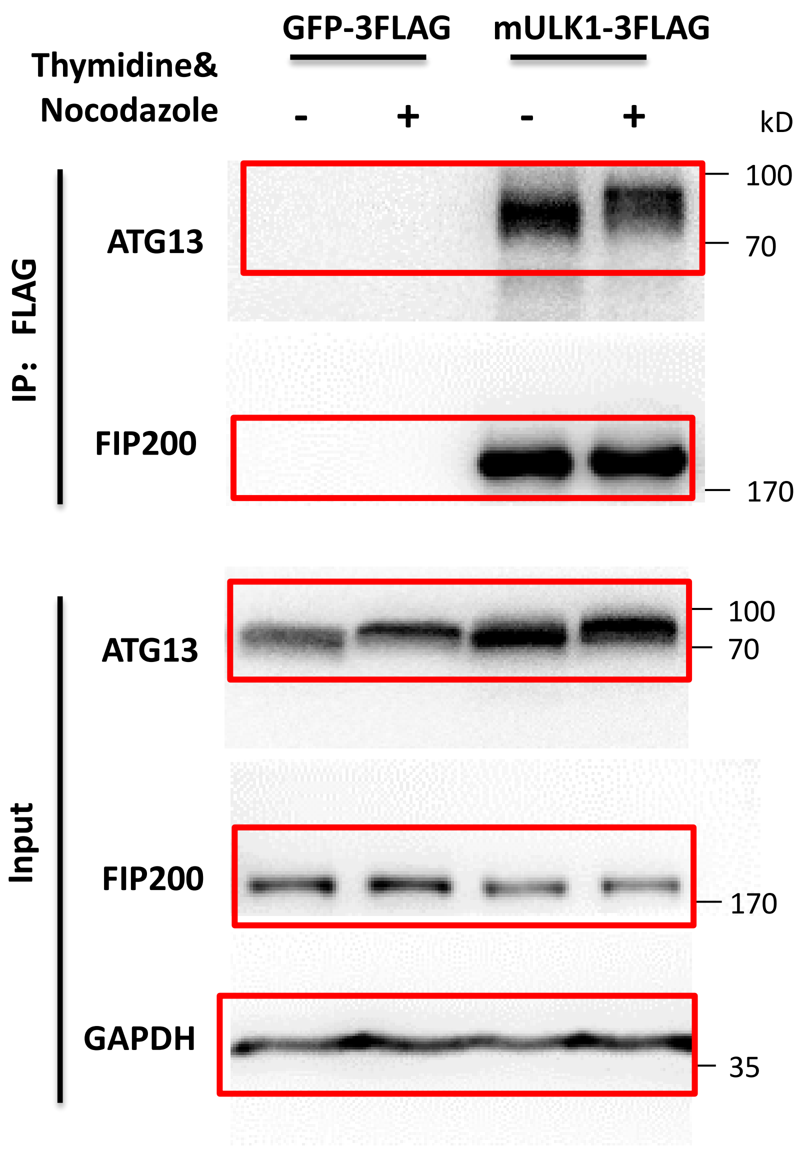

Images were acquired by chemiluminescence CCD camera-based digital imaging instruments (Tanon Fine-do X6).
Approx. molecular weight ladder (L) was indicated.
PVDF Membranes were cut for immunoblotting of more than one protein.

Fig 4B

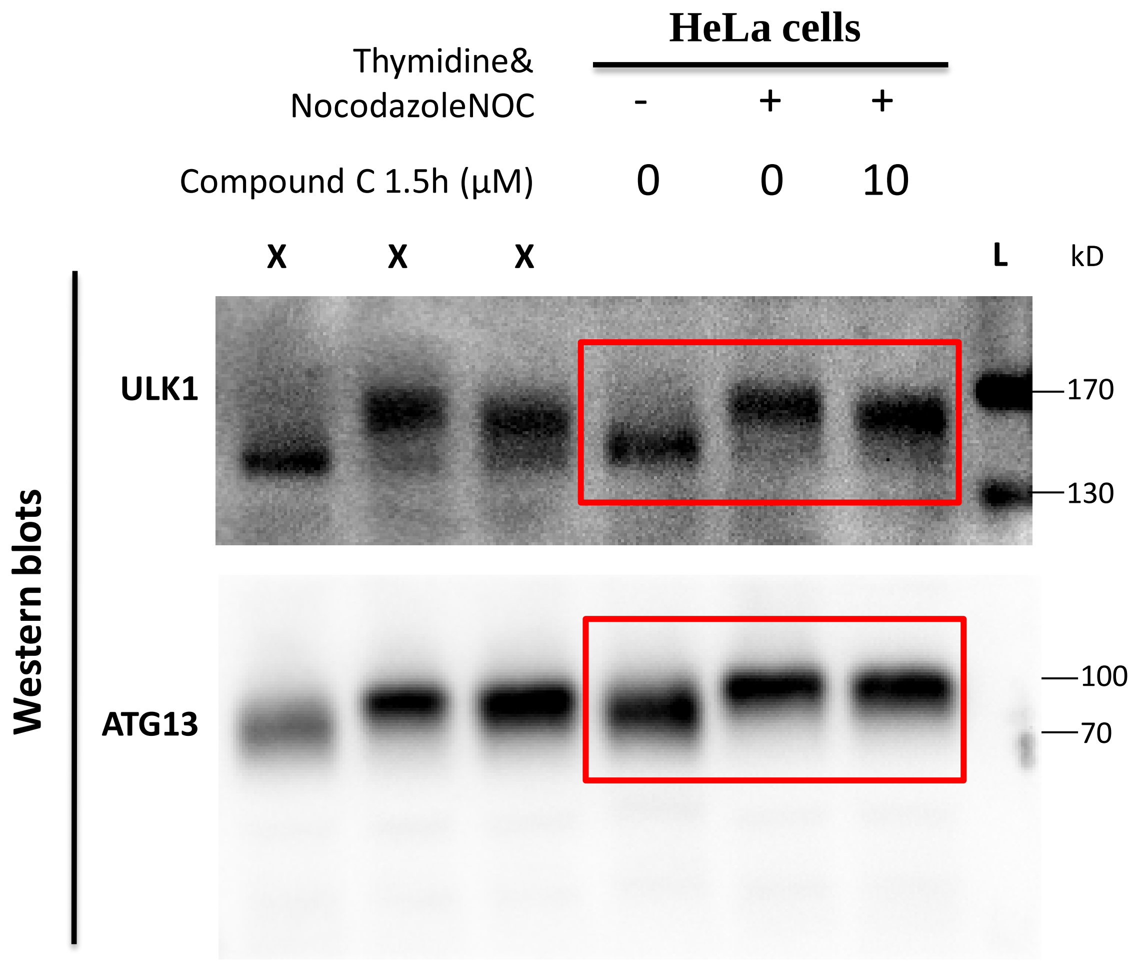

Images were acquired by chemiluminescence CCD camera-based digital imaging instruments (Tanon Fine-do X6).
Approx. molecular weight ladder (L) was indicated.
PVDF Membranes were cut for immunoblotting of more than one protein.

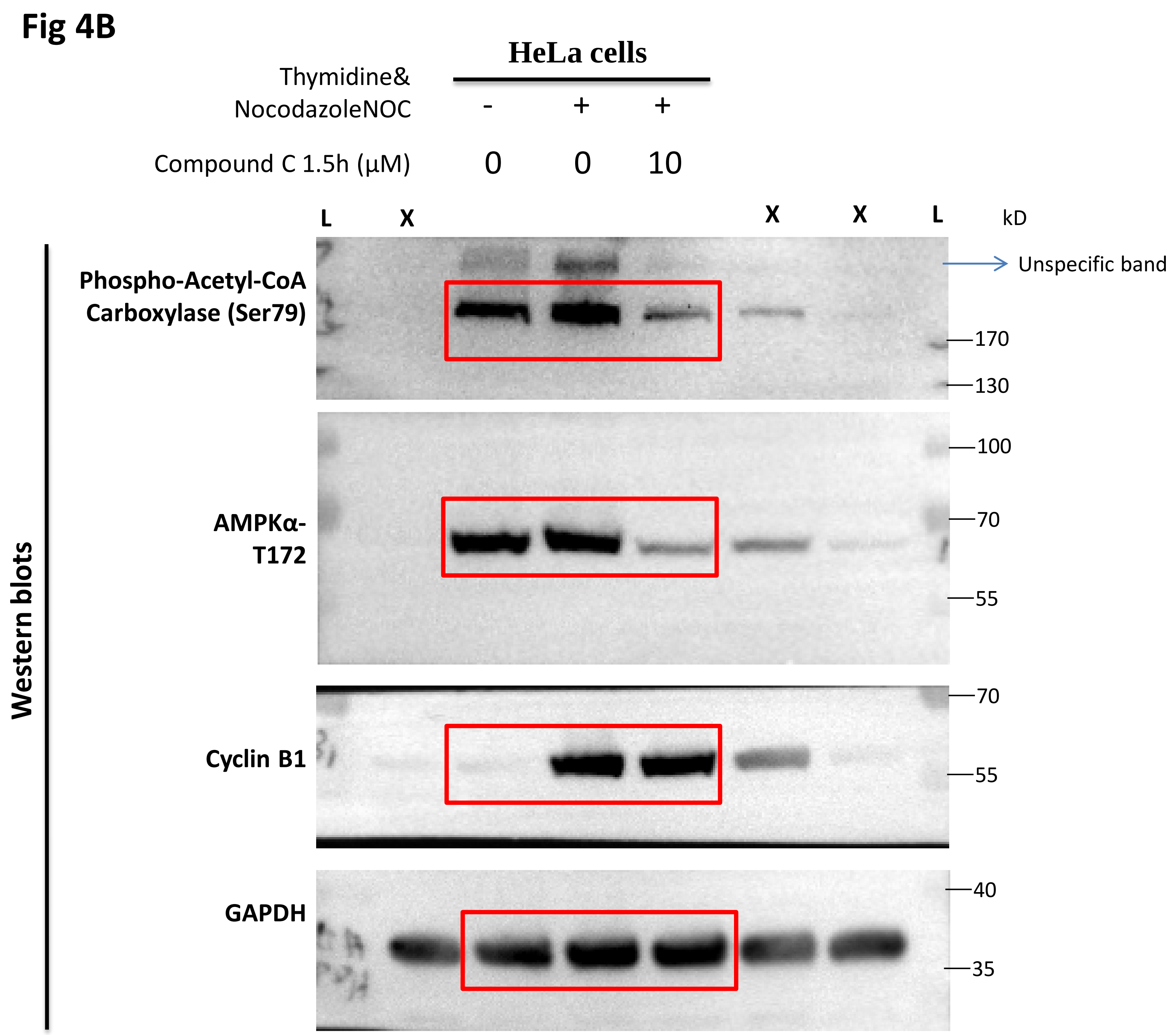

Images were acquired by chemiluminescence CCD camera-based digital imaging instruments (Tanon Fine-do X6).
Approx. molecular weight ladder (L) was indicated.
PVDF Membranes were cut for immunoblotting of more than one protein.

Fig 4C

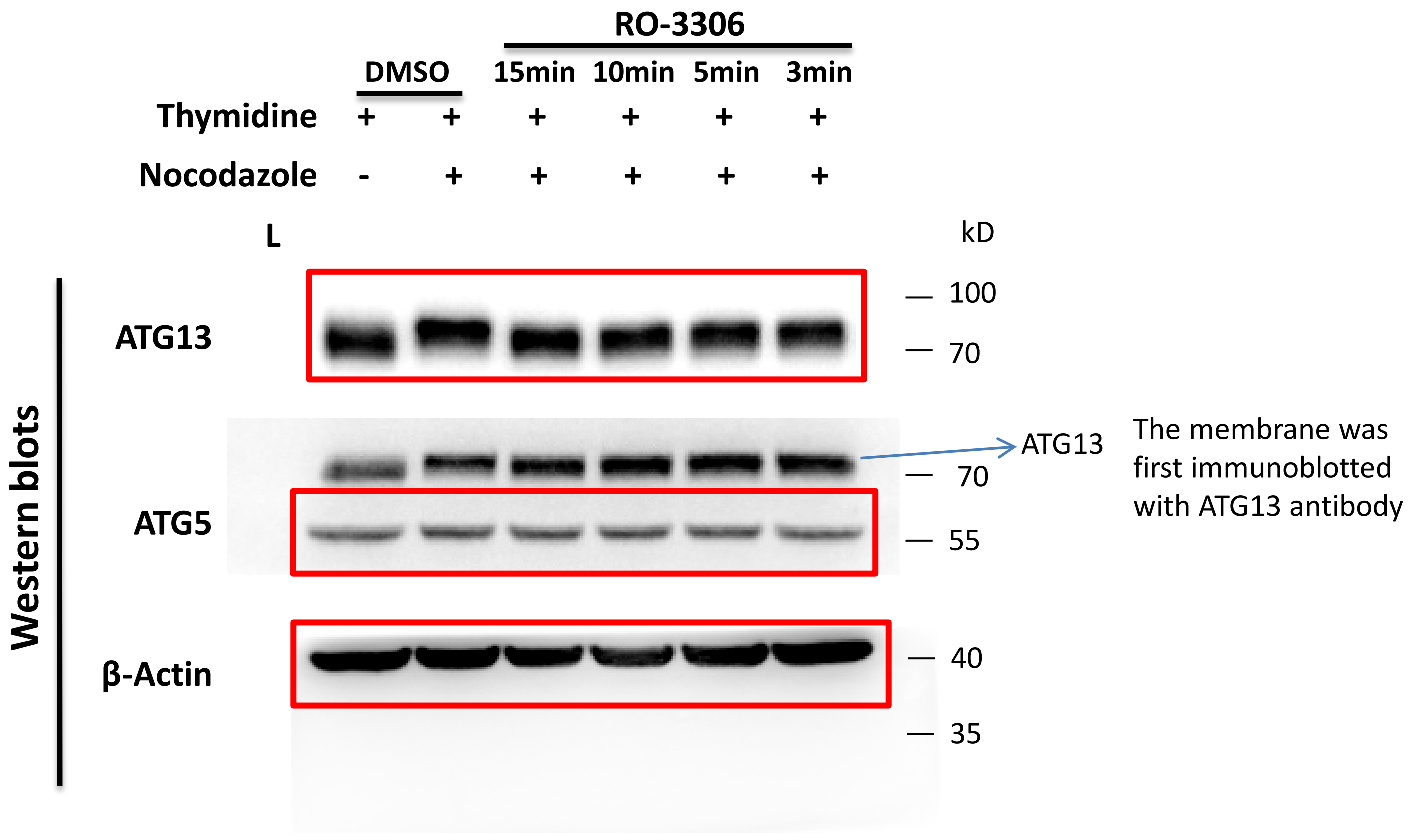

Images were acquired by chemiluminescence CCD camera-based digital imaging instruments (Tanon Fine-do X6).
Approx. molecular weight ladder (L) was indicated.
PVDF Membranes were cut for immunoblotting of more than one protein.

Fig 4D

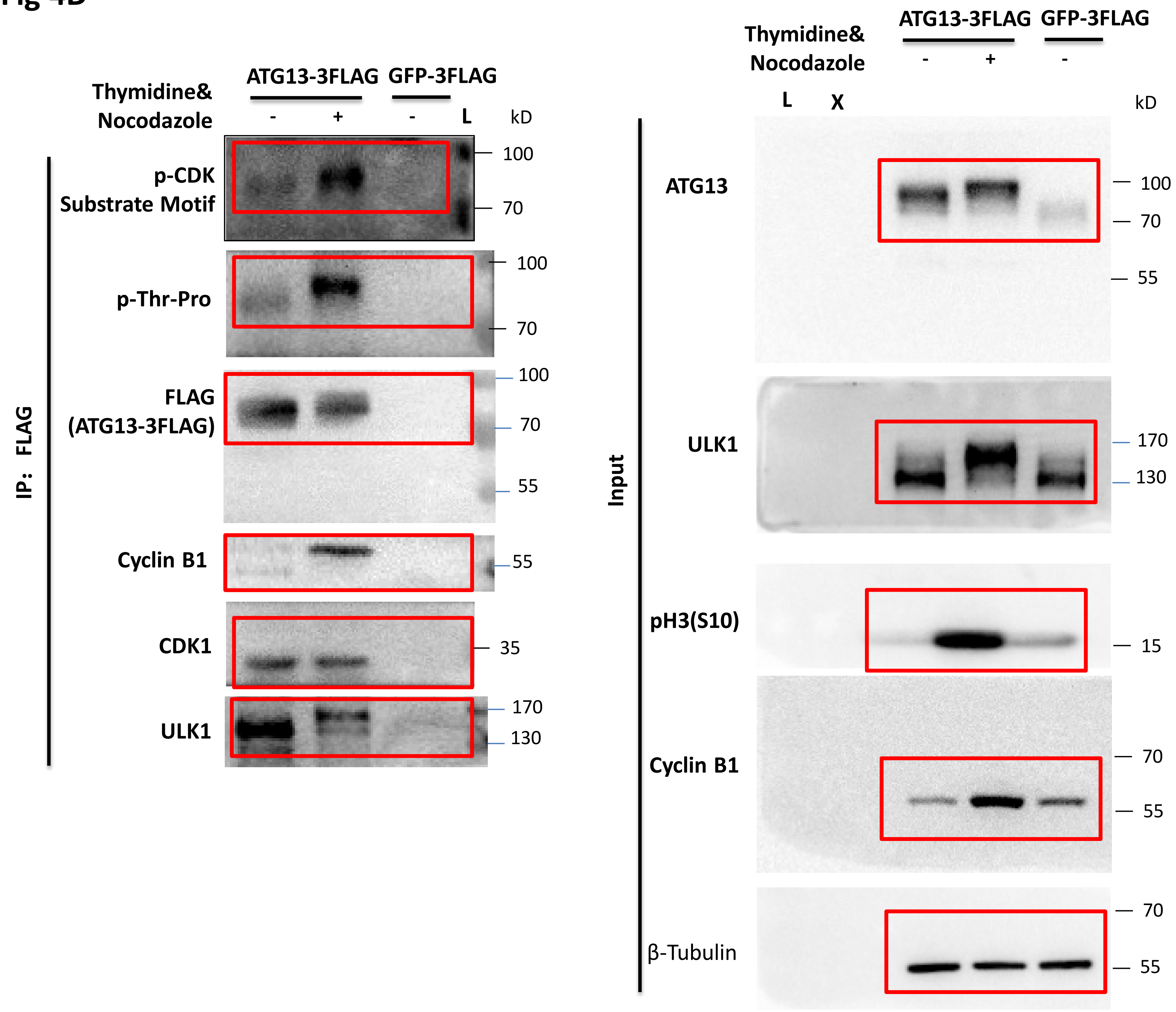

Images were acquired by chemiluminescence CCD camera-based digital imaging instruments (Tanon Fine-do X6).
Approx. molecular weight ladder (L) was indicated.
PVDF Membranes were cut for immunoblotting of more than one protein.

Fig 4E

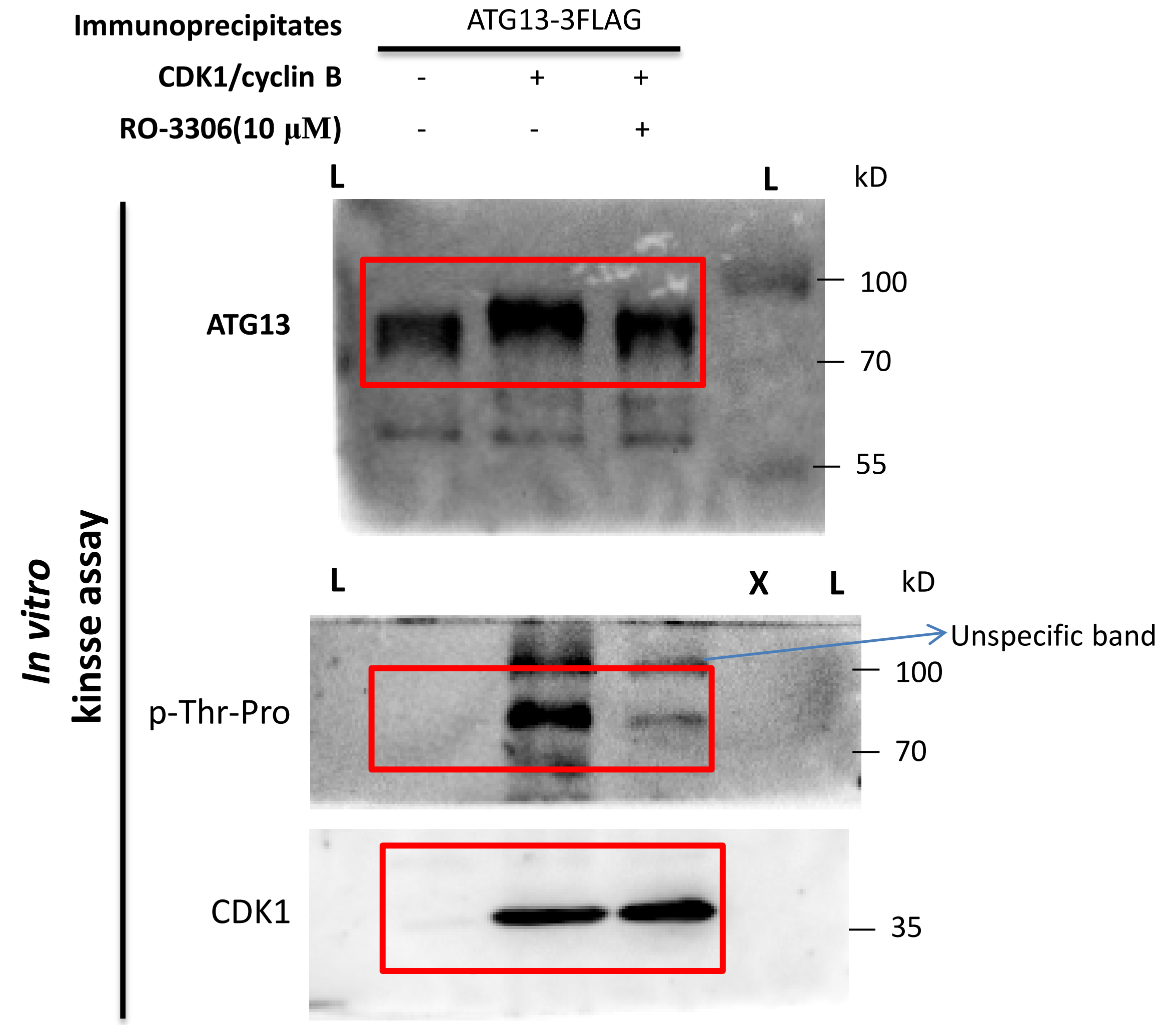

Fig 4F

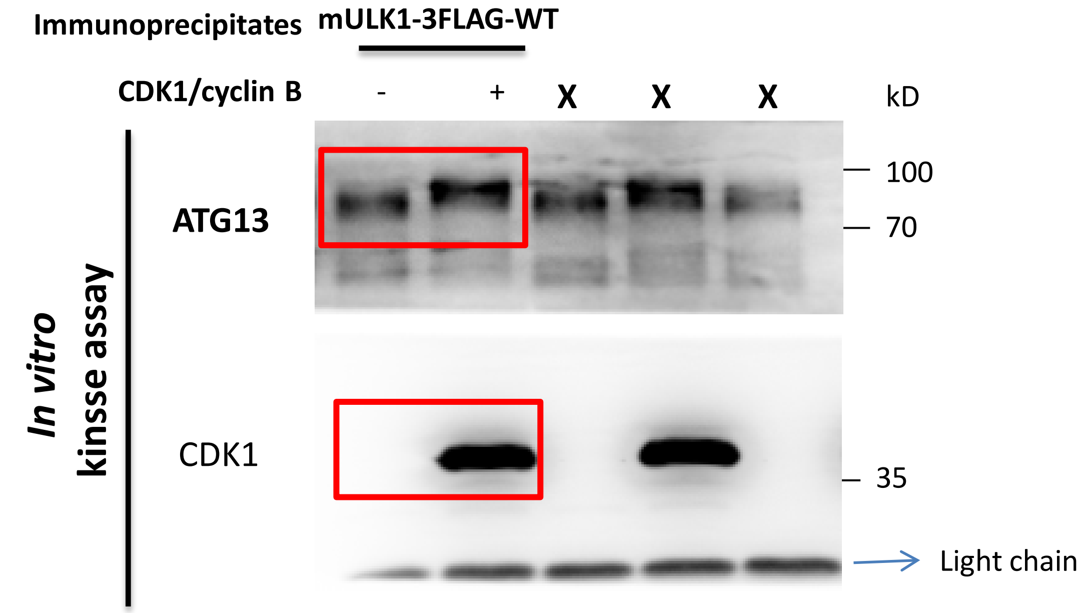

Images were acquired by chemiluminescence CCD camera-based digital imaging instruments (Tanon Fine-do X6).
Approx. molecular weight ladder (L) was indicated.
PVDF Membranes were cut for immunoblotting of more than one protein.

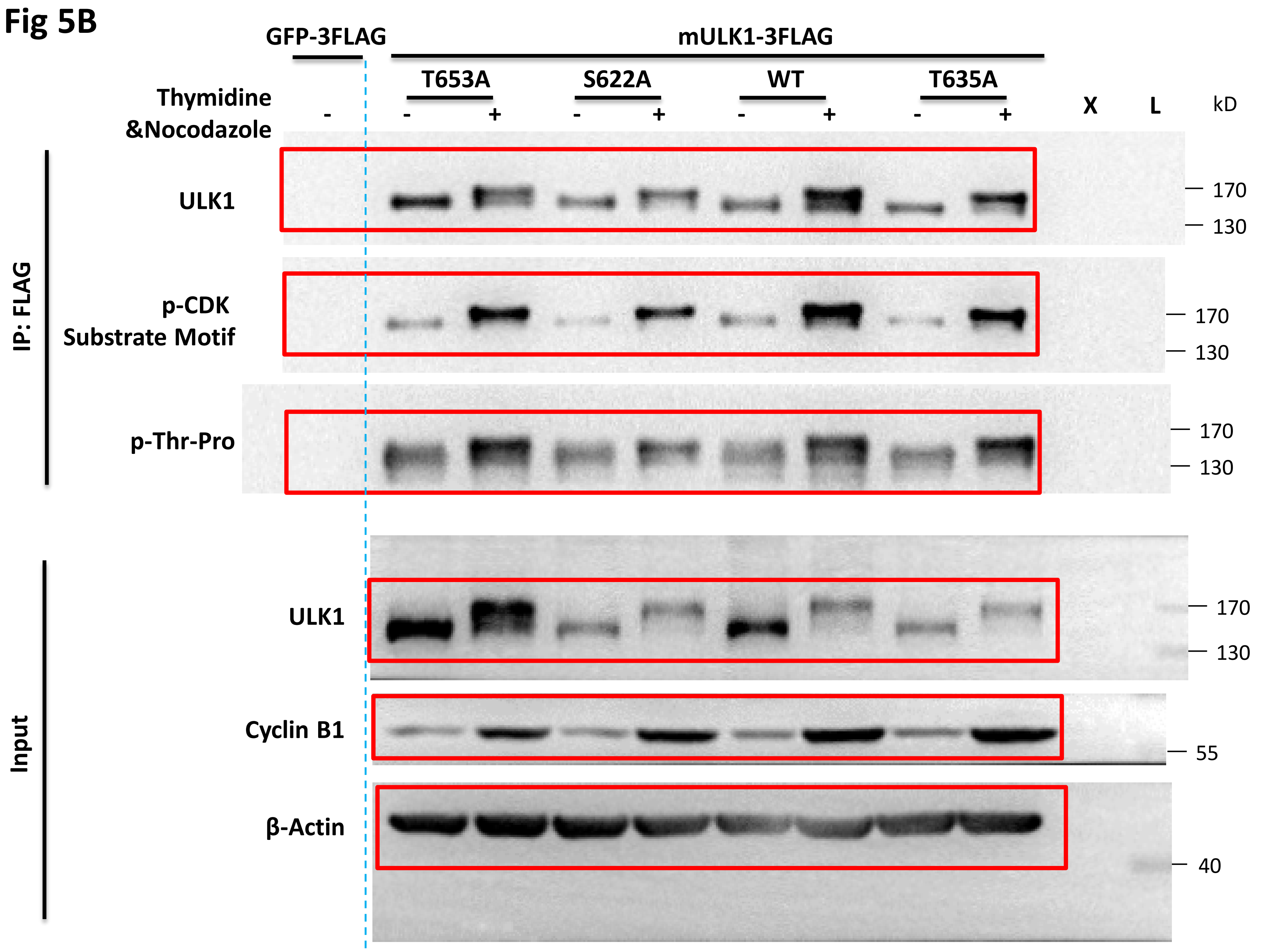

Images were acquired by chemiluminescence CCD camera-based digital imaging instruments (Tanon Fine-do X6).
Approx. molecular weight ladder (L) was indicated.
PVDF Membranes were cut for immunoblotting of more than one protein.

Fig 5C

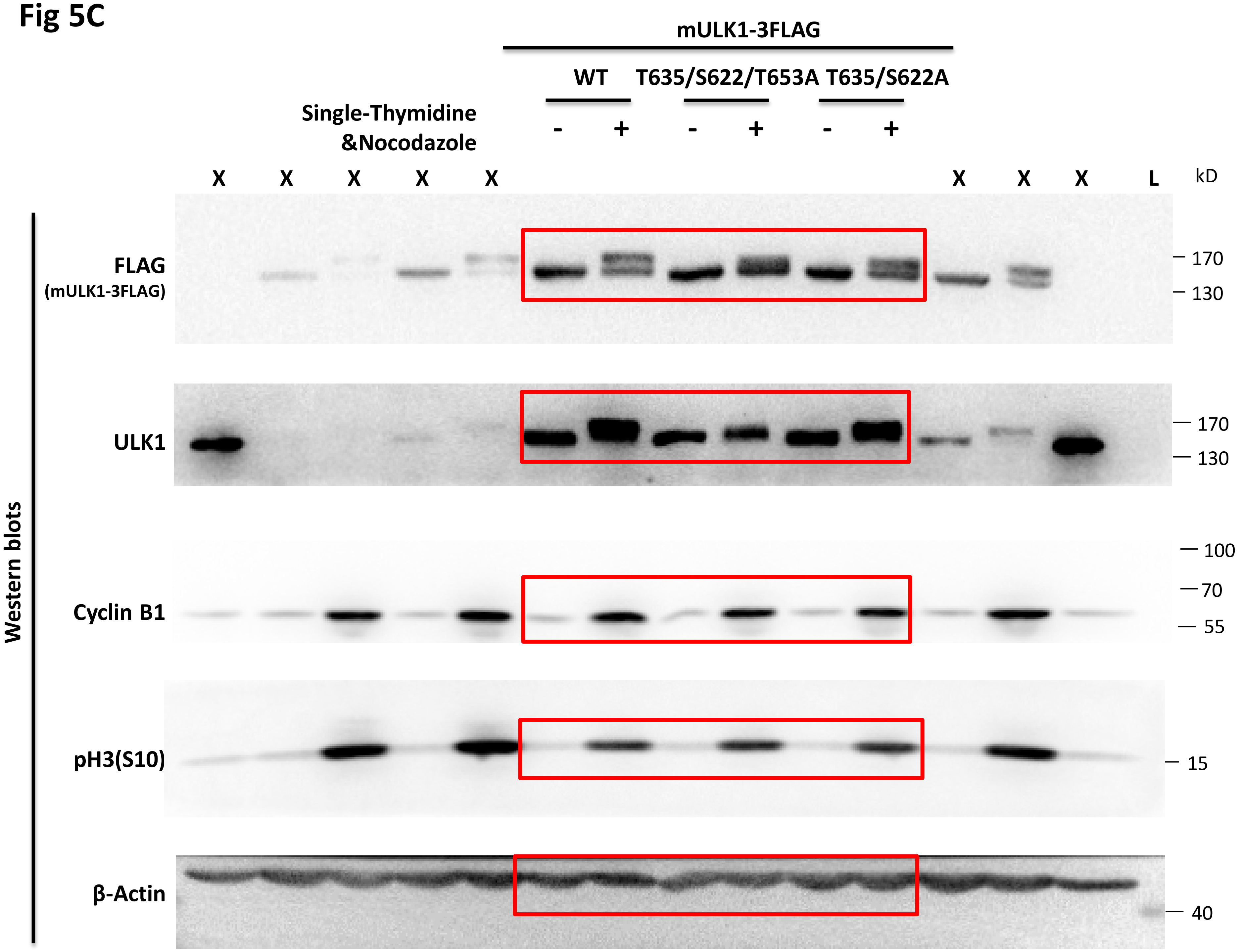

Images were acquired by chemiluminescence CCD camera-based digital imaging instruments (Tanon Fine-do X6).
Approx. molecular weight ladder (L) was indicated.
PVDF Membranes were cut for immunoblotting of more than one protein.

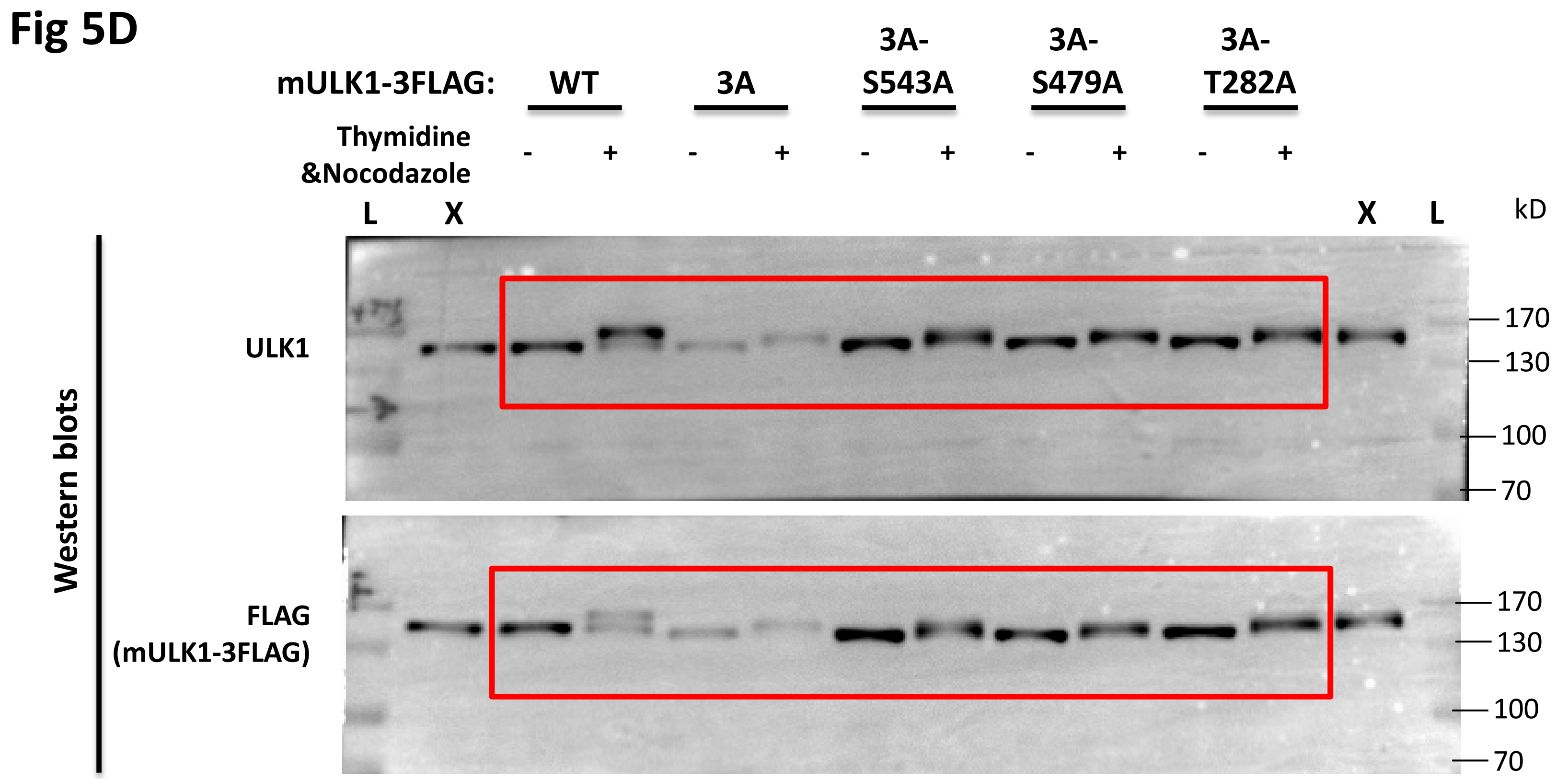

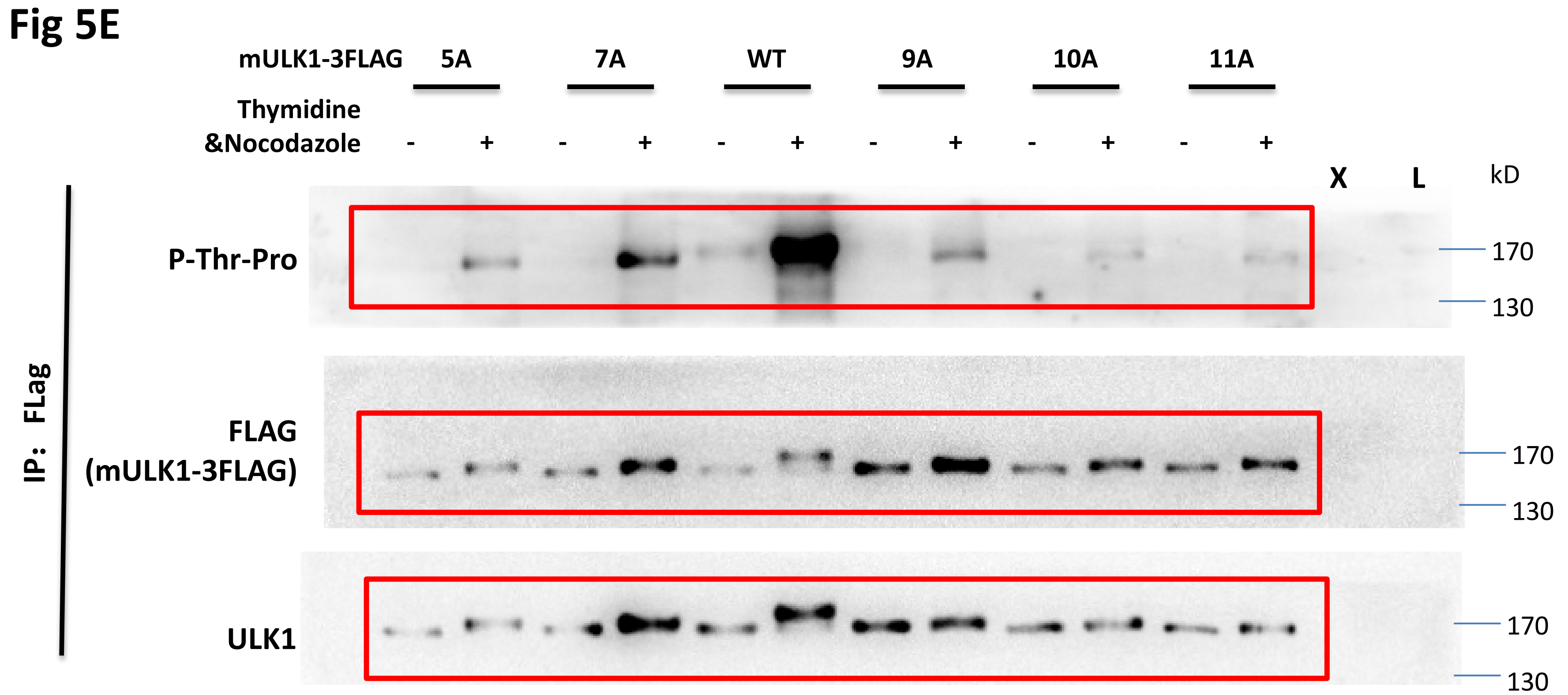

Images were acquired by chemiluminescence CCD camera-based digital imaging instruments (Tanon Fine-do X6).
Approx. molecular weight ladder (L) was indicated.
PVDF Membranes were cut for immunoblotting of more than one protein.

Fig 5F

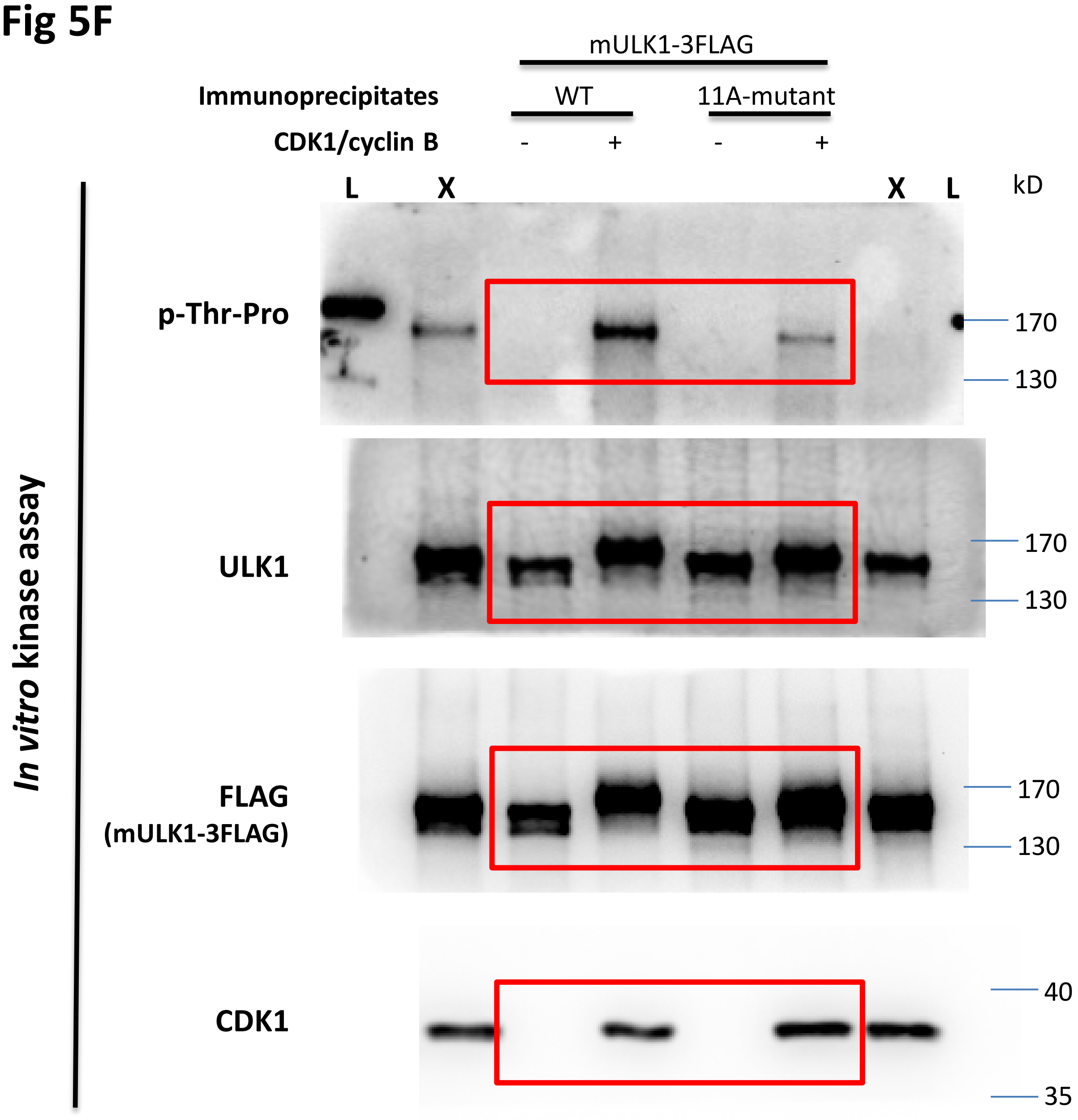

Images were acquired by chemiluminescence CCD camera-based digital imaging instruments (Tanon Fine-do X6).
Approx. molecular weight ladder (L) was indicated.
PVDF Membranes were cut for immunoblotting of more than one protein.

Fig 5H

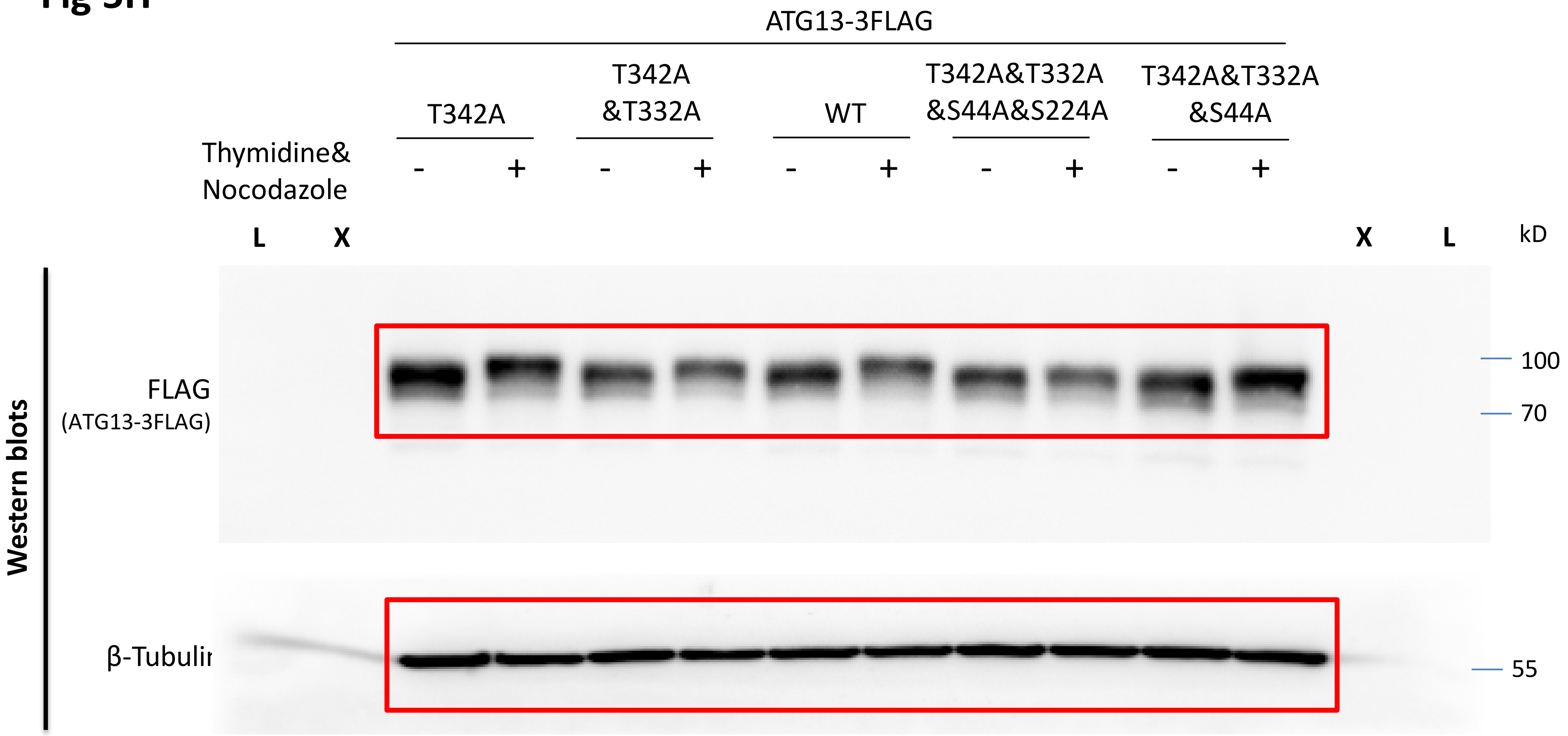

Images were acquired by chemiluminescence CCD camera-based digital imaging instruments (Tanon Fine-do X6).
Approx. molecular weight ladder (L) was indicated.
PVDF Membranes were cut for immunoblotting of more than one protein.

Fig 5I

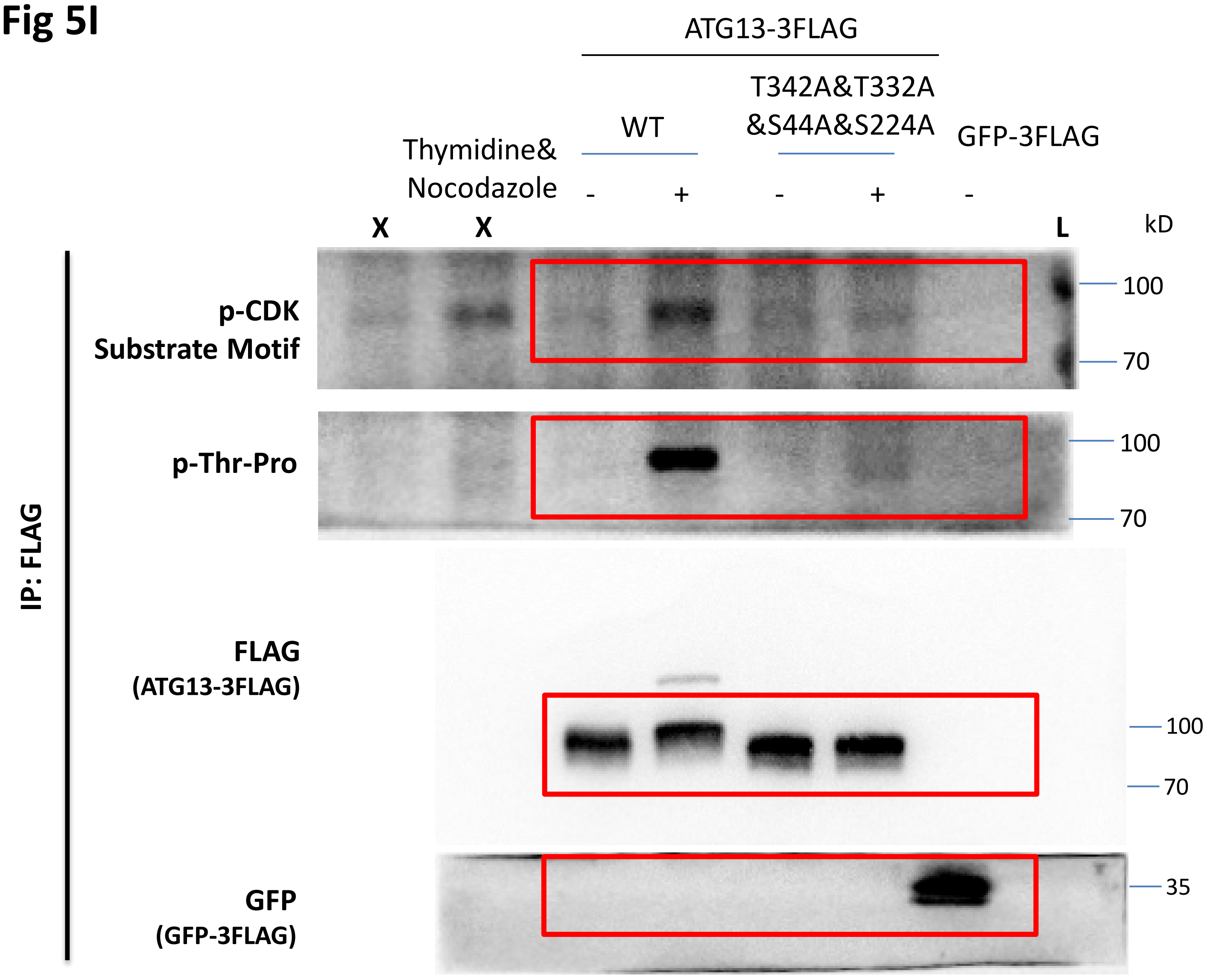

Images were acquired by chemiluminescence CCD camera-based digital imaging instruments (Tanon Fine-do X6).
Approx. molecular weight ladder (L) was indicated.
PVDF Membranes were cut for immunoblotting of more than one protein.

Fig 5I

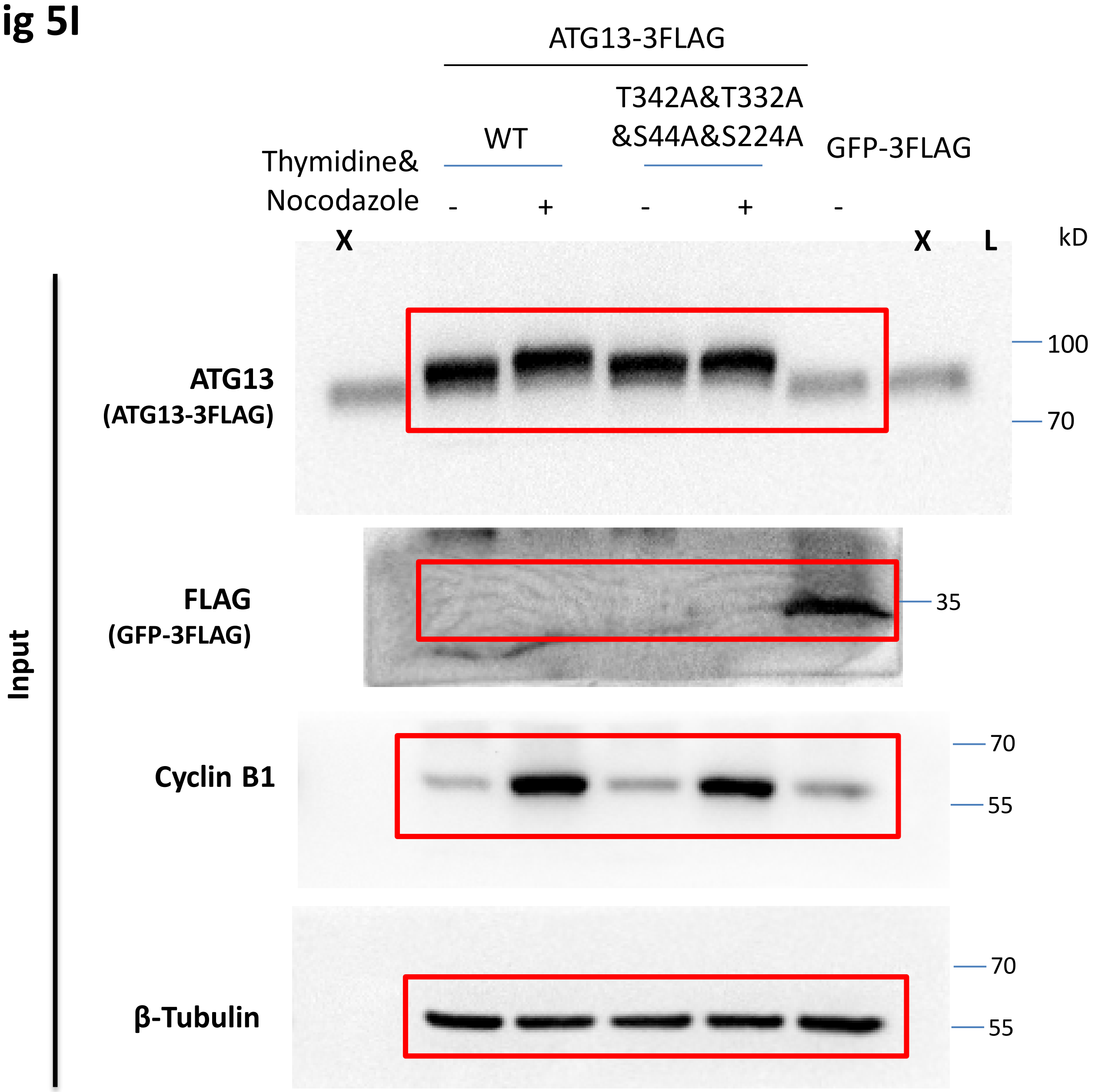

Images were acquired by chemiluminescence CCD camera-based digital imaging instruments (Tanon Fine-do X6).
Approx. molecular weight ladder (L) was indicated.
PVDF Membranes were cut for immunoblotting of more than one protein.

Fig 6B

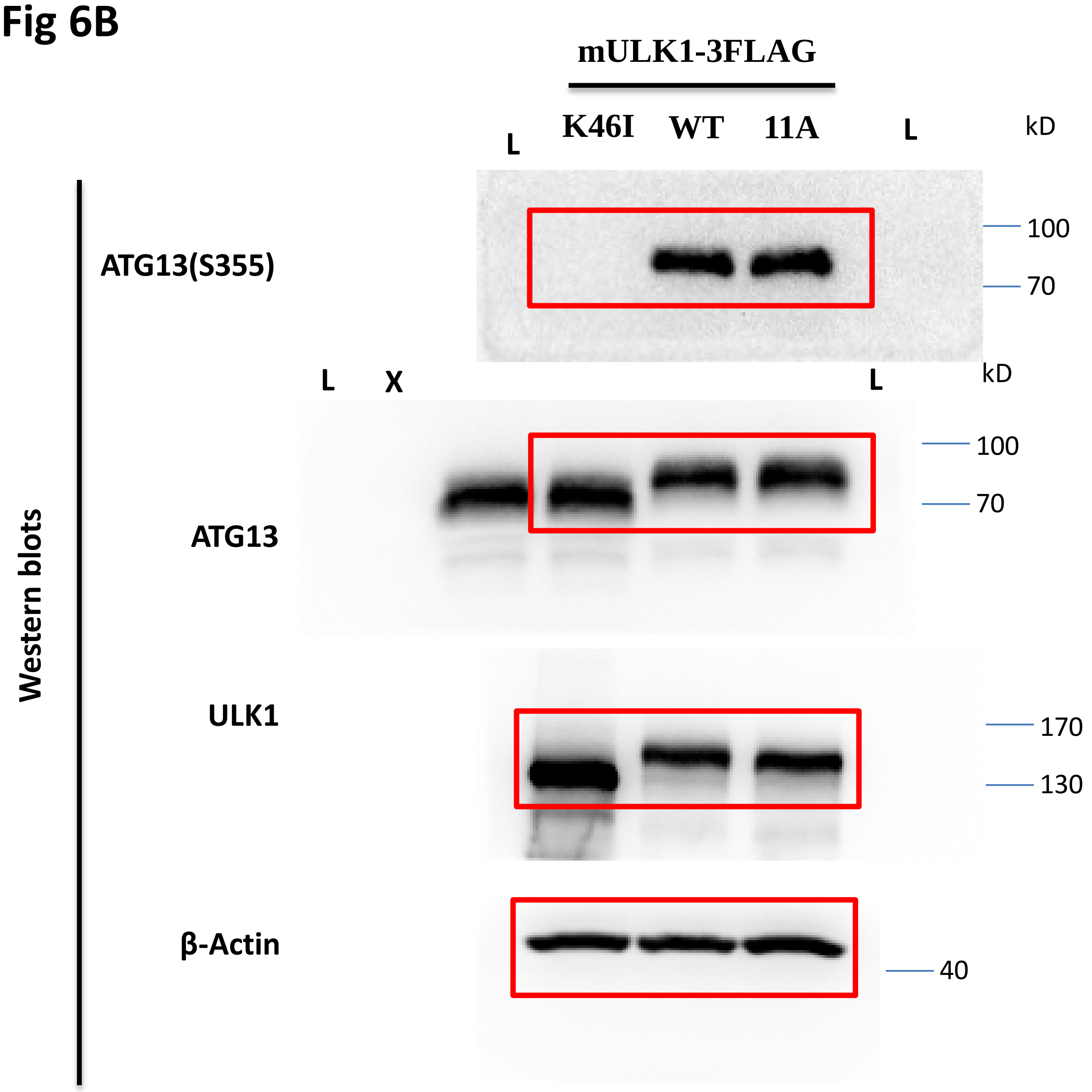

Images were acquired by chemiluminescence CCD camera-based digital imaging instruments (Tanon Fine-do X6).
Approx. molecular weight ladder (L) was indicated.
PVDF Membranes were cut for immunoblotting of more than one protein.

Fig 6D

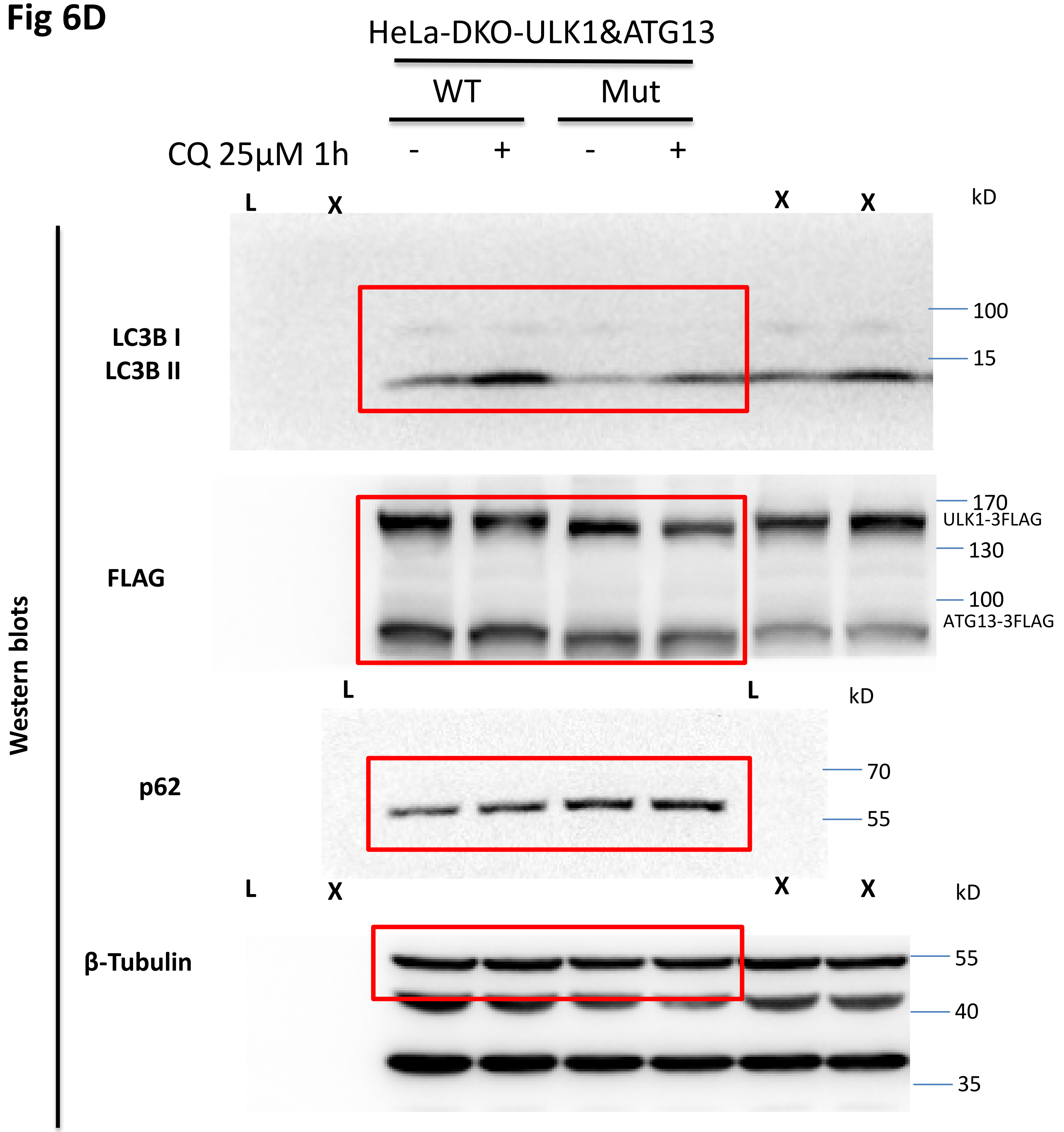

Images were acquired by chemiluminescence CCD camera-based digital imaging instruments (Tanon Fine-do X6).
Approx. molecular weight ladder (L) was indicated.
PVDF Membranes were cut for immunoblotting of more than one protein.

Fig 7B

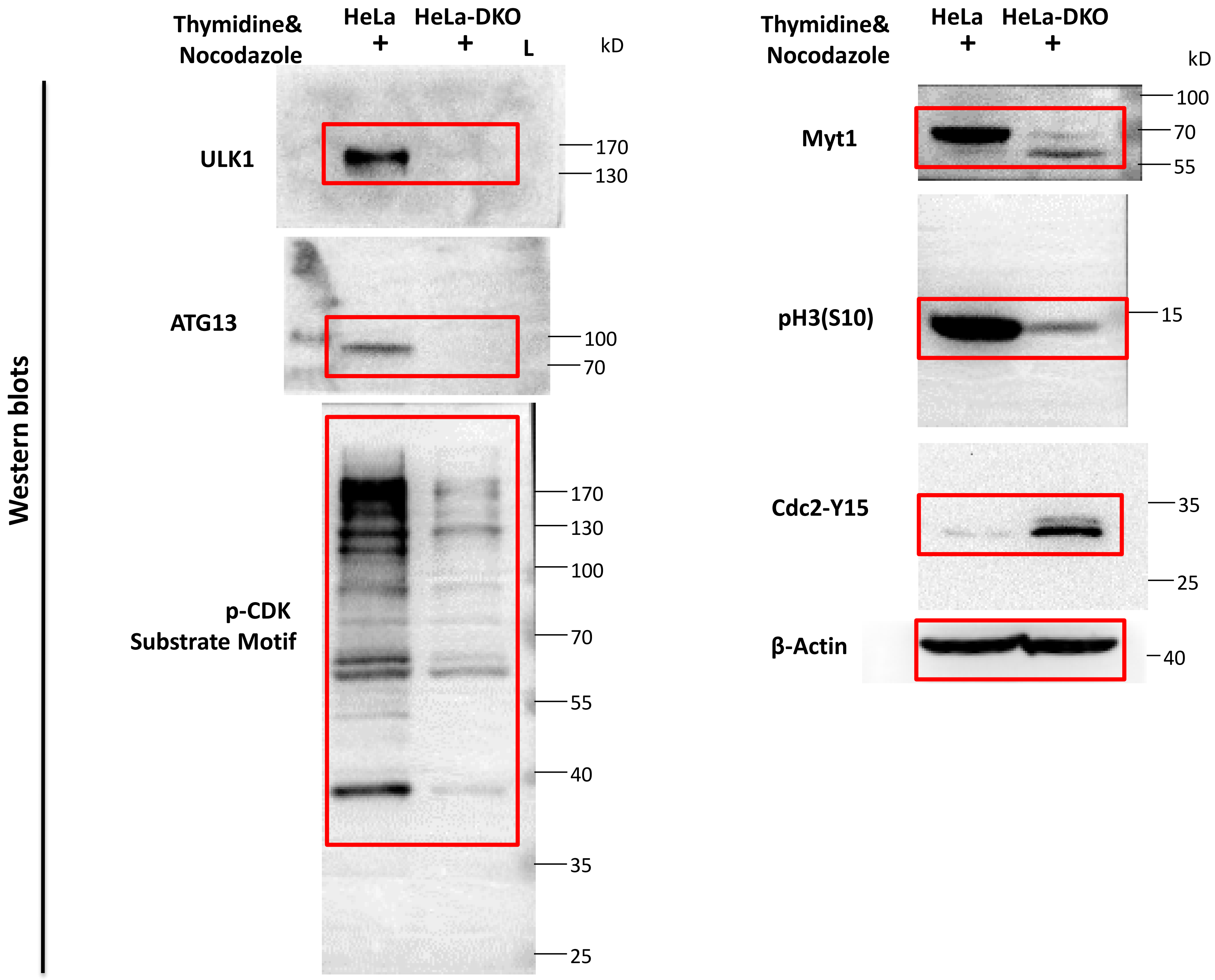

Images were acquired by chemiluminescence CCD camera-based digital imaging instruments (Tanon Fine-do X6).
Approx. molecular weight ladder (L) was indicated.
PVDF Membranes were cut for immunoblotting of more than one protein.

Supplementary Figures

S1A Fig

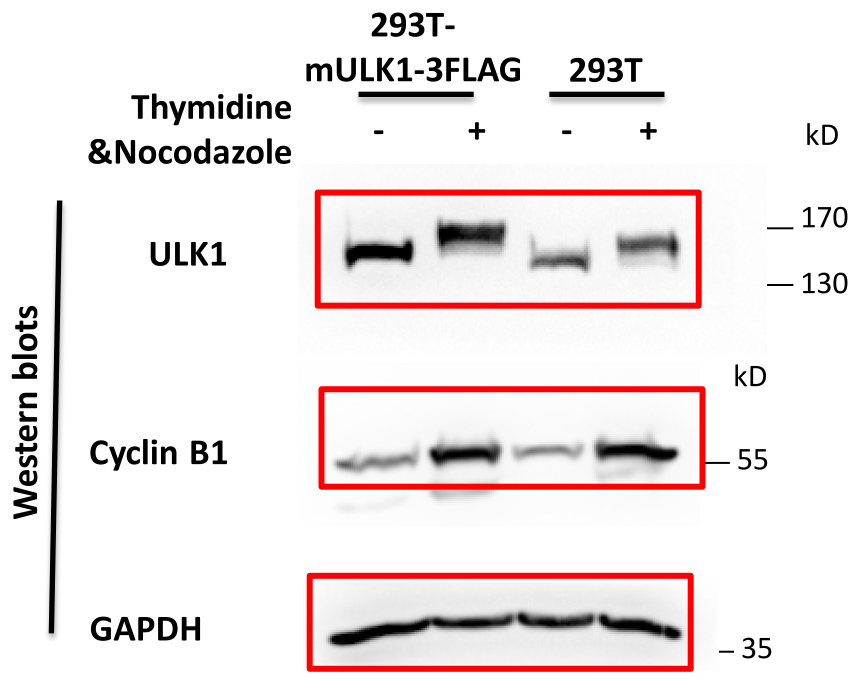

S1B Fig

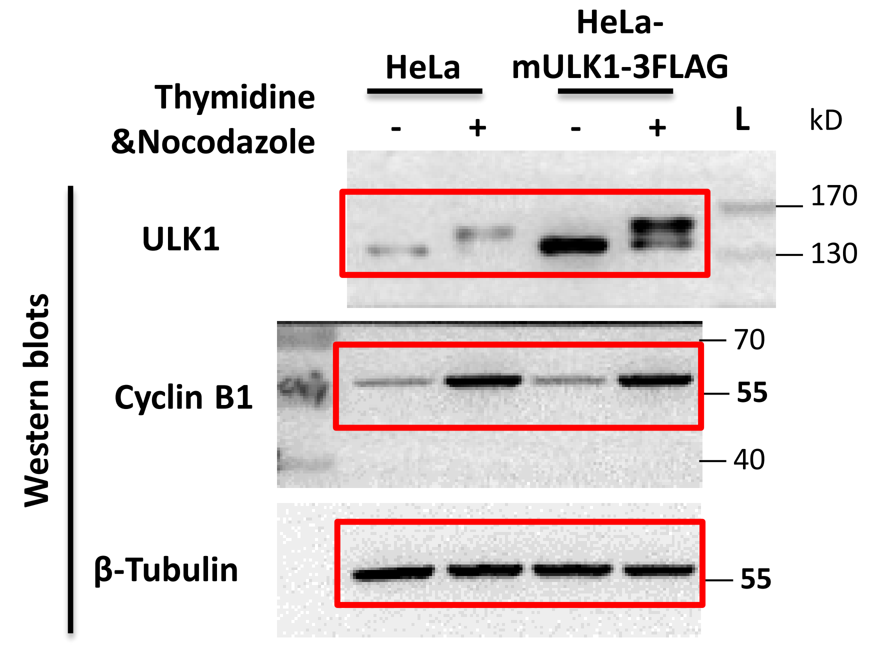

S1C Fig

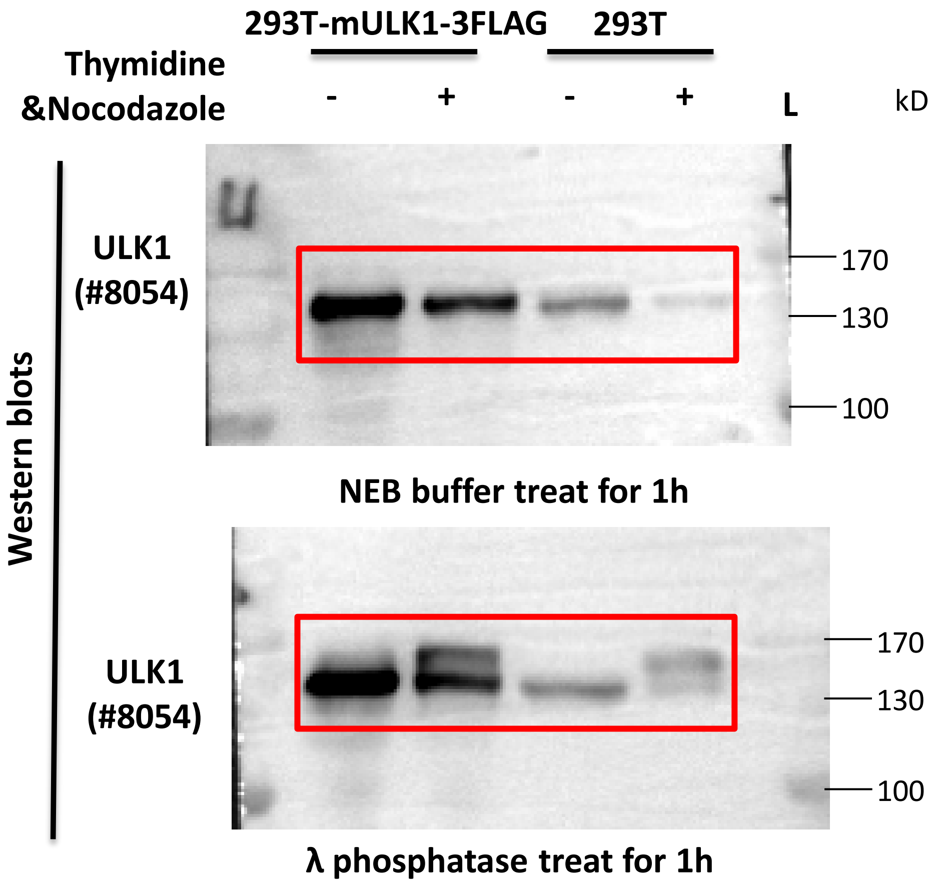

Images were acquired by chemiluminescence CCD camera-based digital imaging instruments (Tanon Fine-do X6).
Approx. molecular weight ladder (L) was indicated.
PVDF Membranes were cut for immunoblotting of more than one protein.

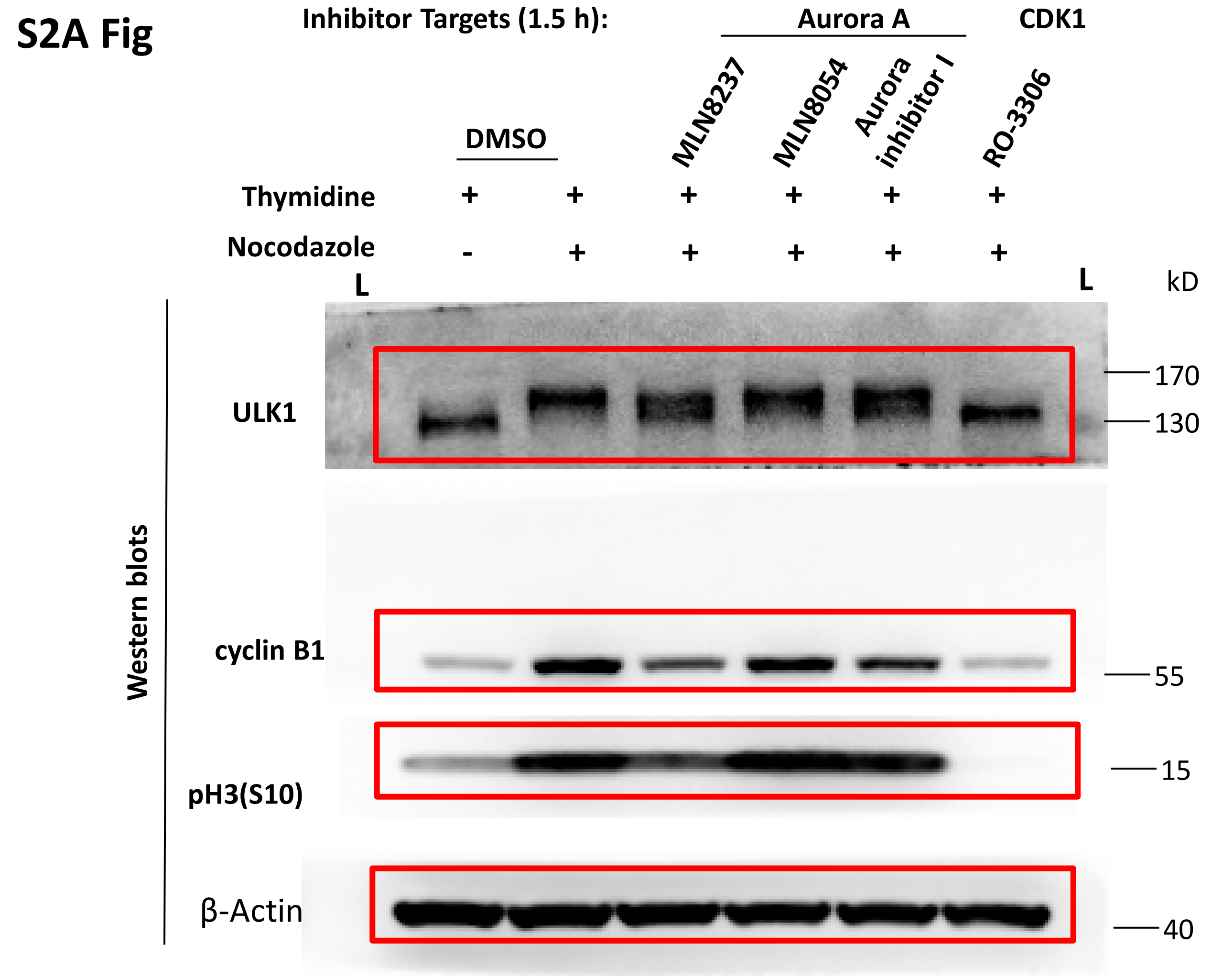

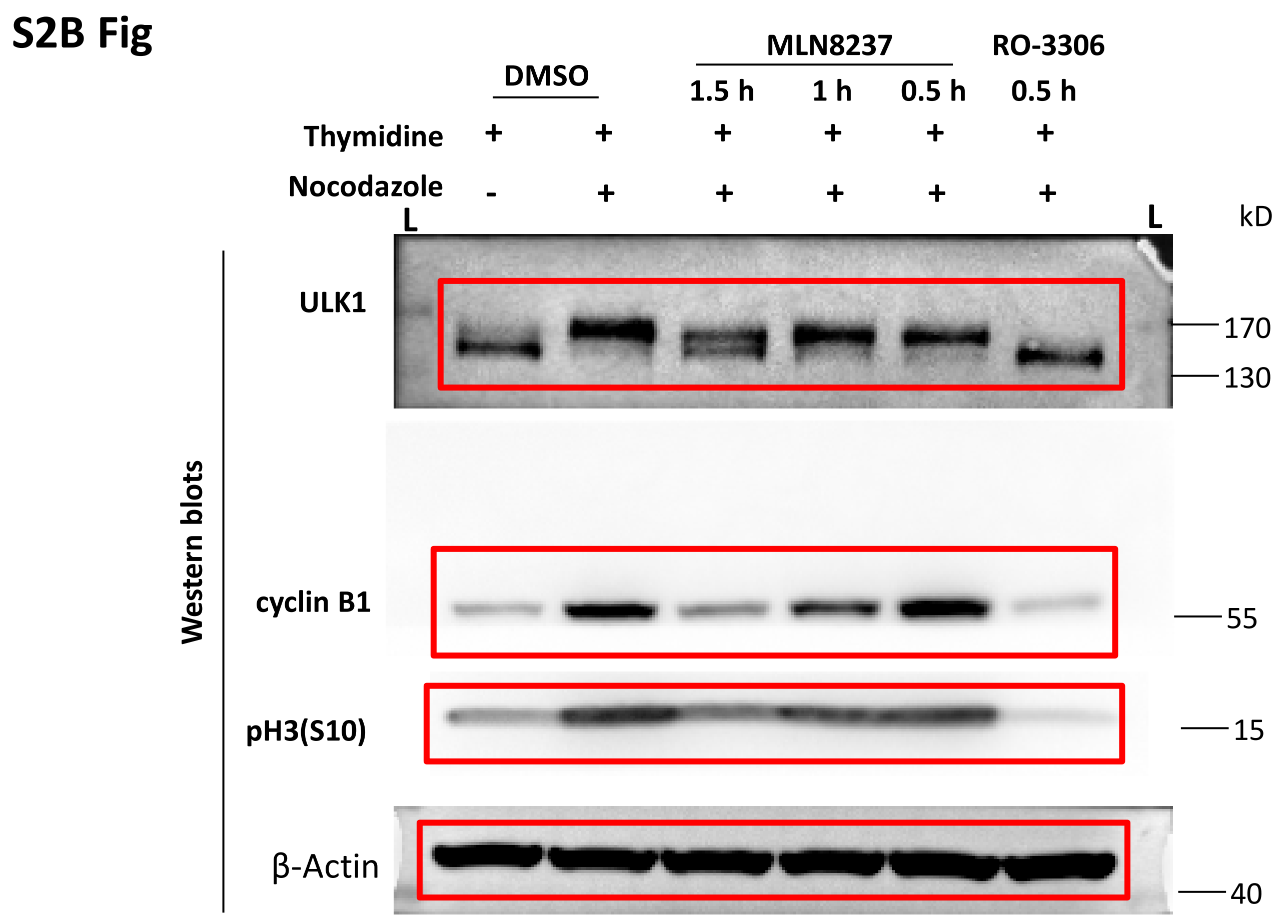

Images were acquired by chemiluminescence CCD camera-based digital imaging instruments (Tanon Fine-do X6).
Approx. molecular weight ladder (L) was indicated.
PVDF Membranes were cut for immunoblotting of more than one protein.

S3A Fig

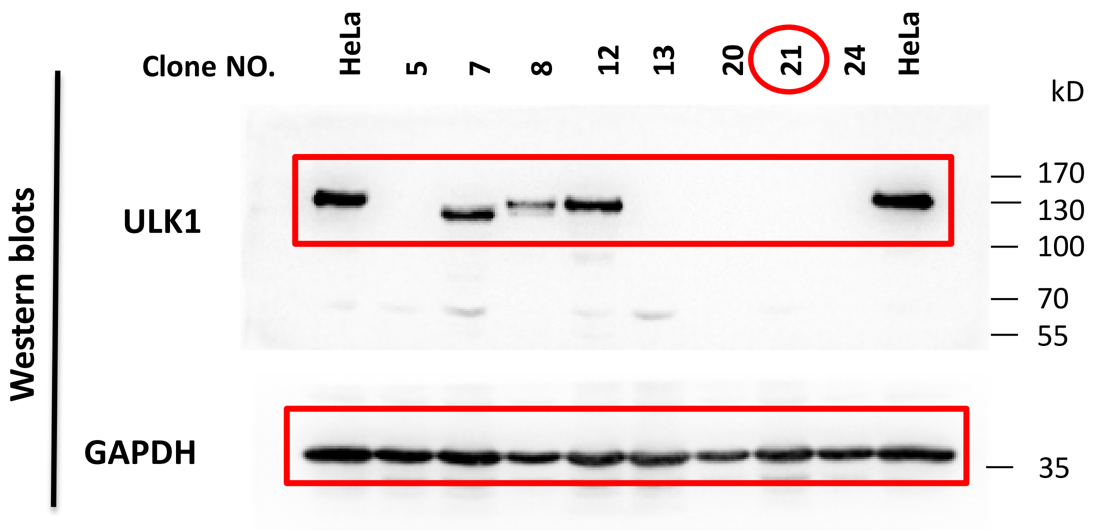

S3B Fig

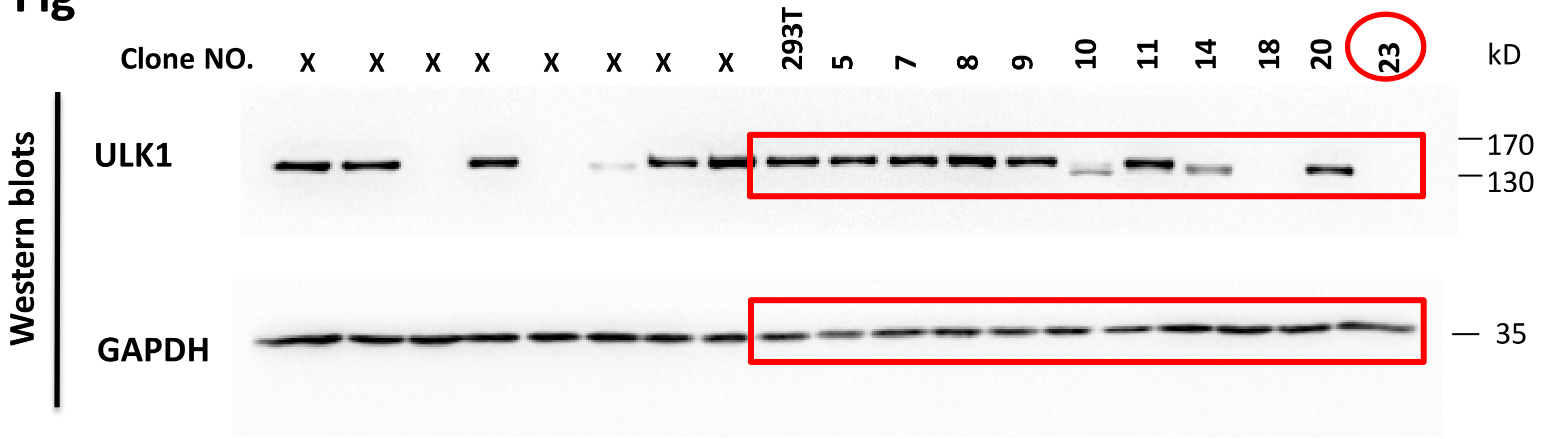

Images were acquired by chemiluminescence CCD camera-based digital imaging instruments (Tanon Fine-do X6).
Approx. molecular weight ladder (L) was indicated.
PVDF Membranes were cut for immunoblotting of more than one protein.

S3C Fig

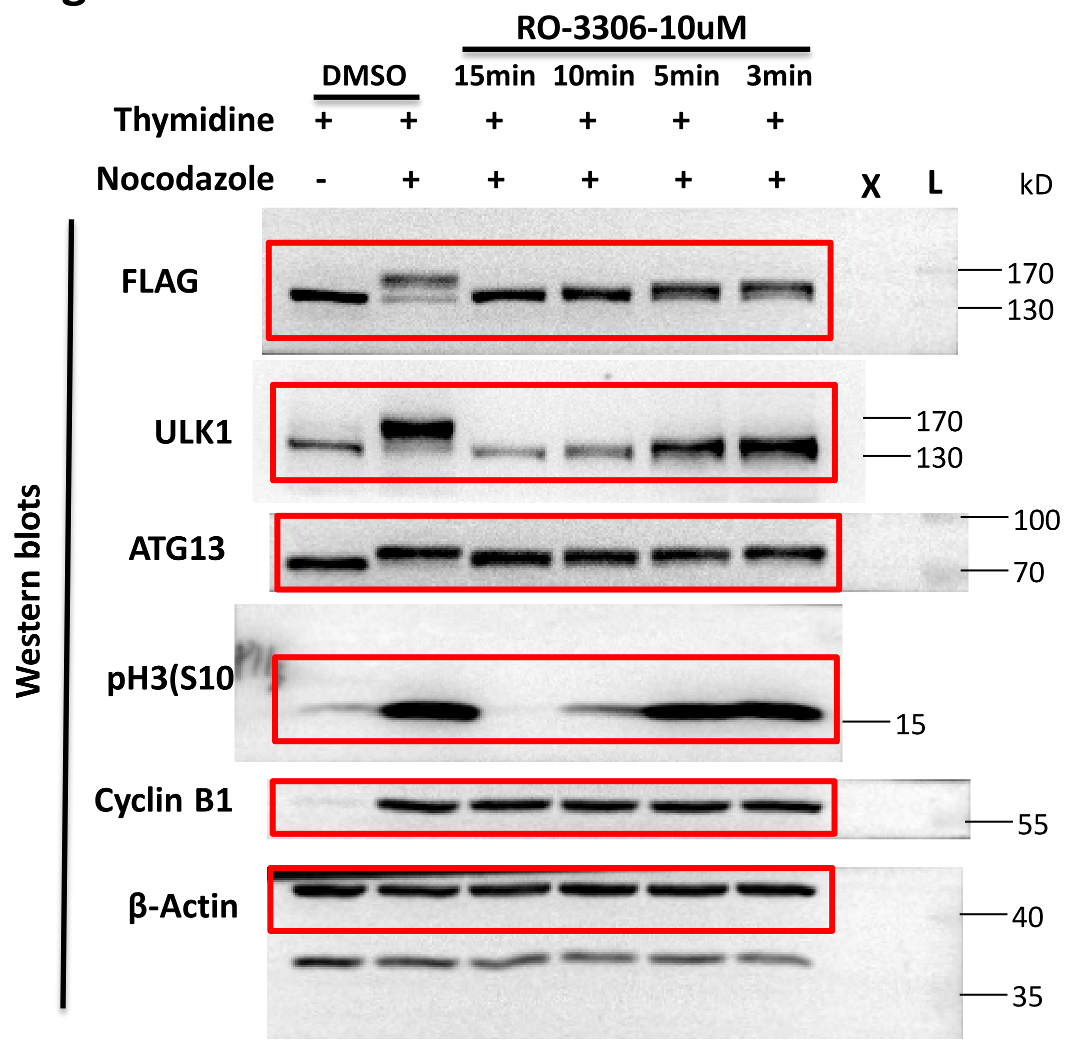

Images were acquired by chemiluminescence CCD camera-based digital imaging instruments (Tanon Fine-do X6).
Approx. molecular weight ladder (L) was indicated.
PVDF Membranes were cut for immunoblotting of more than one protein.

S3D Fig

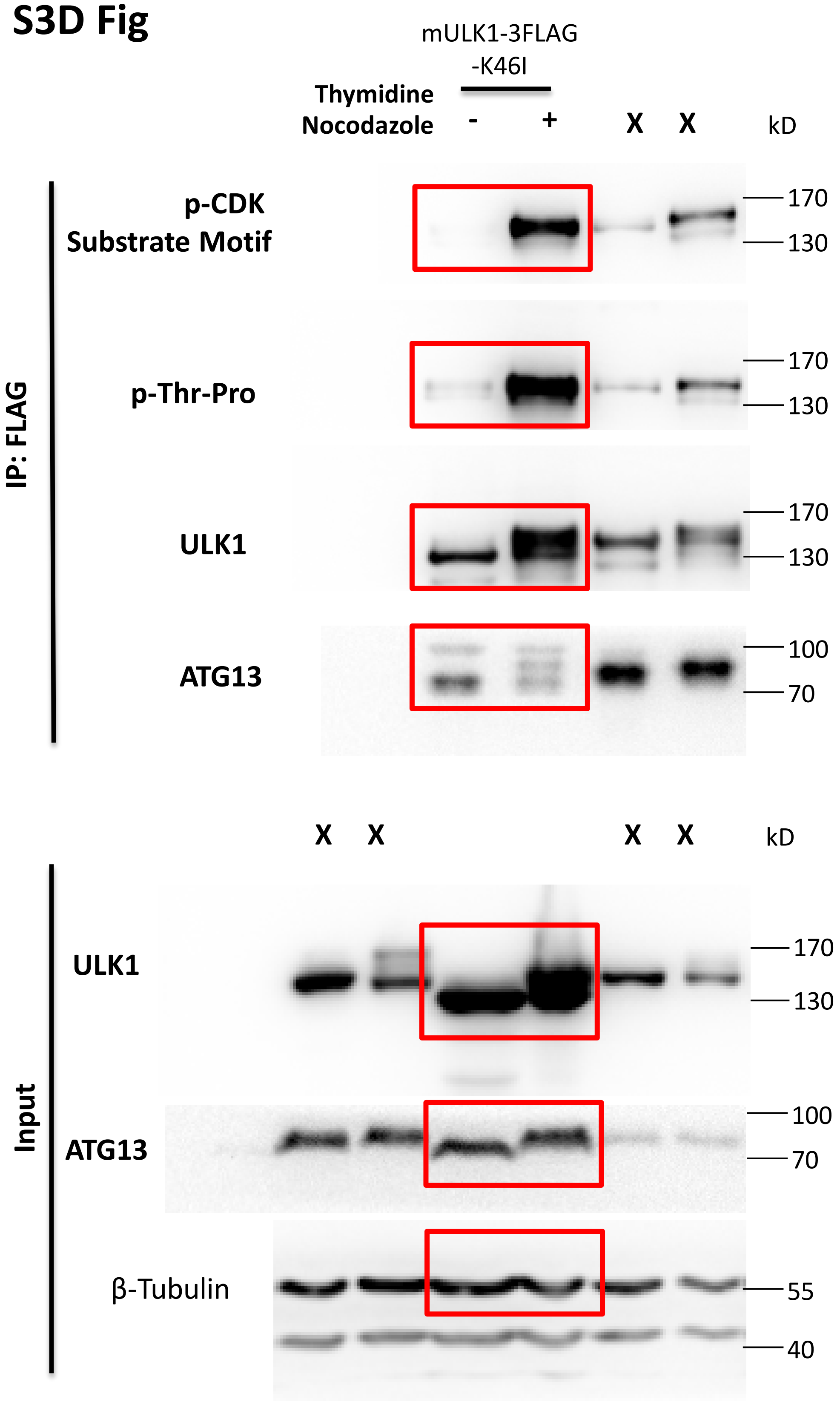

S3E Fig

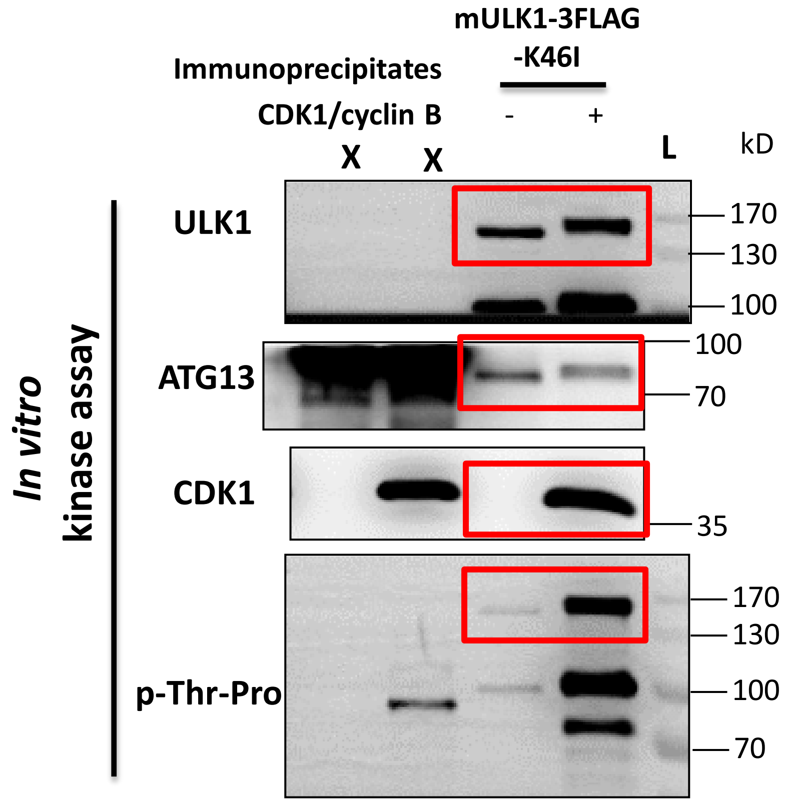

Images were acquired by chemiluminescence CCD camera-based digital imaging instruments (Tanon Fine-do X6).
Approx. molecular weight ladder (L) was indicated.
PVDF Membranes were cut for immunoblotting of more than one protein.

S4 Fig

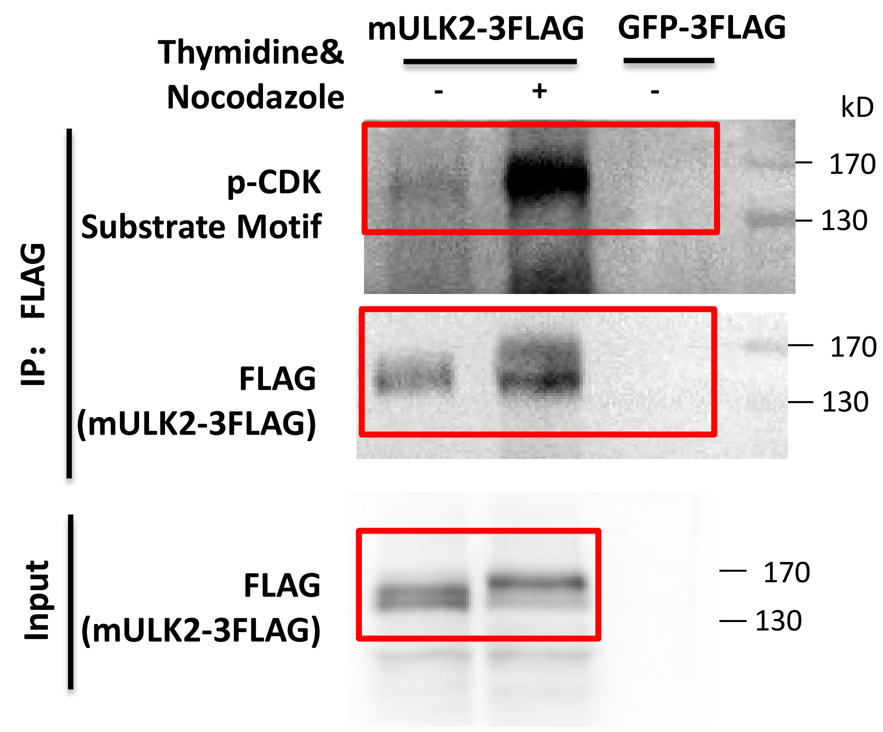

Images were acquired by chemiluminescence CCD camera-based digital imaging instruments (Tanon Fine-do X6).
Approx. molecular weight ladder (L) was indicated.
PVDF Membranes were cut for immunoblotting of more than one protein.

S5A Fig

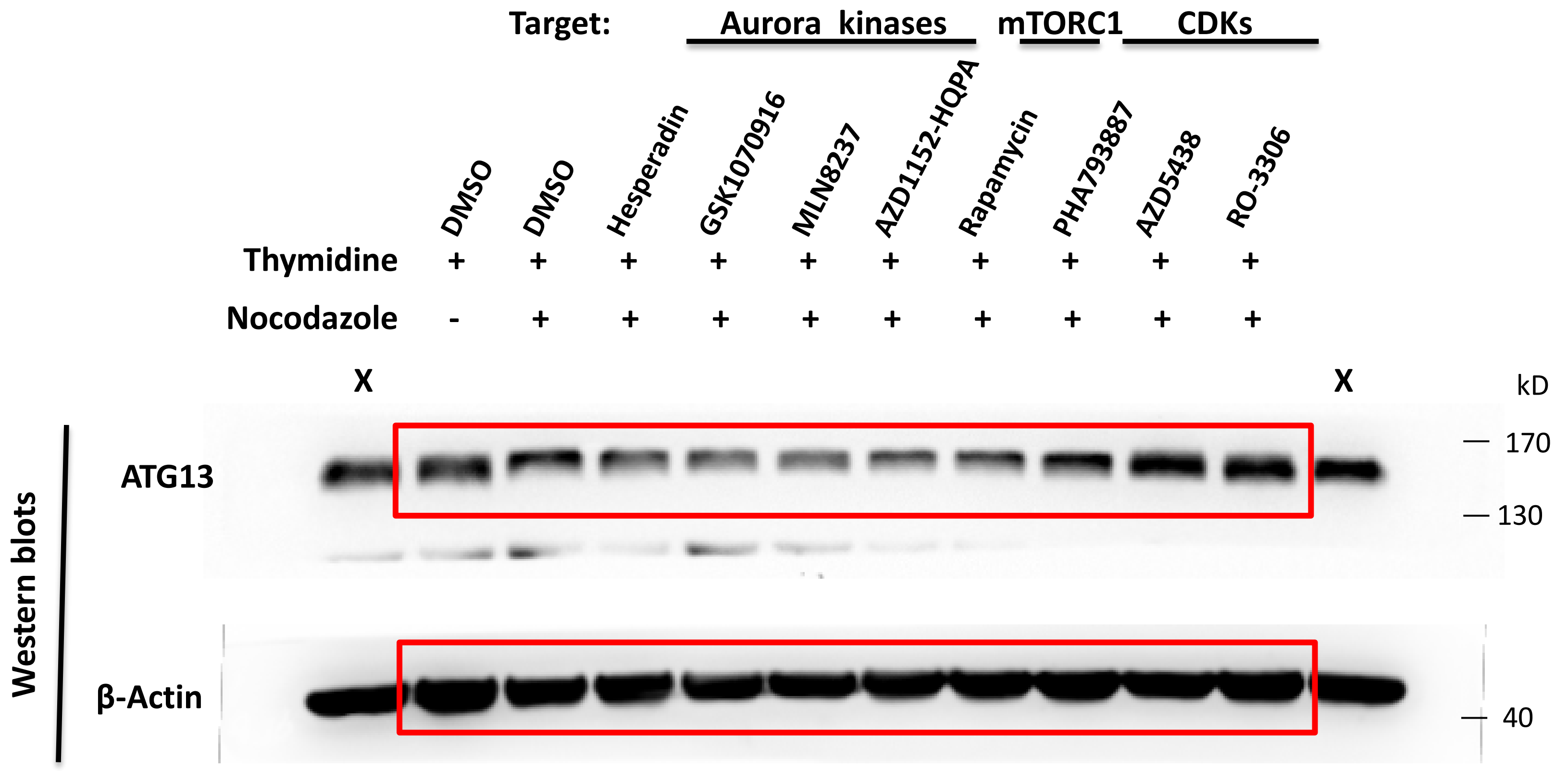

Images were acquired by chemiluminescence CCD camera-based digital imaging instruments (Tanon Fine-do X6).
Approx. molecular weight ladder (L) was indicated.
PVDF Membranes were cut for immunoblotting of more than one protein.

S5B Fig

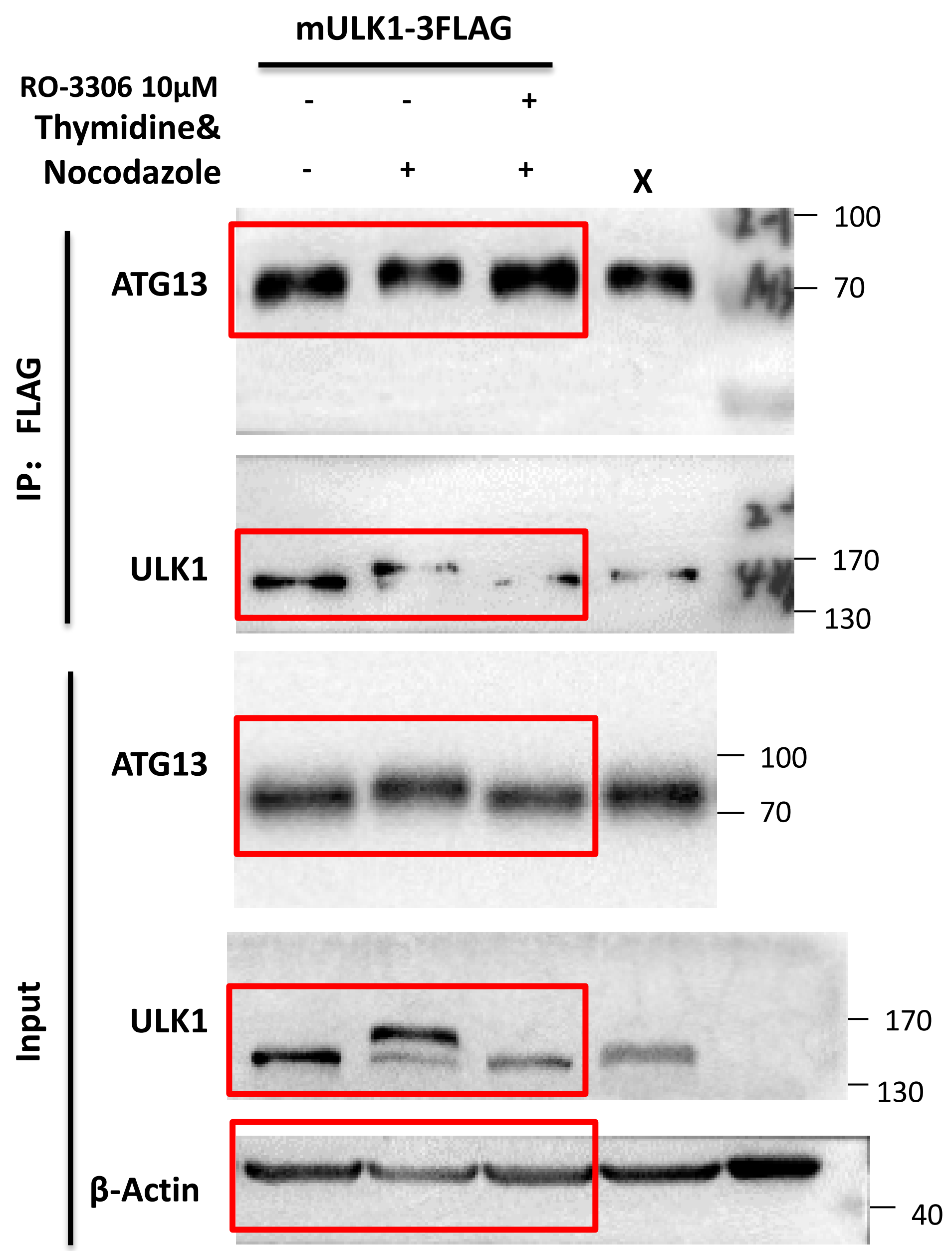

Images were acquired by chemiluminescence CCD camera-based digital imaging instruments (Tanon Fine-do X6).
Approx. molecular weight ladder (L) was indicated.
PVDF Membranes were cut for immunoblotting of more than one protein.

S5C Fig

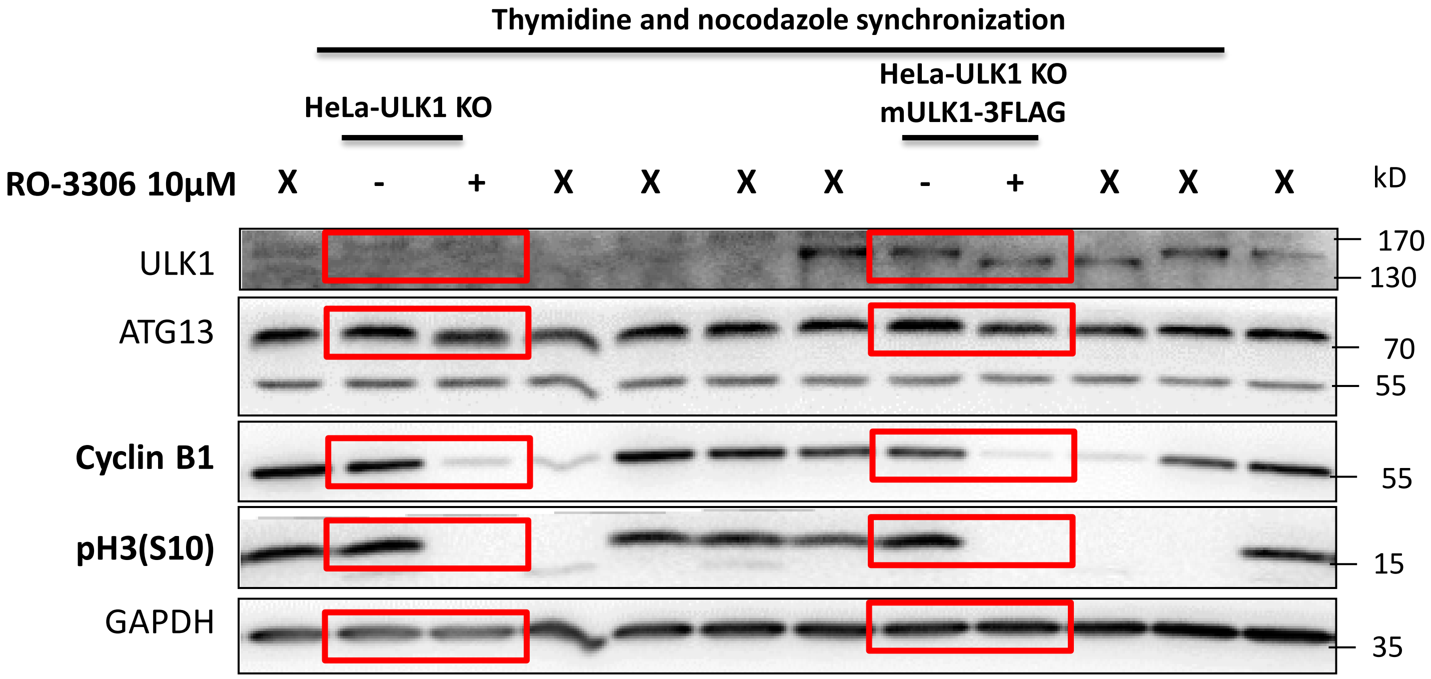

Images were acquired by chemiluminescence CCD camera-based digital imaging instruments (Tanon Fine-do X6).
Approx. molecular weight ladder (L) was indicated.
PVDF Membranes were cut for immunoblotting of more than one protein.

S5D Fig

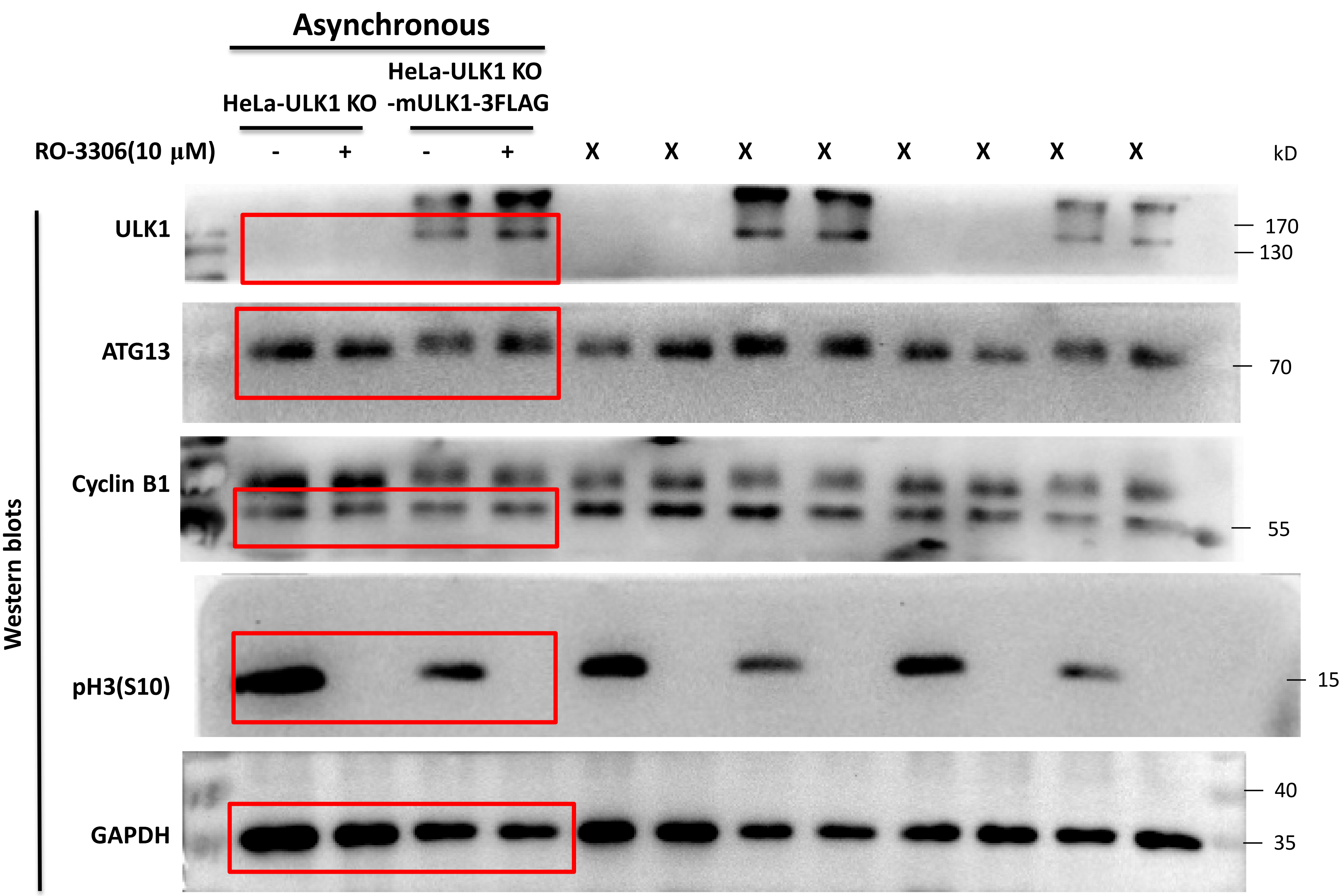

Images were acquired by chemiluminescence CCD camera-based digital imaging instruments (Tanon Fine-do X6).
Approx. molecular weight ladder (L) was indicated.
PVDF Membranes were cut for immunoblotting of more than one protein.

S6A Fig

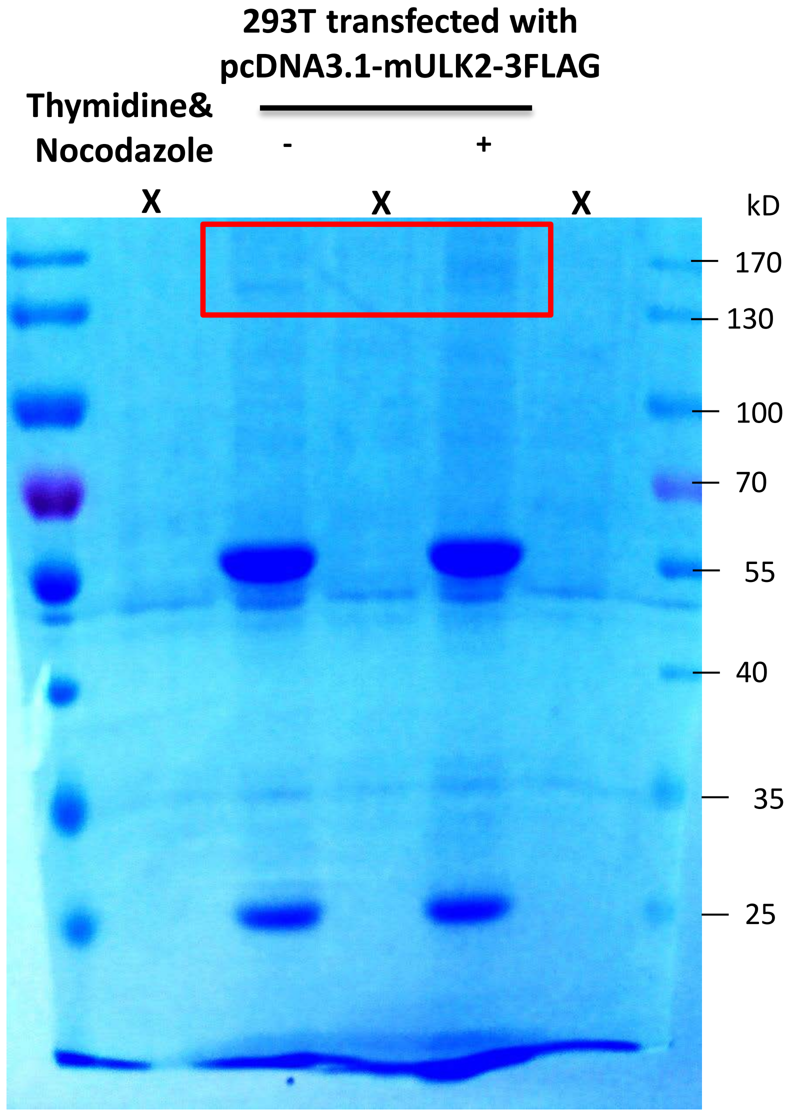

S6C Fig

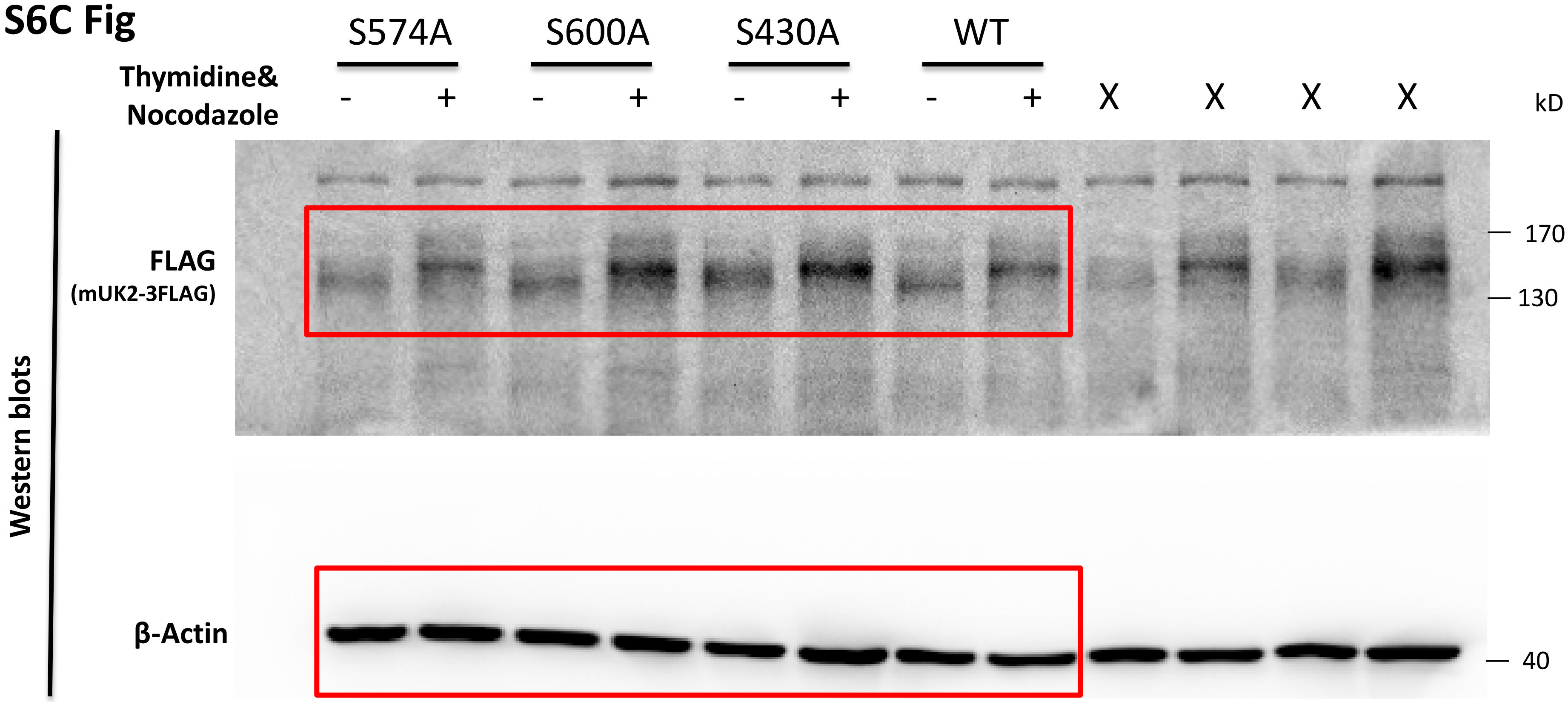

Images were acquired by chemiluminescence CCD camera-based digital imaging instruments (Tanon Fine-do X6).
Approx. molecular weight ladder (L) was indicated.
PVDF Membranes were cut for immunoblotting of more than one protein.

S7 Fig

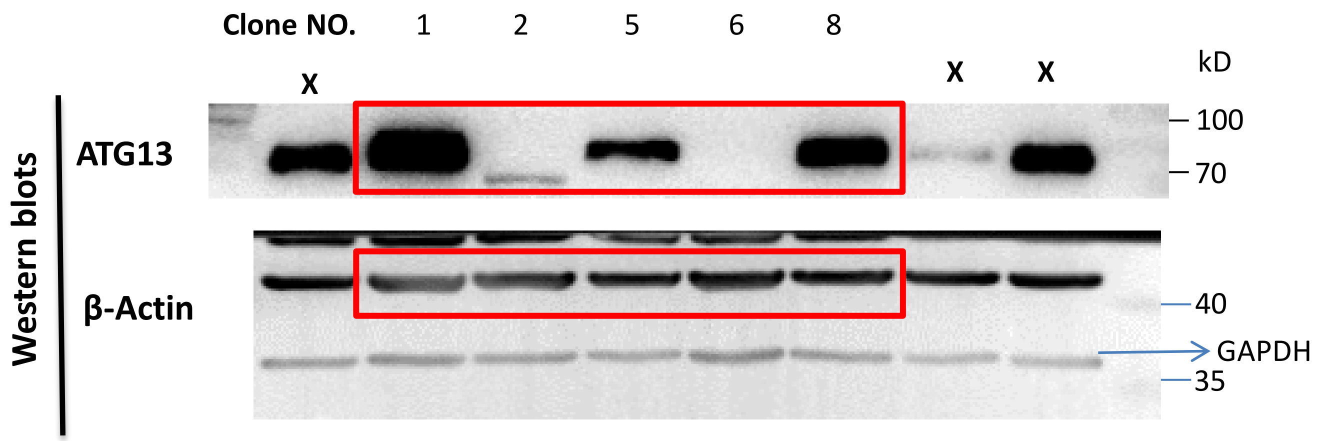

S10A Fig

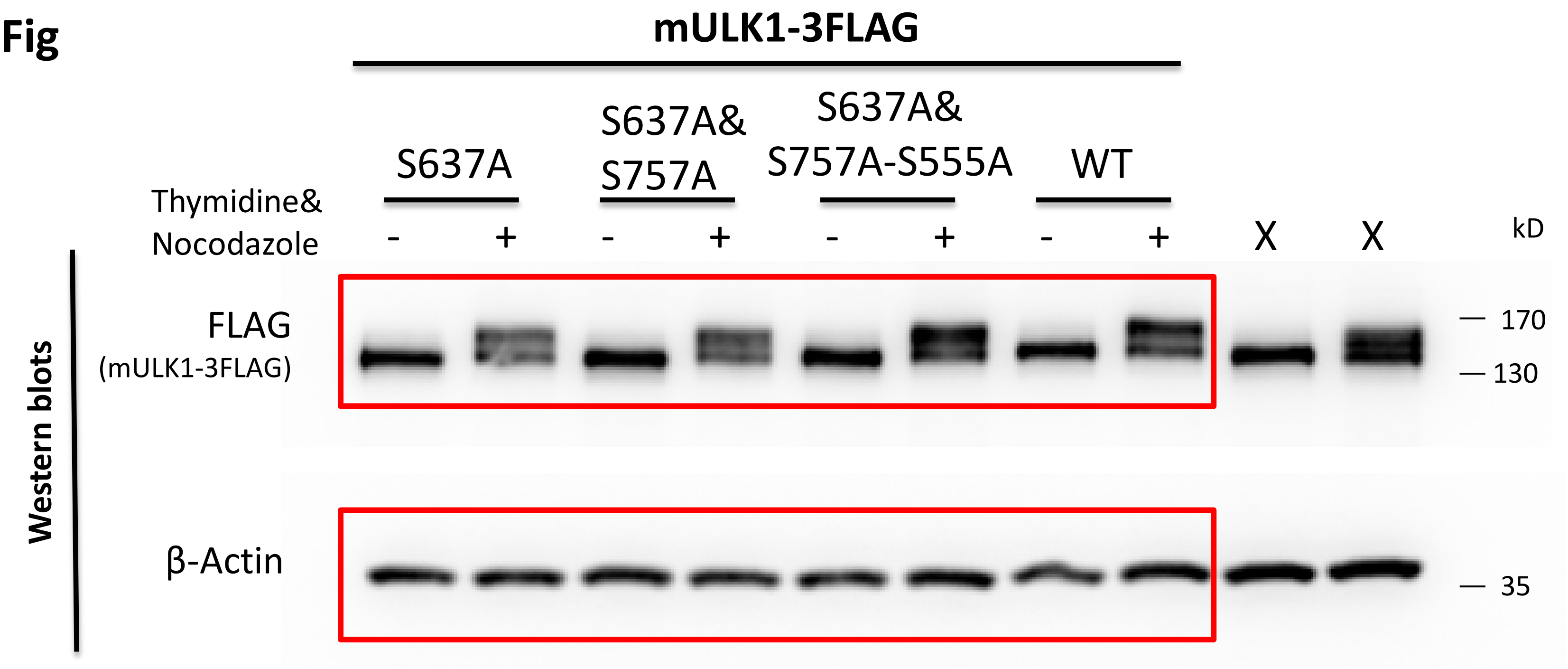

S10B Fig

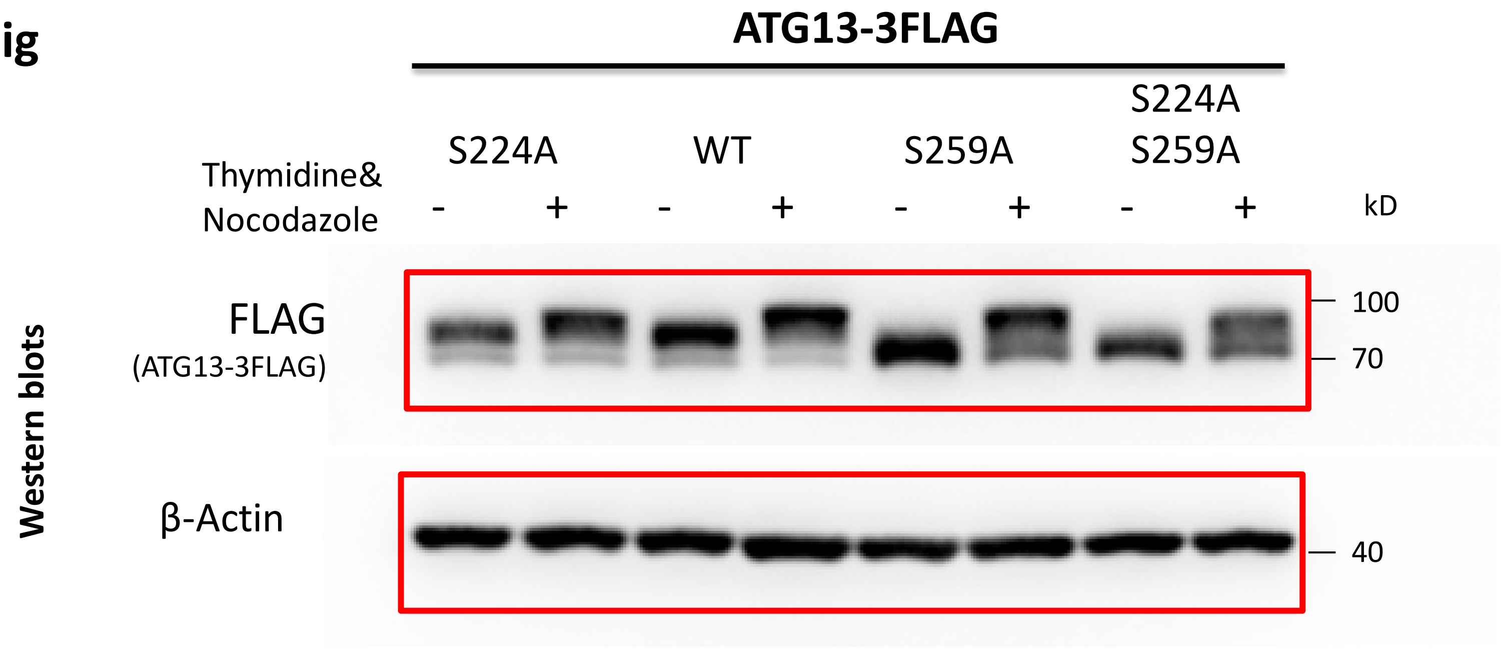

Images were acquired by chemiluminescence CCD camera-based digital imaging instruments (Tanon Fine-do X6).
Approx. molecular weight ladder (L) was indicated.
PVDF Membranes were cut for immunoblotting of more than one protein.

S11A Fig

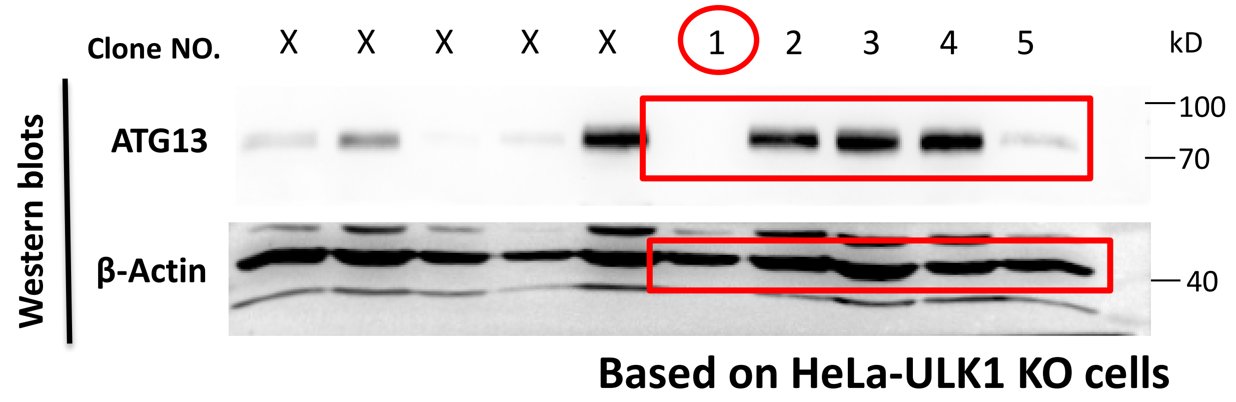

S11B Fig

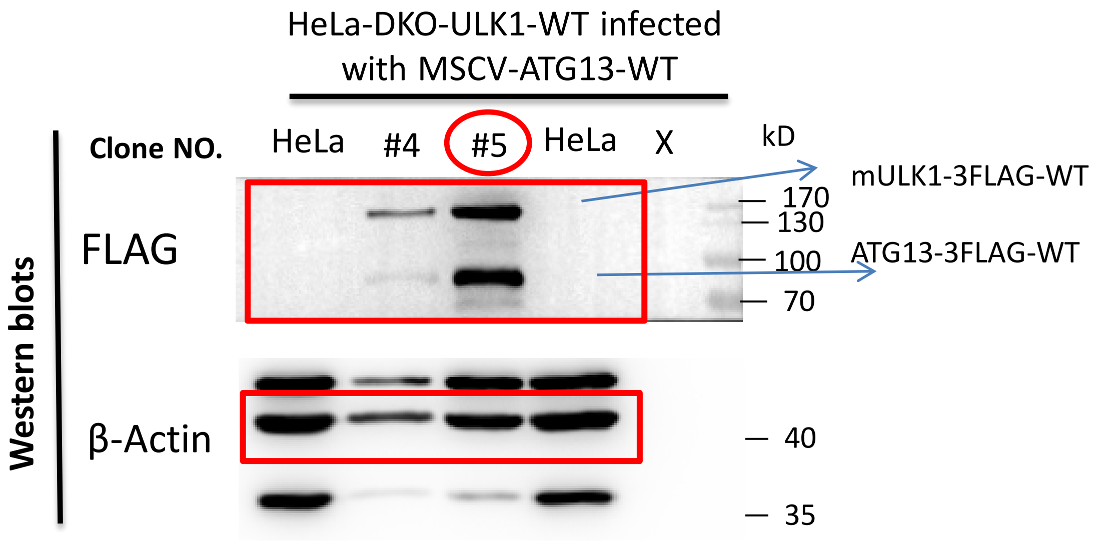

Images were acquired by chemiluminescence CCD camera-based digital imaging instruments (Tanon Fine-do X6).
Approx. molecular weight ladder (L) was indicated.
PVDF Membranes were cut for immunoblotting of more than one protein.

S11C Fig

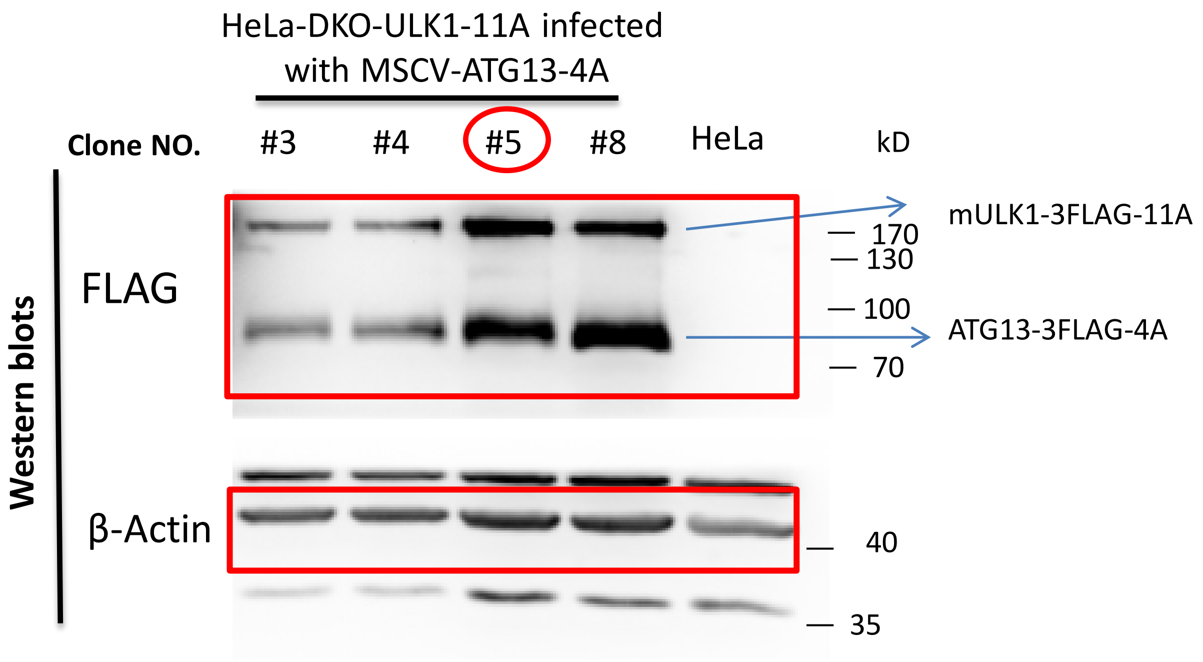

S11D Fig

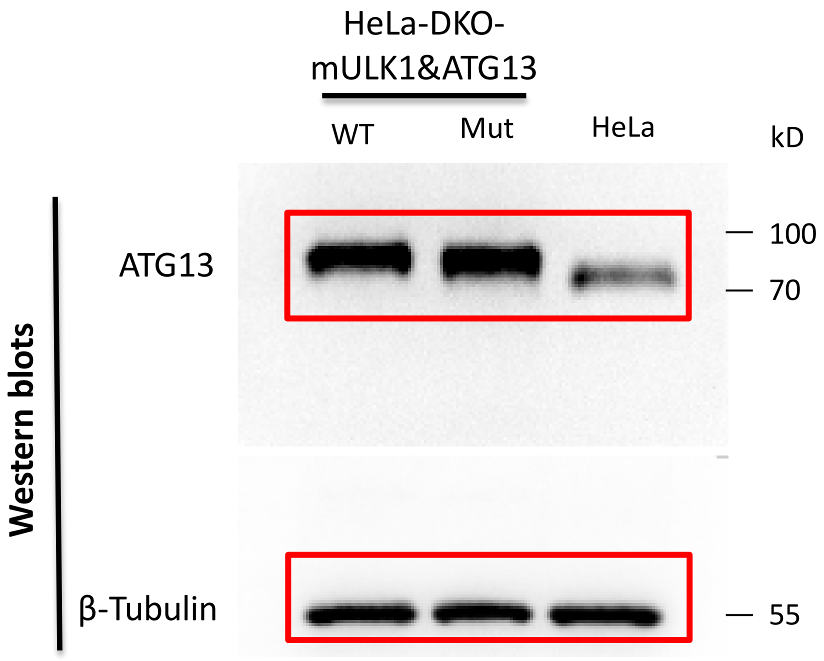

Images were acquired by chemiluminescence CCD camera-based digital imaging instruments (Tanon Fine-do X6).
Approx. molecular weight ladder (L) was indicated.
PVDF Membranes were cut for immunoblotting of more than one protein.

S12A and S12B Fig

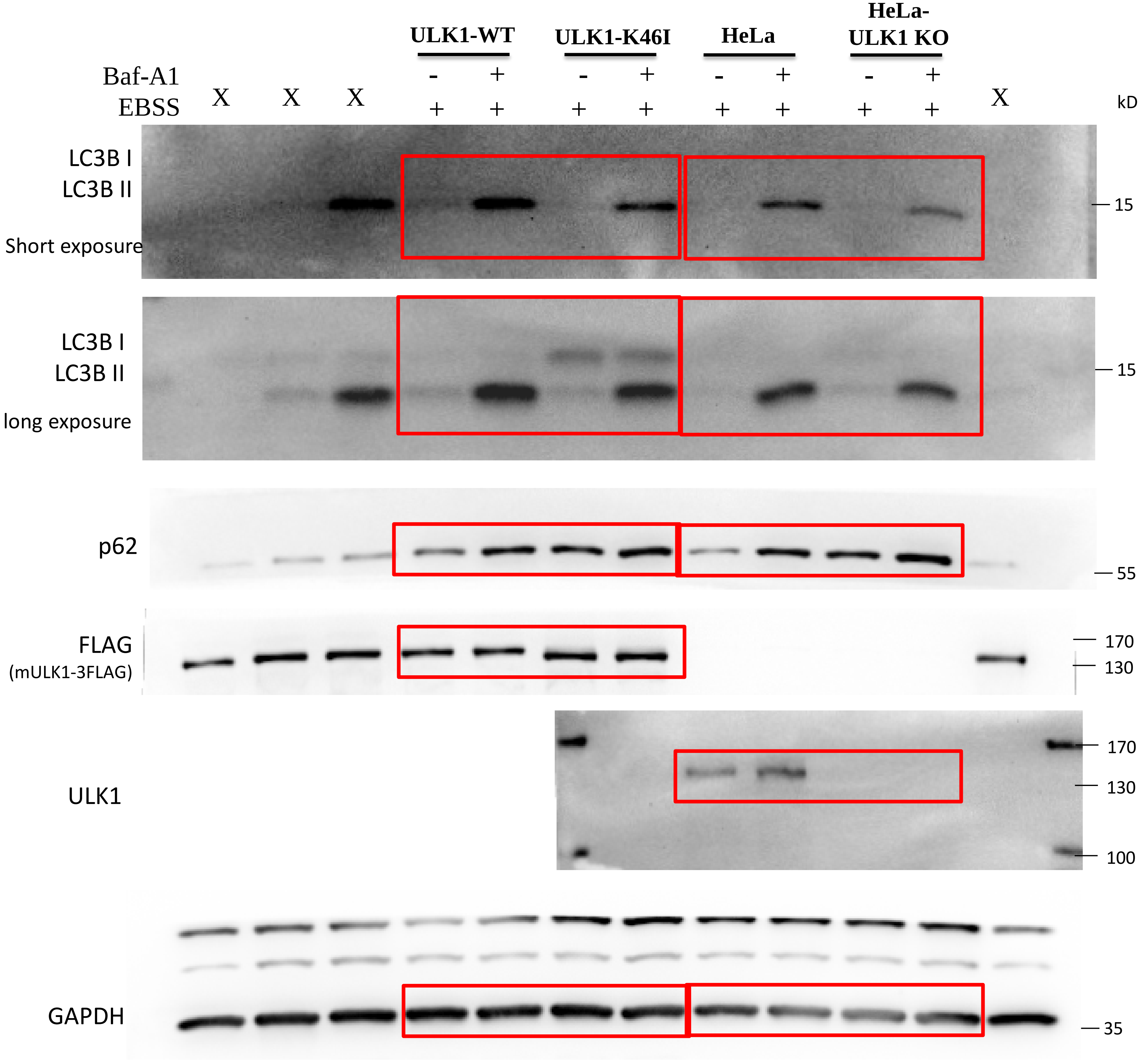

Western blots

Images were acquired by chemiluminescence CCD camera-based digital imaging instruments (Tanon Fine-do X6).
Approx. molecular weight ladder (L) was indicated.
PVDF Membranes were cut for immunoblotting of more than one protein.

S12C Fig

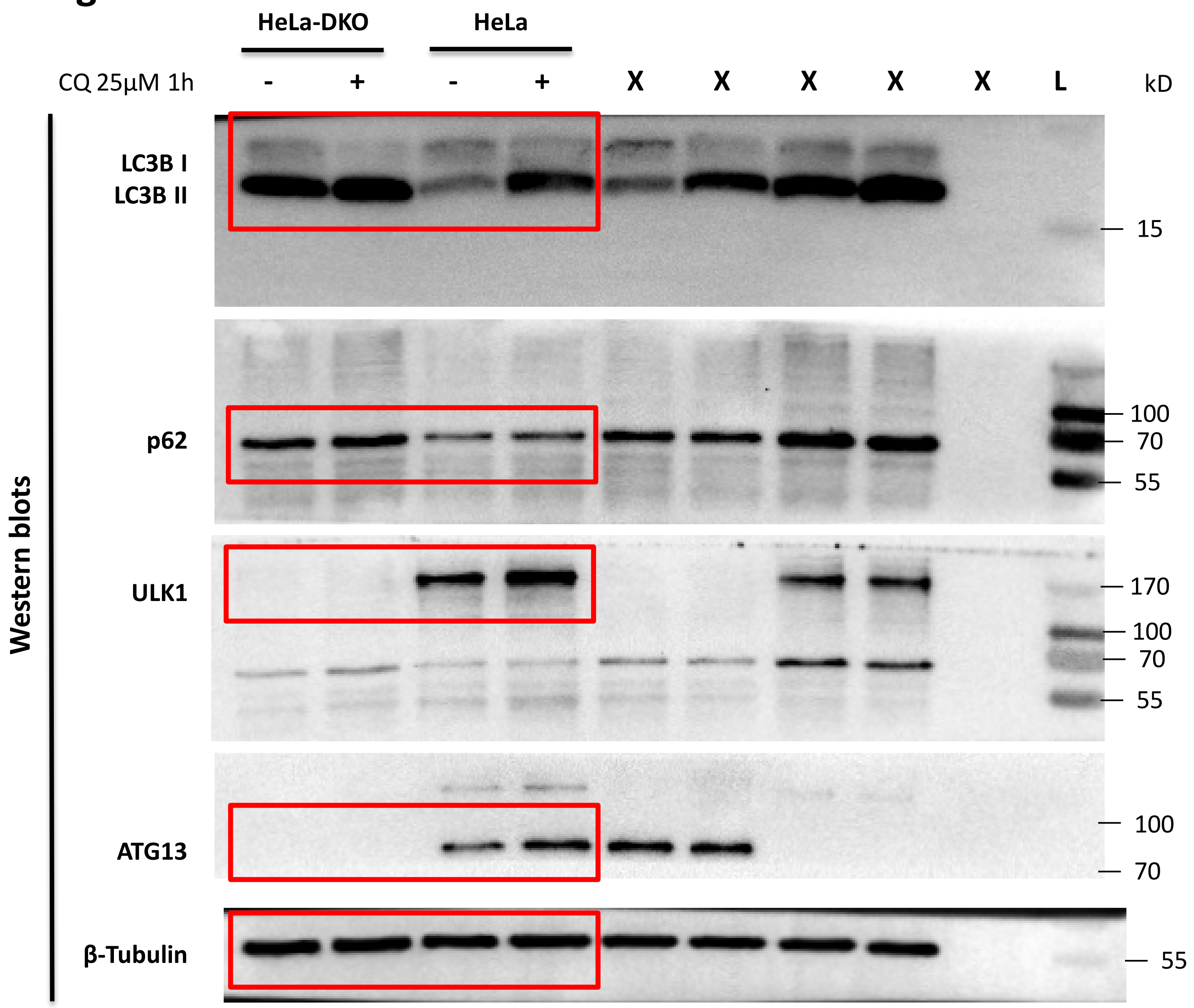

Images were acquired by chemiluminescence CCD camera-based digital imaging instruments (Tanon Fine-do X6).
Approx. molecular weight ladder (L) was indicated.
PVDF Membranes were cut for immunoblotting of more than one protein.

S14B Fig

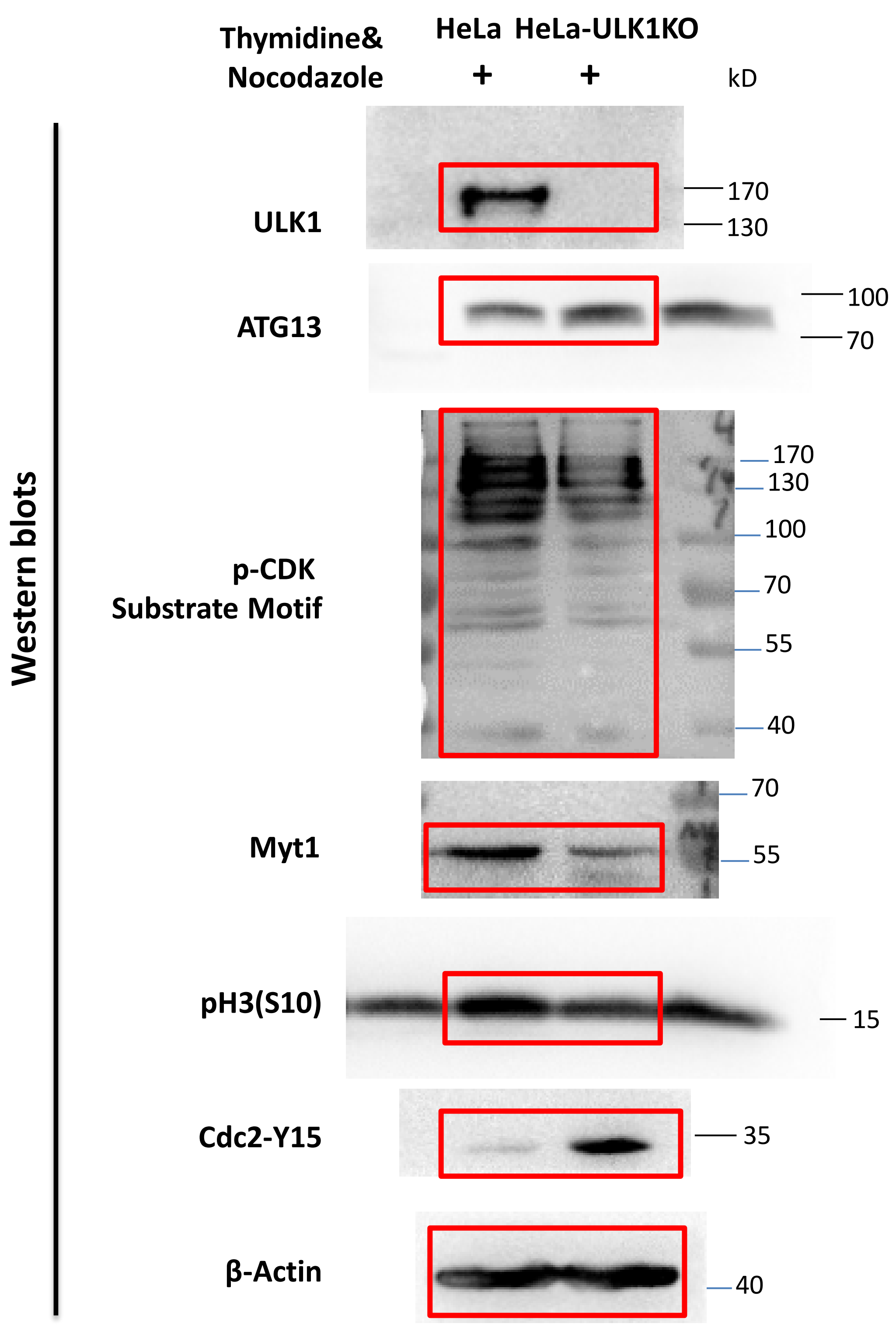

Images were acquired by chemiluminescence CCD camera-based digital imaging instruments (Tanon Fine-do X6).
Approx. molecular weight ladder (L) was indicated.
PVDF Membranes were cut for immunoblotting of more than one protein.

S14D Fig

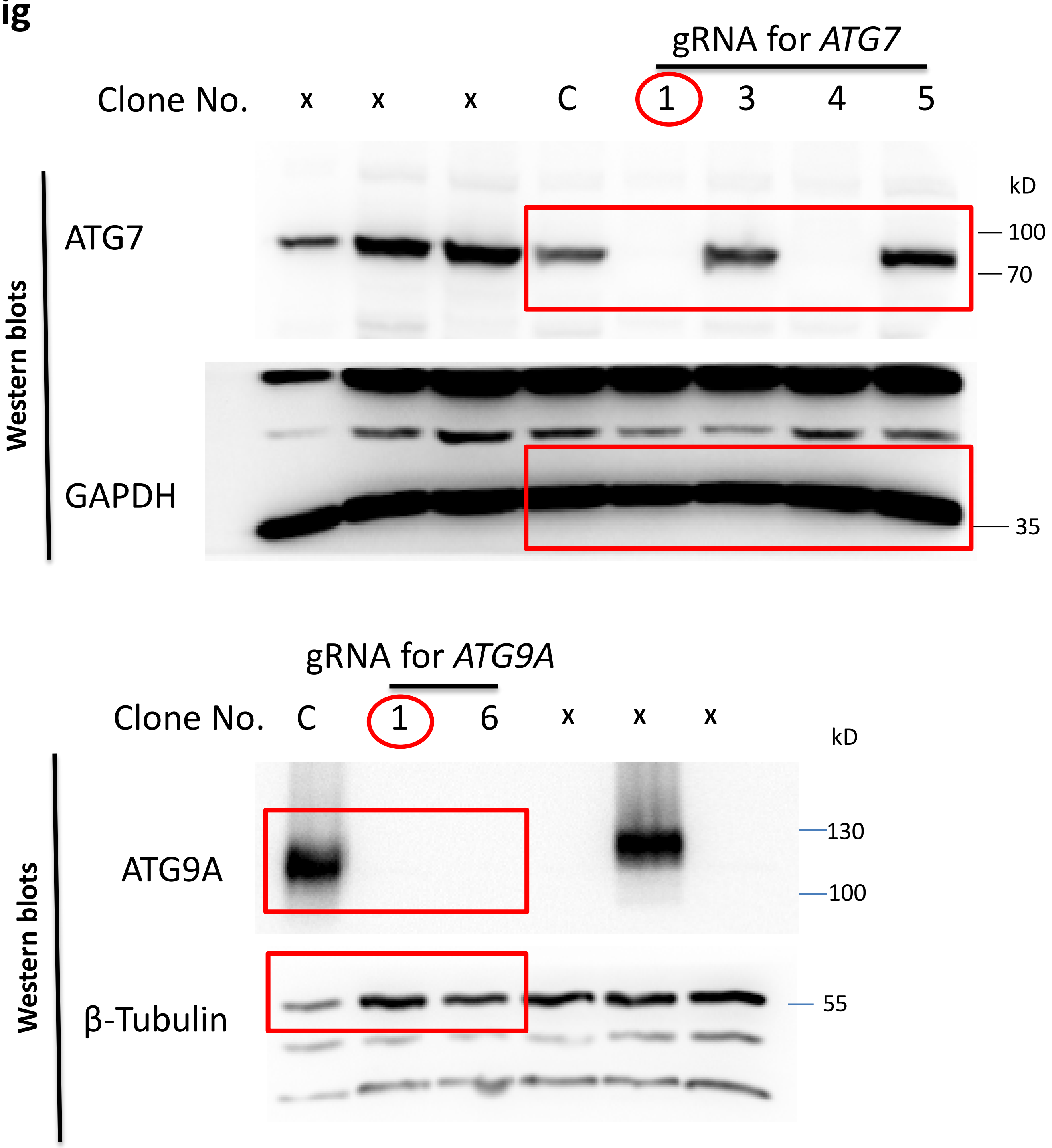

Images were acquired by chemiluminescence CCD camera-based digital imaging instruments (Tanon Fine-do X6).
Approx. molecular weight ladder (L) was indicated.
PVDF Membranes were cut for immunoblotting of more than one protein.

S15B Fig

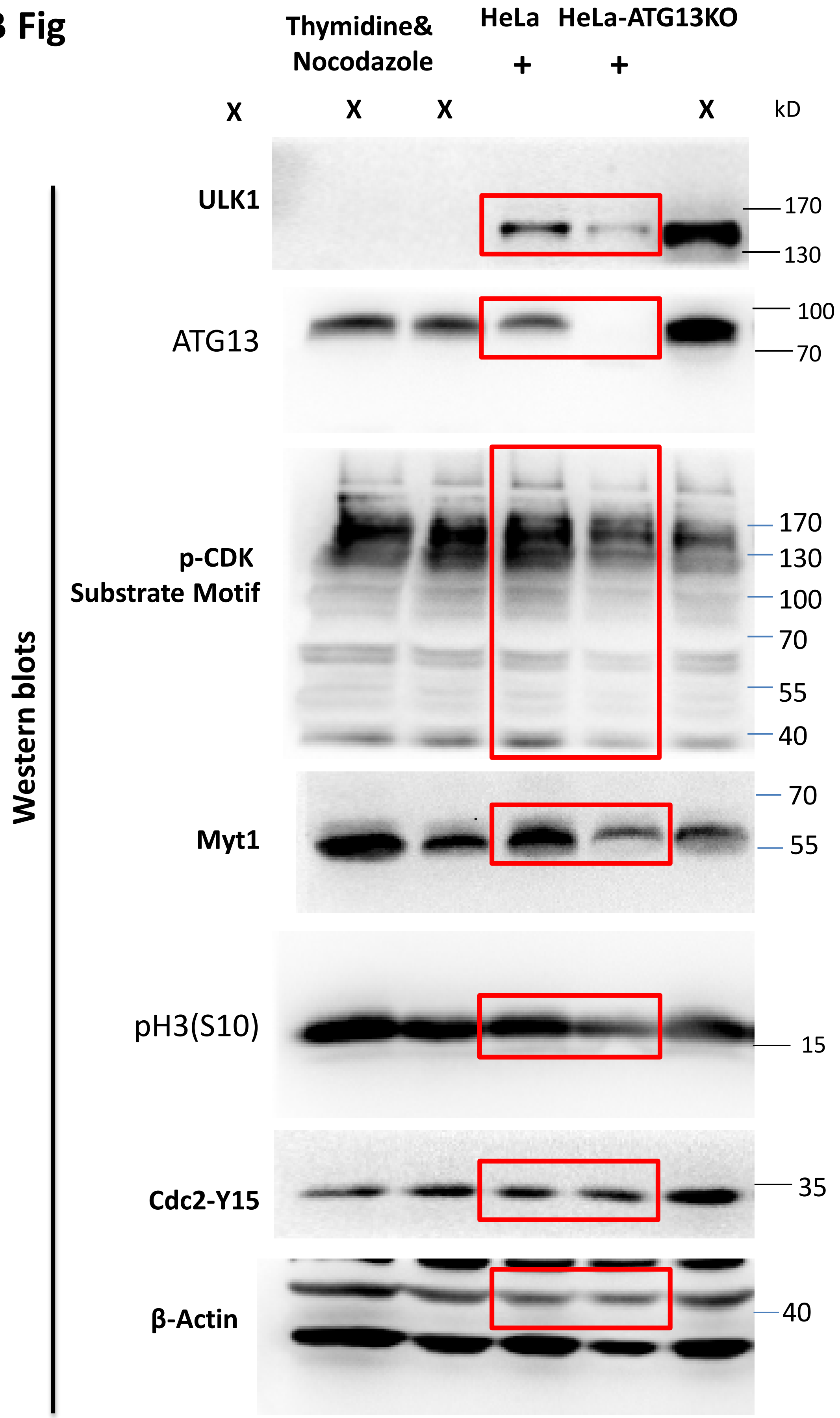

Images were acquired by chemiluminescence CCD camera-based digital imaging instruments (Tanon Fine-do X6).
Approx. molecular weight ladder (L) was indicated.
PVDF Membranes were cut for immunoblotting of more than one protein.

S15D Fig

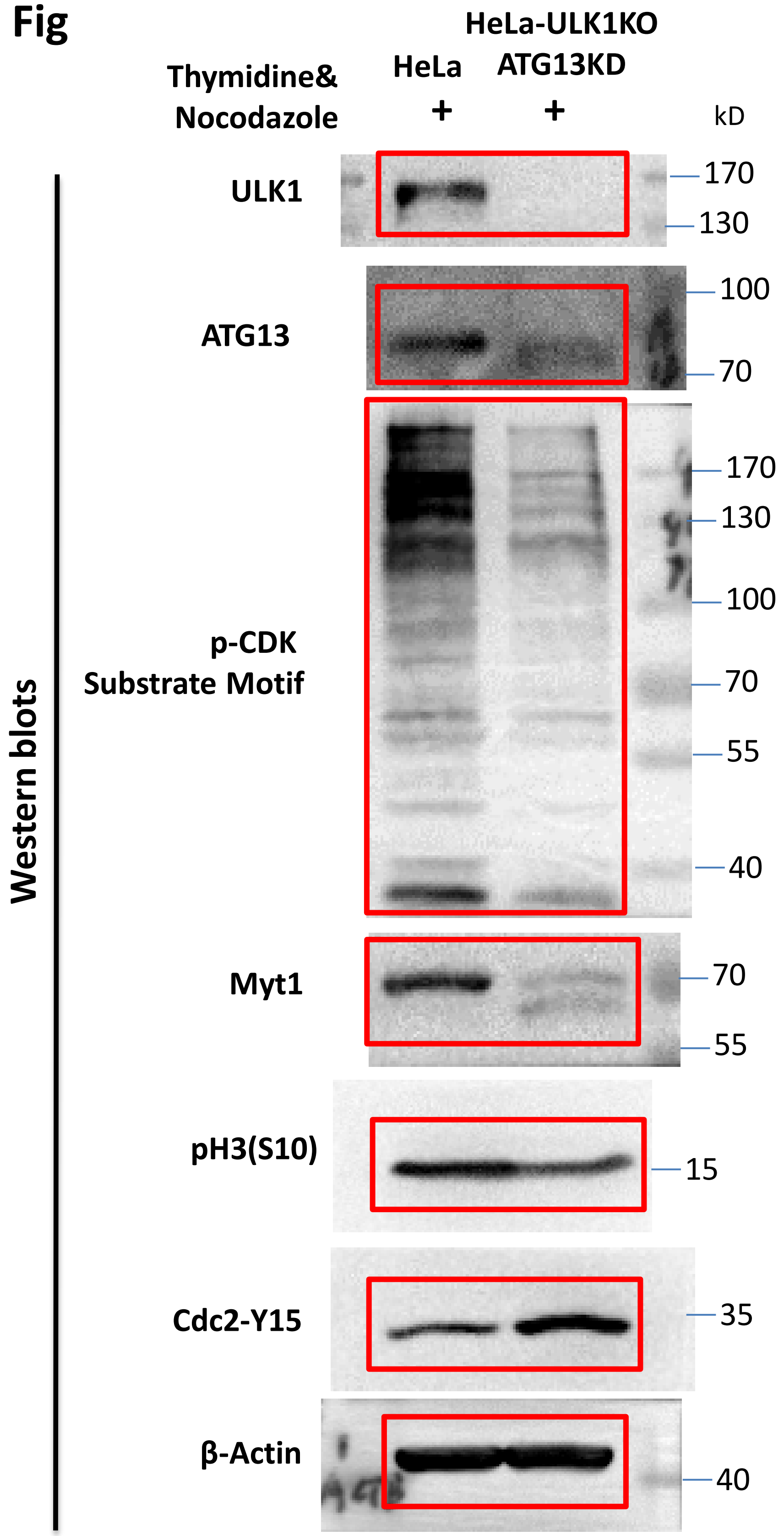

Images were acquired by chemiluminescence CCD camera-based digital imaging instruments (Tanon Fine-do X6).
Approx. molecular weight ladder (L) was indicated.
PVDF Membranes were cut for immunoblotting of more than one protein.
