# Supplementary figures and images for: ULK1-ATG13 and their mitotic phospho-regulation by CDK1 connect autophagy to cell cycle
Source: PLoS Biol. 2020 Jun 9;18(6):e3000288. doi: 10.1371/journal.pbio.3000288 (PMC7282624; doi:10.1371/journal.pbio.3000288)

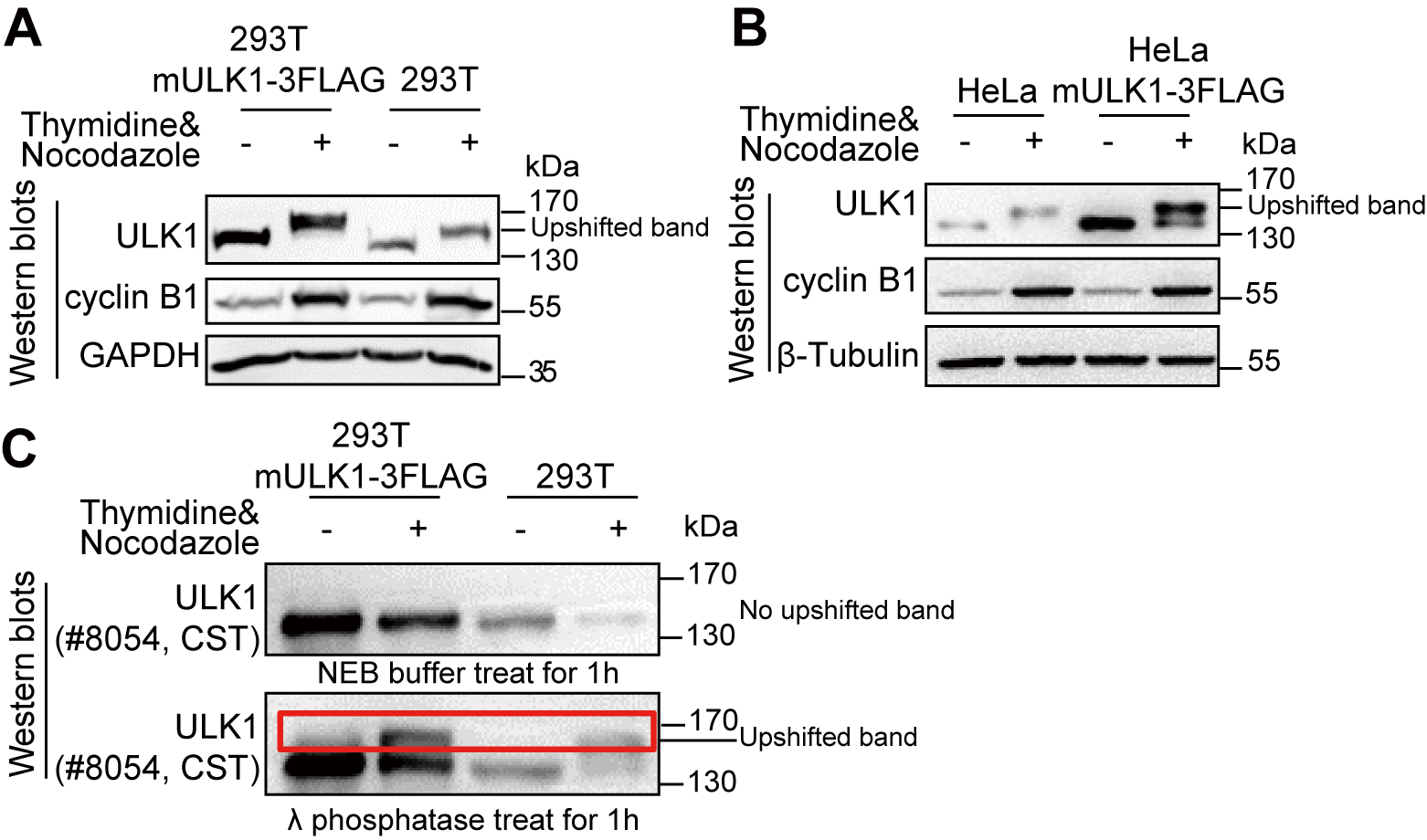

Supplement: S1 Fig — (A-B) Both endogenous human and exogenous mouse ULK1 are upshifted in thymidine and nocodazole-arrested mitosis. 293T and HeLa cells with or without FLAG-tagged mULK1 overexpression were synchronized into mitosis by single-thymidine and nocodazole for western blot analysis. (C) ULK1 phosphorylation in mitosis interferes with ULK1 antibody recognition. The ULK1 antibody (Cell Signaling Technology, #8054) could not recognize the upshifted band for mitotic ULK1 but could recognize when the PVDF membrane was treated with lambda phosphatase for 1 hour. PVDF, polyvinylidene fluoride; ULK1, unc-51-like autophagy activating kinase 1. (TIF) [file pbio.3000288.s003.tif]

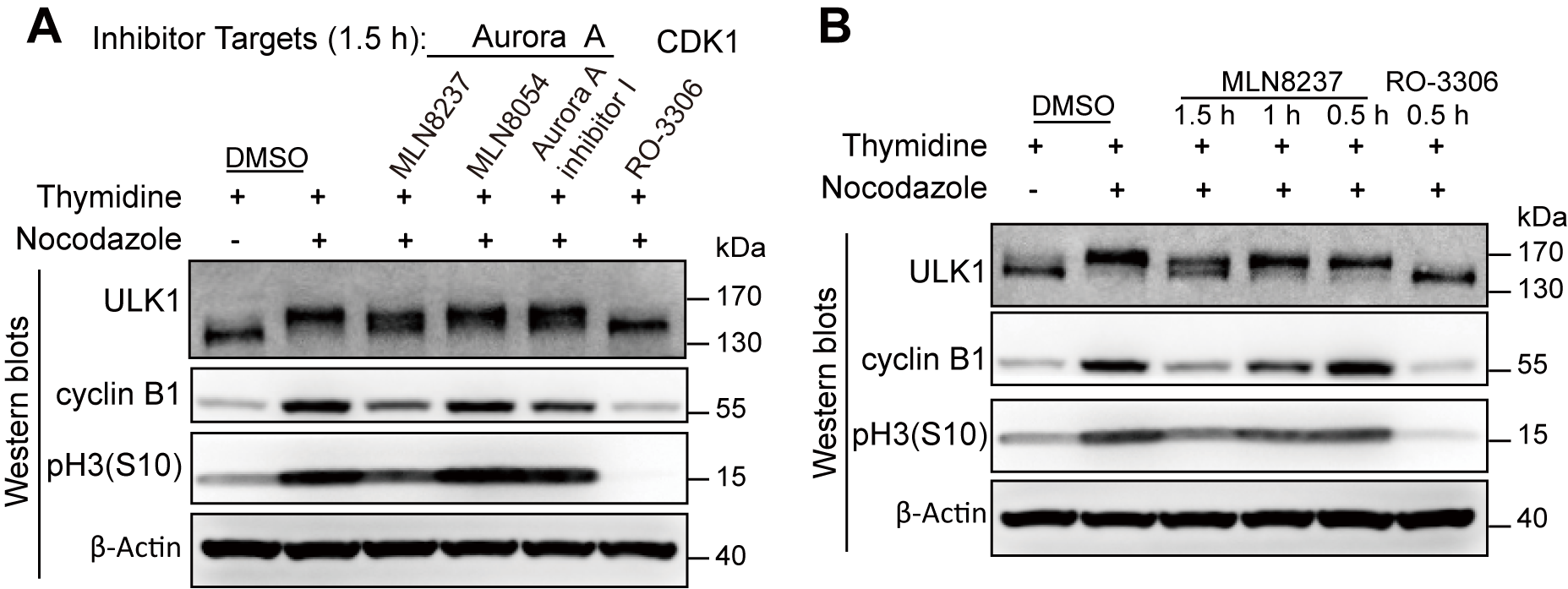

Supplement: S2 Fig — (A-B) HeLa cells synchronized and treated as Fig 3A were subjected to western blot analysis. However, we found that the other 2 Aurora A inhibitors, MLN8054 and Aurora A inhibitor I, did not affect ULK1 band shift (A). In addition, MLN8237 treatment for a shorter time (1 hour or 0.5 hours) did not cause ULK1 band shift change as 1.5-hour treatment (B). MLN8237, MLN8054, and Aurora A inhibitor I were Aurora A inhibitor. CDK, cyclin-dependent kinase; ULK1, unc-51-like autophagy activating kinase 1. (TIF) [file pbio.3000288.s004.tif]

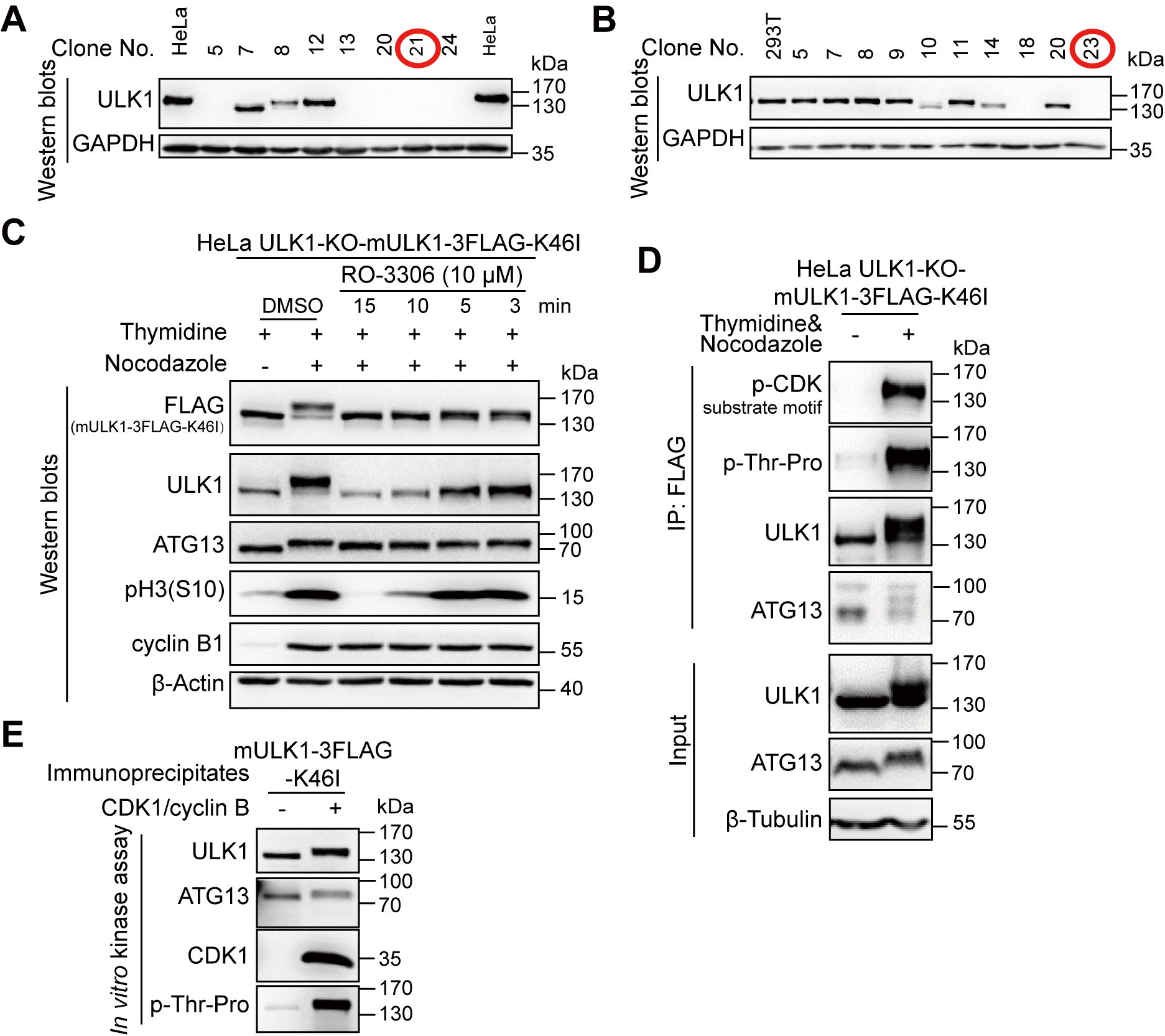

Supplement: S3 Fig — (A-B) ULK1-KO cells were established. HeLa and 293T cells transiently transfected with the CRISPR/Cas9 plasmid subcloned gRNA for human ULK1 were screened by western blot analysis and the ULK1-KO clones were identified. The red circles indicate the ULK1-KO clones for the following assay. (C-D) K46I kinase-dead ULK1 also underwent significant electrophoretic mobility shift and phosphorylation in mitosis as WT ULK1. HeLa ULK1-KO cells reconstituted with FLAG-tagged mULK1-K46I were treated as Figs 1C and 3B (the lower panel) and then analyzed by western blot analysis and immunoprecipitation, respectively. (E) In vitro kinase assay indicated that purified CDK1/cyclin B could induce K46I kinase-dead ULK1 to undergo significant electrophoretic mobility shift and phosphorylation. CDK, cyclin-dependent kinase; gRNA, guide RNA; KO, knockout; mULK1, mouse ULK1; ULK1, unc-51-like autophagy activating kinase 1; WT, wild type. (TIF) [file pbio.3000288.s005.tif]

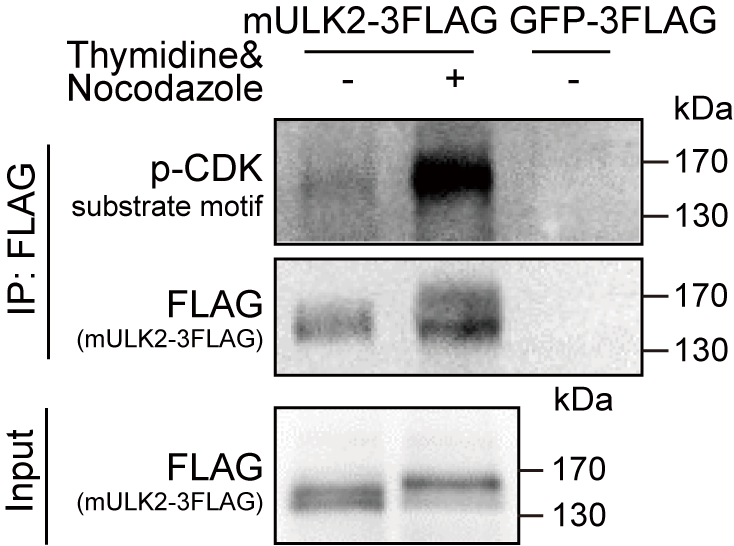

Supplement: S4 Fig — The 293T cells transiently transfected with FLAG-tagged mULK2 treated as Fig 3A were analyzed by western blot analysis and immunoprecipitation. mULK2, mouse ULK2; ULK2, unc-51-like autophagy activating kinase 2. (TIF) [file pbio.3000288.s006.tif]

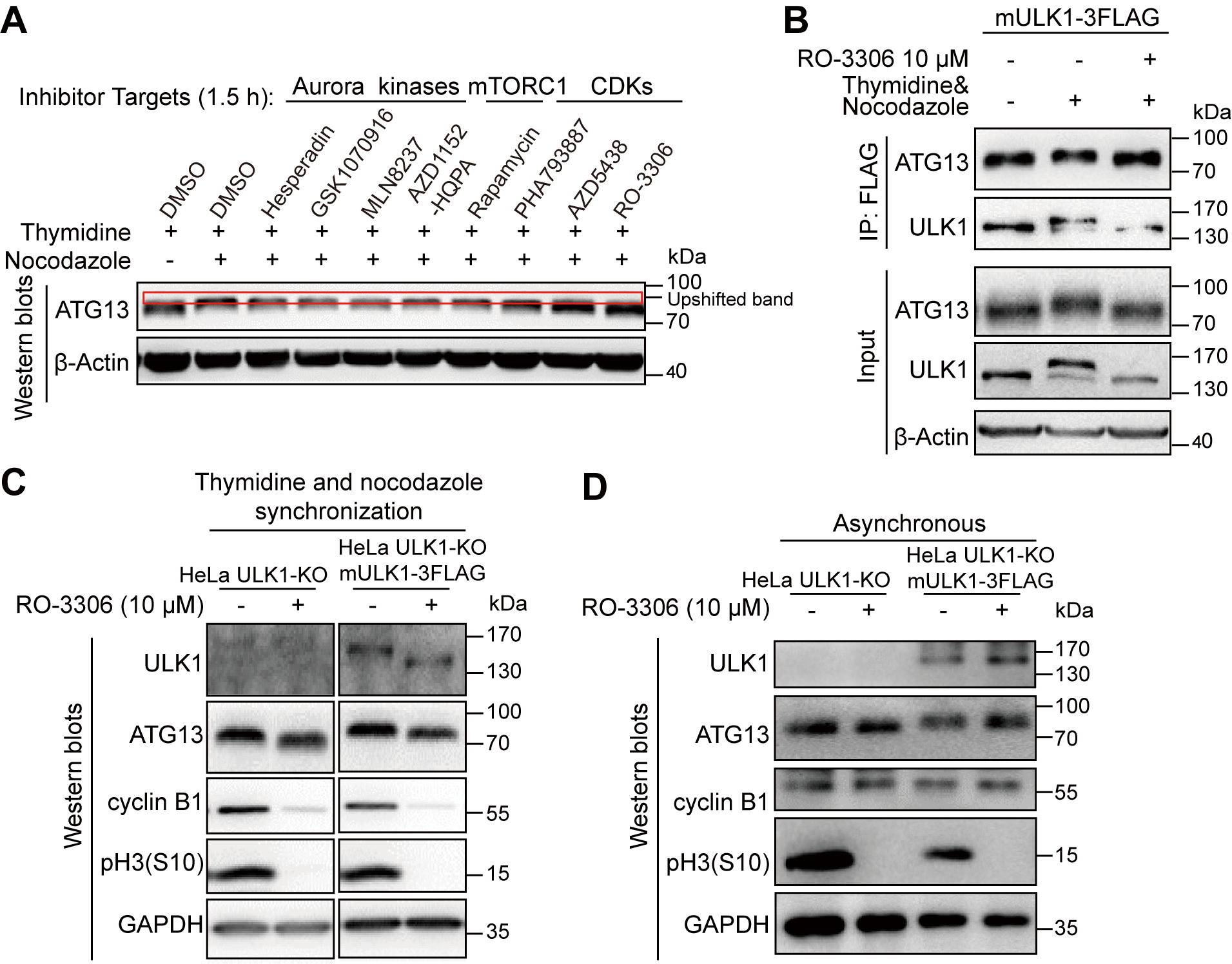

Supplement: S5 Fig — (A) HeLa cells were treated as Fig 4C for ATG13 mobility shift analysis. (B) ATG13 mobility shift in mitosis is decreased by CDK1 inhibitor RO-3306, similarly to ULK1, although to a lesser extent. The 293T cells overexpressing FLAG-tagged mULK1 were synchronized by single-thymidine in the presence or absence of nocodazole, treated with 10 μM RO-3306 for 5 or 30 minutes. The coimmunoprecipitate by FLAG antibody was subjected to immunoblotting with ATG13, FIP200 antibodies. (C-D) ULK1 expression level does not affect ATG13 mobility shift in mitosis. HeLa ULK1-KO cells with or without FLAG-tagged mULK1 expression synchronized into mitosis with thymidine and nocodazole (C) or in asynchronous condition (D) were treated with 10 μM RO-3306 for 30 minutes for western blot analysis. ATG, autophagy-related; CDK, cyclin-dependent kinase; FIP200, FAK family-interacting protein of 200 kDa; KO, knockout; mULK1, mouse ULK1; ULK1, unc-51-like autophagy activating kinase 1. (TIF) [file pbio.3000288.s007.tif]

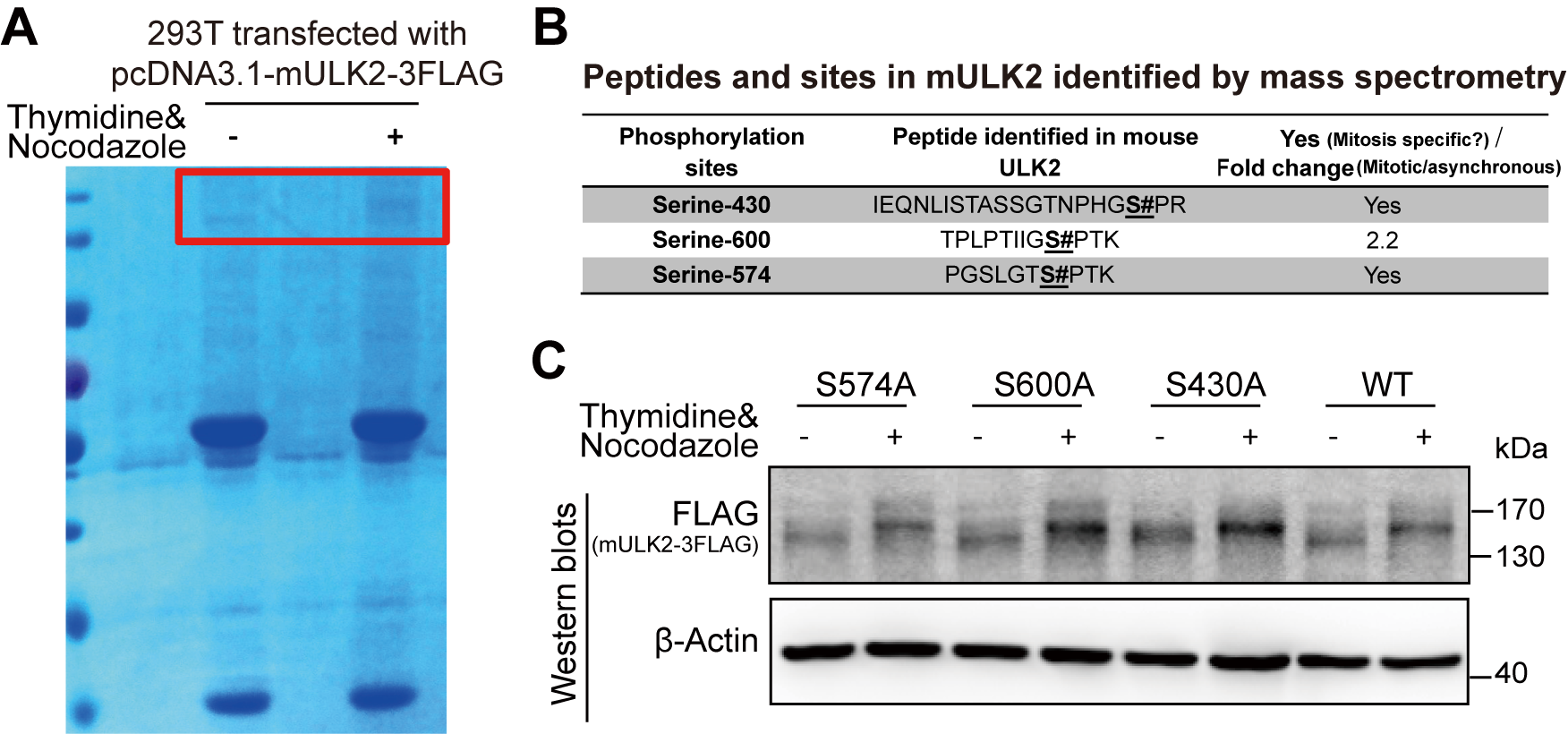

Supplement: S6 Fig — (A-B) The preliminary identification of ULK2 phosphorylation sites in mitosis. The immunoprecipitate with FLAG antibody in asynchronous or mitotic 293T cells transfected with mULK2-3FLAG was subjected to SDS-PAGE and Coomassie brilliant blue staining (A) and mass spectrometry analysis of phosphorylation sites for mitotic mULK2 compared with asynchronous mULK2 (B). (C) The contribution of the potential residues to mitotic ULK2 band shift. HeLa cells were transfected with the mutant mULK2-3FLAG plasmid in indicated sites and analyzed by cell cycle synchronization and western blot. mULK2, mouse ULK2; ULK2, unc-51-like autophagy activating kinase 2. (TIF) [file pbio.3000288.s008.tif]

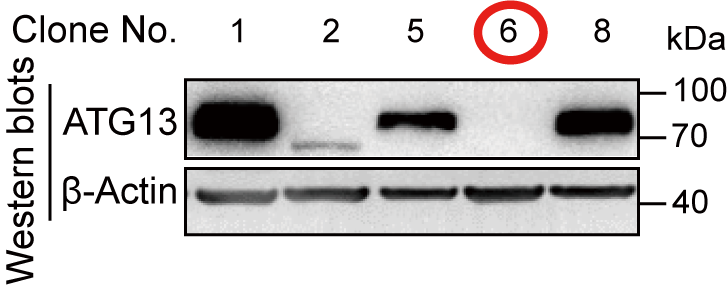

Supplement: S7 Fig — HeLa cells were transiently transfected with the CRISPR/Cas9 plasmid that subcloned gRNA for human ATG13. The ATG13-KO clones were screened by western blot and identified. The red circle indicates the ATG13-KO clones for the following assay. ATG, autophagy-related; gRNA, guide RNA; KO, knockout. (TIF) [file pbio.3000288.s009.tif]

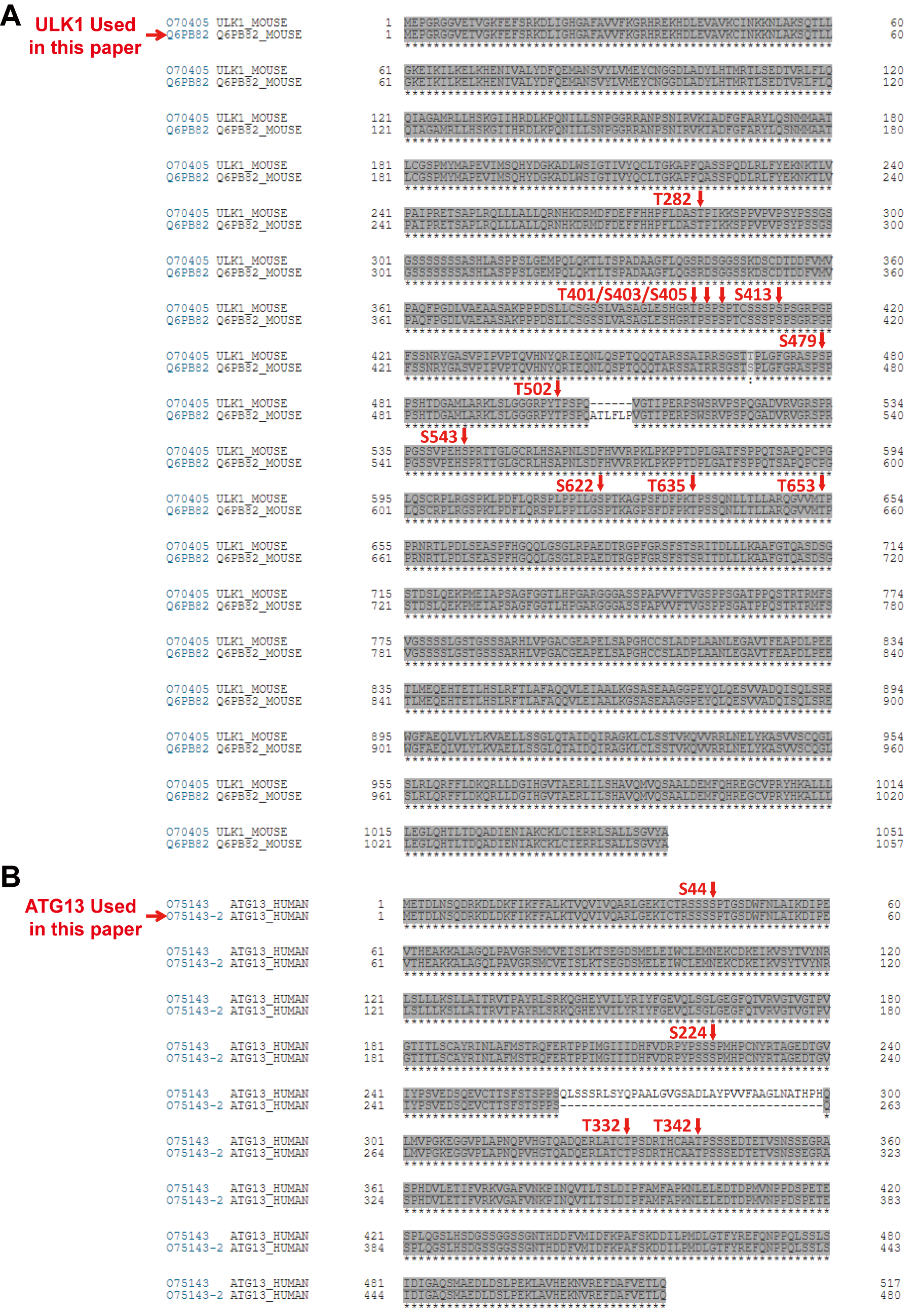

Supplement: S8 Fig — (A) The difference between the construct we used in this paper (Q6PB82 in Uniprot, BC059835 in GenBank) with the ULK1 used in some other studies (O70405 in Uniprot) is that there are 6 additional amino acids at position 507–512 (ATLFLP) of Q6PB82 and a conversion from S to T at position 469 of Q6PB82. (B) The human ATG13 cloned from the cDNA of HeLa cells, isofom2 (O75143-2 in Uniprot, BC002378 in GenBank), is used in this paper. It differs from O75143 (isoform 1) used in other studies with the missing region in the amino acids at position 263–299. The phosphorylation sites we identified in both ULK1 and ATG13 are conserved between different isoforms (A-B), which were aligned in Uniprot. ATG, autophagy-related; ULK1, unc-51-like autophagy activating kinase 1. (TIF) [file pbio.3000288.s010.tif]

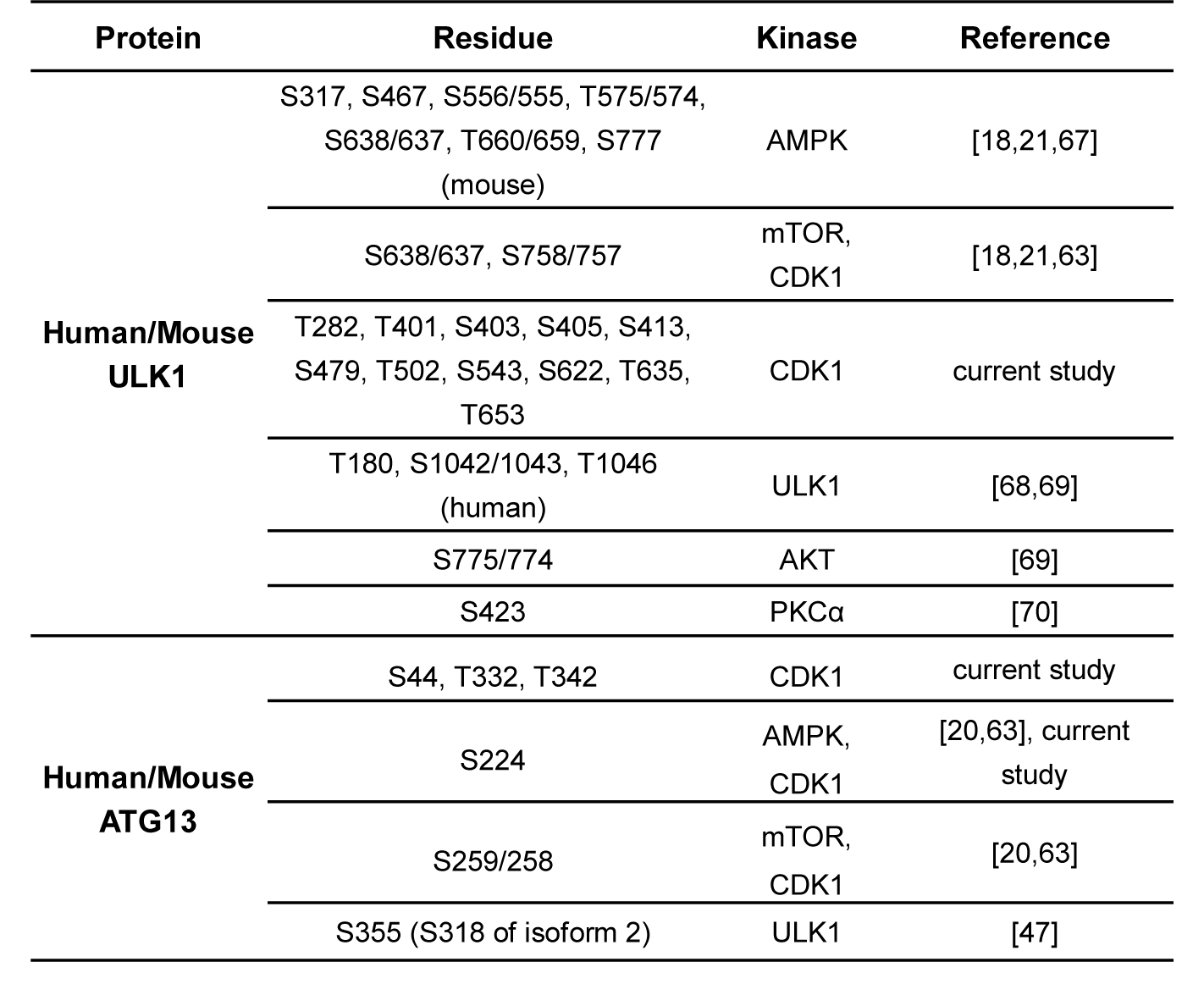

Supplement: S9 Fig — The identified phosphorylated residues of the mouse ULK1 and human ATG13 from “current study” and literature [18,20,21,47,63,67–70] are summarized. ATG, autophagy-related; ULK1, unc-51-like autophagy activating kinase 1. (TIF) [file pbio.3000288.s011.tif]

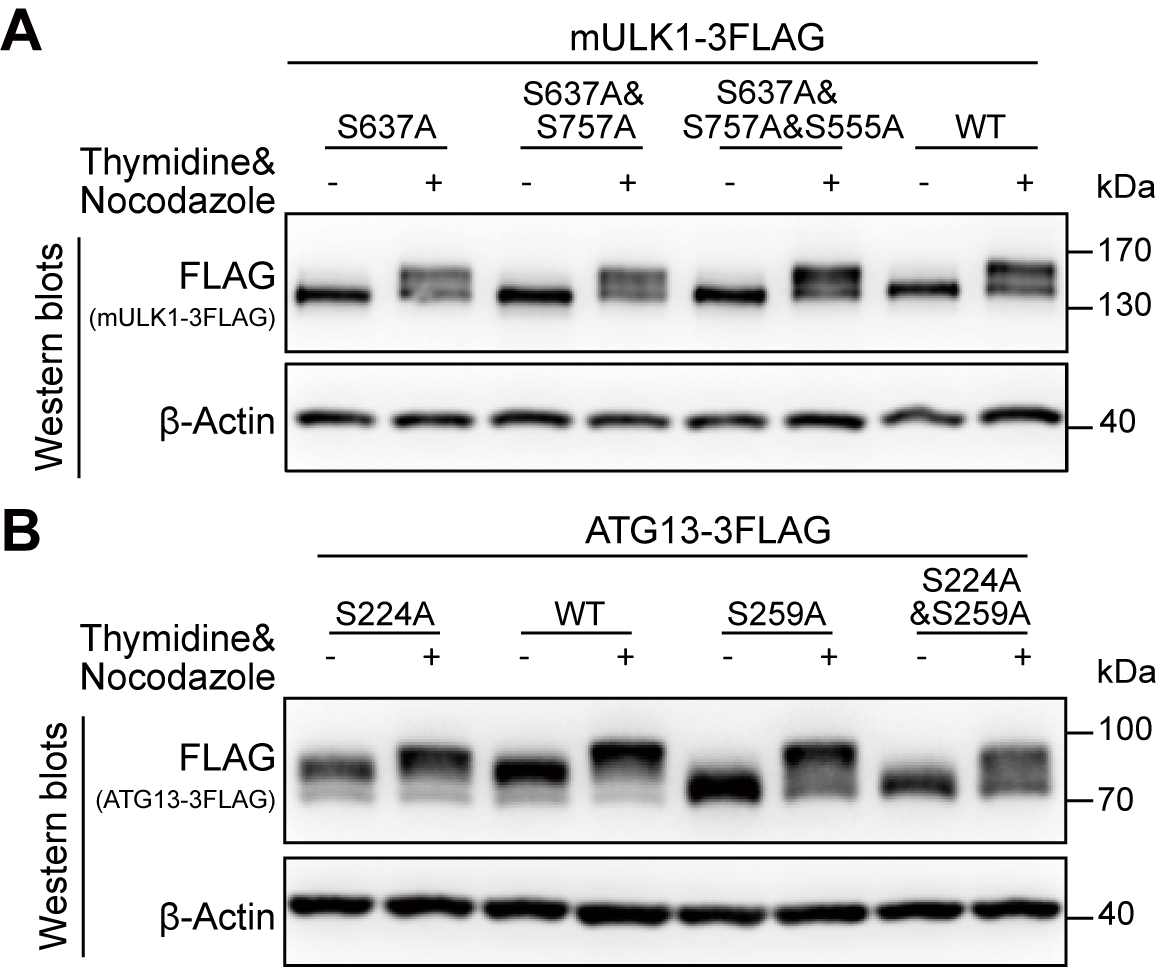

Supplement: S10 Fig — (A-B) The alanine mutants of ULK1 and ATG13 at fundamental mTOR/AMPK phosphorylation sites were constructed in ATG13-KO or ULK1-KO cells and examined their contribution to mitotic ULK1 (A) or ATG13 (B) band shift in mitosis. AMPK, AMP-activated protein kinase; KO, knockout; mTOR, mammalian target-of-rapamycin; ULK1, unc-51-like autophagy activating kinase 1. (TIF) [file pbio.3000288.s012.tif]

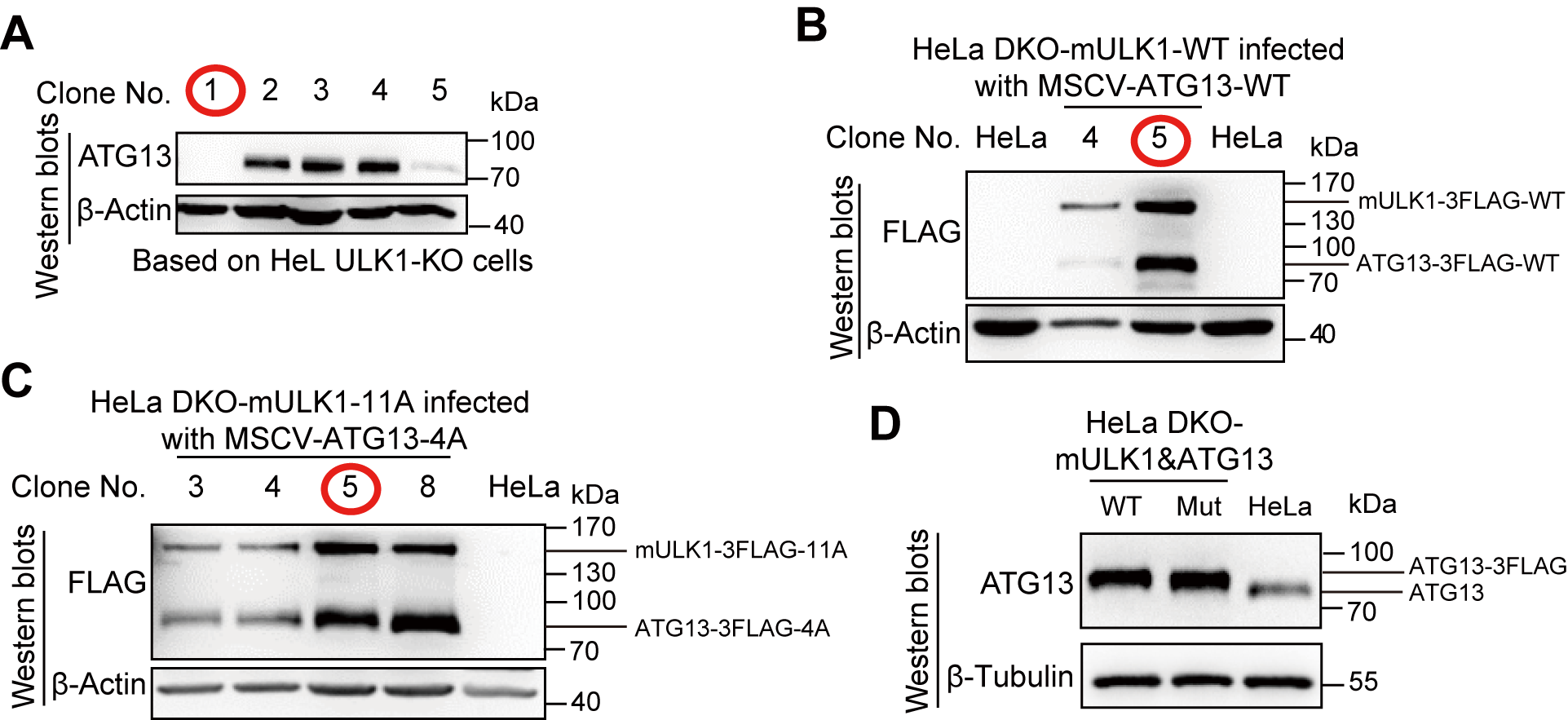

Supplement: S11 Fig — (A) ULK1 and ATG13 DKO cells establishment. HeLa ULK1-KO cells were transiently transfected with the CRISPR/Cas9 plasmid that subcloned gRNA for human ATG13. The ATG13-KO clones were screened by western blot analysis and identified as ULK1 and ATG13 DKO cells. The red circles indicate the ULK1 and ATG13 DKO clones for the following assays. (B-C) The ULK1 and ATG13 double WT or mutant cell lines were established by ATG13-WT/4A MSCV infection based on HeLa-DKO cell reconstituted with FLAG-tagged WT or 11A mutant mULK1. (D) The expression of ATG13 in ULK1 and ATG13 double WT or mutant cell lines and HeLa cells. 11A, S622&T635&T653&S479&S543&S413&T401&S403&S405&T282&T502A; ATG, autophagy-related; DKO, double knockout; gRNA, guide RNA; KO, knockout; MSCV, murine stem cell virus; mULK1, mouse ULK1; ULK1, unc-51-like autophagy activating kinase 1; WT, wild type. (TIF) [file pbio.3000288.s013.tif]

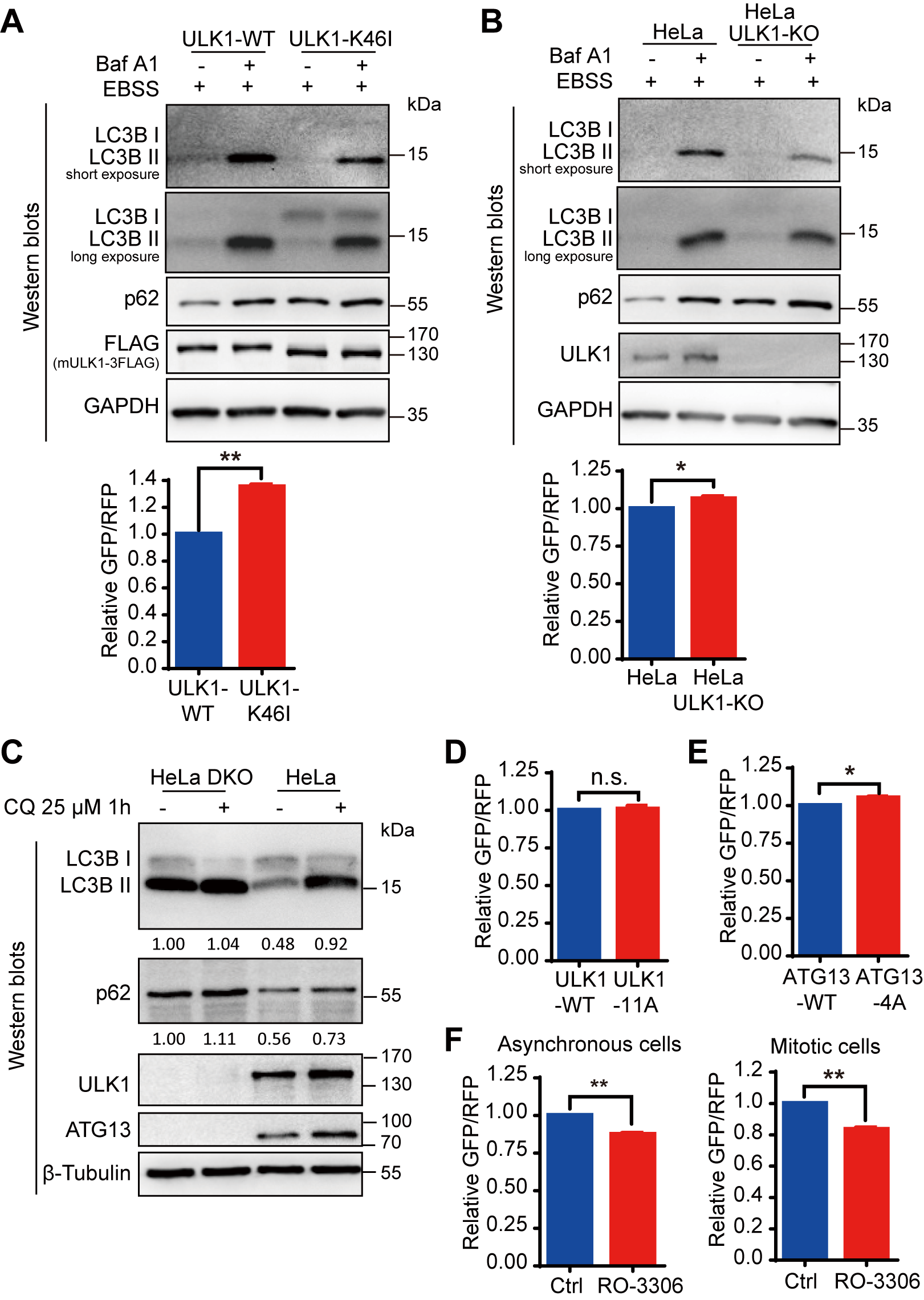

Supplement: S12 Fig — (A-B) The autophagy inhibition was compared between FLAG-tagged wild-type and K46I kinase dead mULK1, HeLa and HeLa ULK1-KO, respectively. Cells were treated with EBSS starvation for 1.5 hours with or without Baf A1 and examined by western blot. GFP-LC3-RFP was stably expressed in indicated cell lines. The autophagy activity was detected by flow cytometry for GFP and RFP fluorescence intensity. (C) The autophagic activity of HeLa and HeLa-DKO cells in mitosis. Cells were synchronized into mitosis by thymidine release and nocodazole arrest. Mitotic cells were collected by shake-off and treated by 25 μM CQ for 1 hour. (D-E) The mitotic autophagy activity in ULK1-11A mutant or ATG13-4A mutant cells was determined by relative GFP/RFP ratio. Cells were treated as Fig 6C. (F) The effect of CDK1 inhibition by RO-3306 on autophagy. HeLa cells stably expressing GFP-LC3-RFP were treated by 10 μM RO-3306 for 4 hours in asynchronous condition and 20 minutes in mitotic condition synchronized by thymidine release and nocodazole arrest. Cells in (D, E, and F) were collected by flow cytometry for the relative GFP/RFP ratio. n = 3, *p < 0.05, **p < 0.01. Numerical data underlying the figure panels are available in S1 Data. 11A, S622&T635&T653&S479&S543&S413&T401&S403&S405&T282&T502A; ATG, autophagy-related; Baf A1, bafilomycin A1; CDK, cyclin-dependent kinase; CQ, chloroquine; DKO, double knockout; EBSS, Earle’s balanced salt solution; GFP, green fluorescent protein; mULK1, mouse ULK1; n.s., not significant; RFP, red fluorescent protein; ULK1, unc-51-like autophagy activating kinase 1. (TIF) [file pbio.3000288.s014.tif]

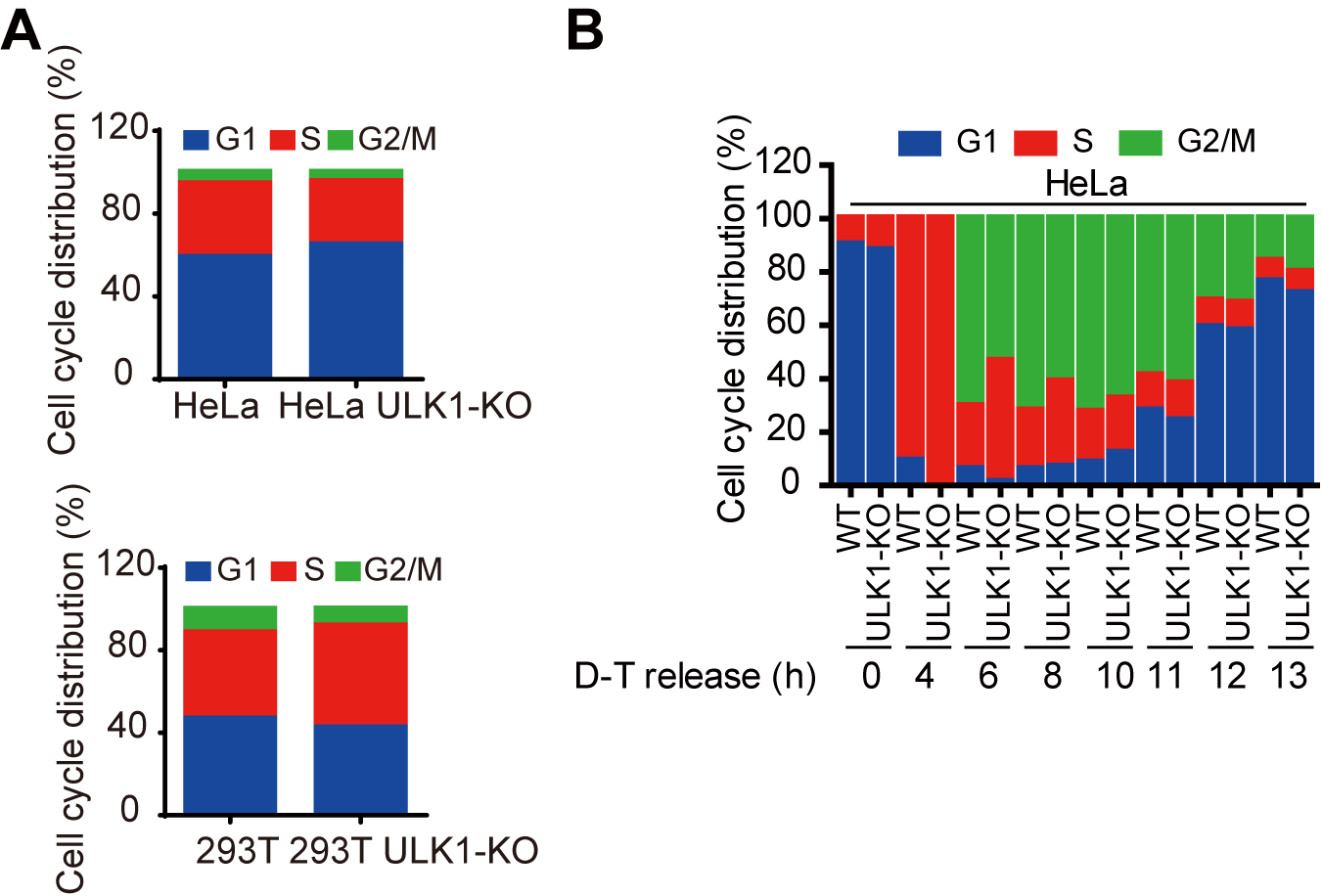

Supplement: S13 Fig — (A) ULK1-KO does not affect cell cycle distribution in HeLa and 293T cells. Cell cycle distribution was analyzed by flow cytometry in asynchronous WT and ULK1-KO HeLa or 293T cells. (B) ULK1-KO slightly delays S/G2 transition. HeLa WT or ULK1-KO cells synchronized with double-thymidine and nocodazole were subjected to cell cycle analysis by flow cytometry. Numerical data underlying the figure panels are available in S1 Data. KO, knockout; ULK1, unc-51-like autophagy activating kinase 1; WT, wild type. (TIF) [file pbio.3000288.s015.tif]

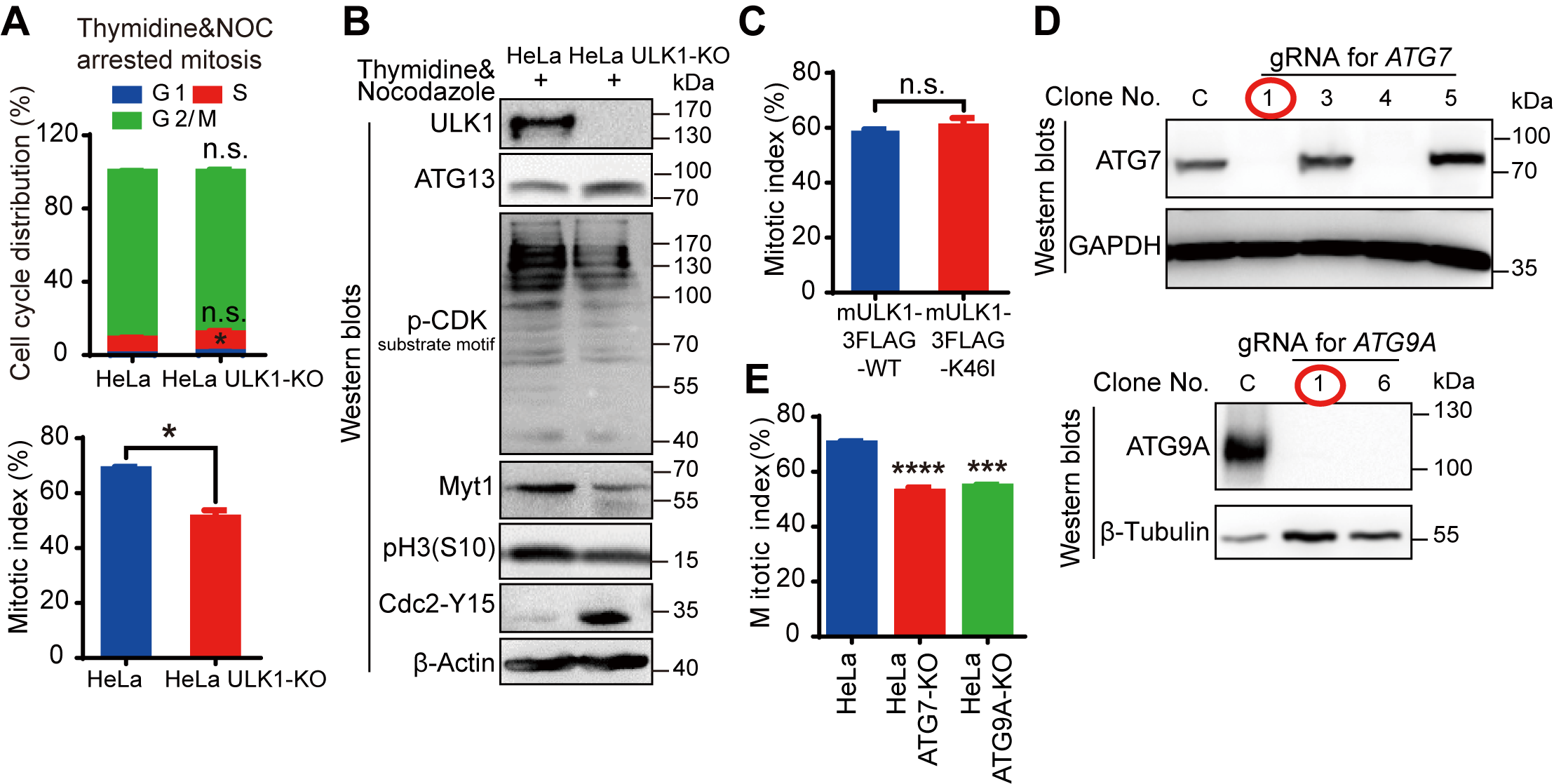

Supplement: S14 Fig — (A-B) Mitotic index is decreased in ULK1-KO cells synchronized by single-thymidine and nocodazole. HeLa WT or ULK1-KO cells synchronized into mitosis released from thymidine for 5 hours and nocodazole for another 7 hours were subjected to either PI and pH3(S10) co-staining for cell cycle and mitotic index analysis by flow cytometry (A) or western blot analysis for cell cycle markers (B). (C) Mitotic progression was not affected by K46I kinase-dead ULK1. HeLa ULK1-KO cells reconstituted with FLAG-tagged WT or K46I kinase-dead mULK1 were synchronized into mitosis and subjected to pH3(S10) staining for mitotic index analysis by flow cytometry. (D-E) The effect of ATG7/ATG9A-KO on mitotic entry. HeLa cells with ATG7 or ATG9A-KO were established by CRISPR/Cas9 (D) and treated as above for the detection of mitotic index analyzed by 1-way ANOVA followed by Tukey’s multiple comparison test (E). n = 3, *p < 0.05, ***p < 0.001, ****p < 0.0001. Numerical data underlying the figure panels are available in S1 Data. ATG, autophagy-related; KO, knockout; mULK1, mouse ULK1; n.s., not significant; PI, propidium iodide; ULK1, unc-51-like autophagy activating kinase 1; WT, wild type. (TIF) [file pbio.3000288.s016.tif]

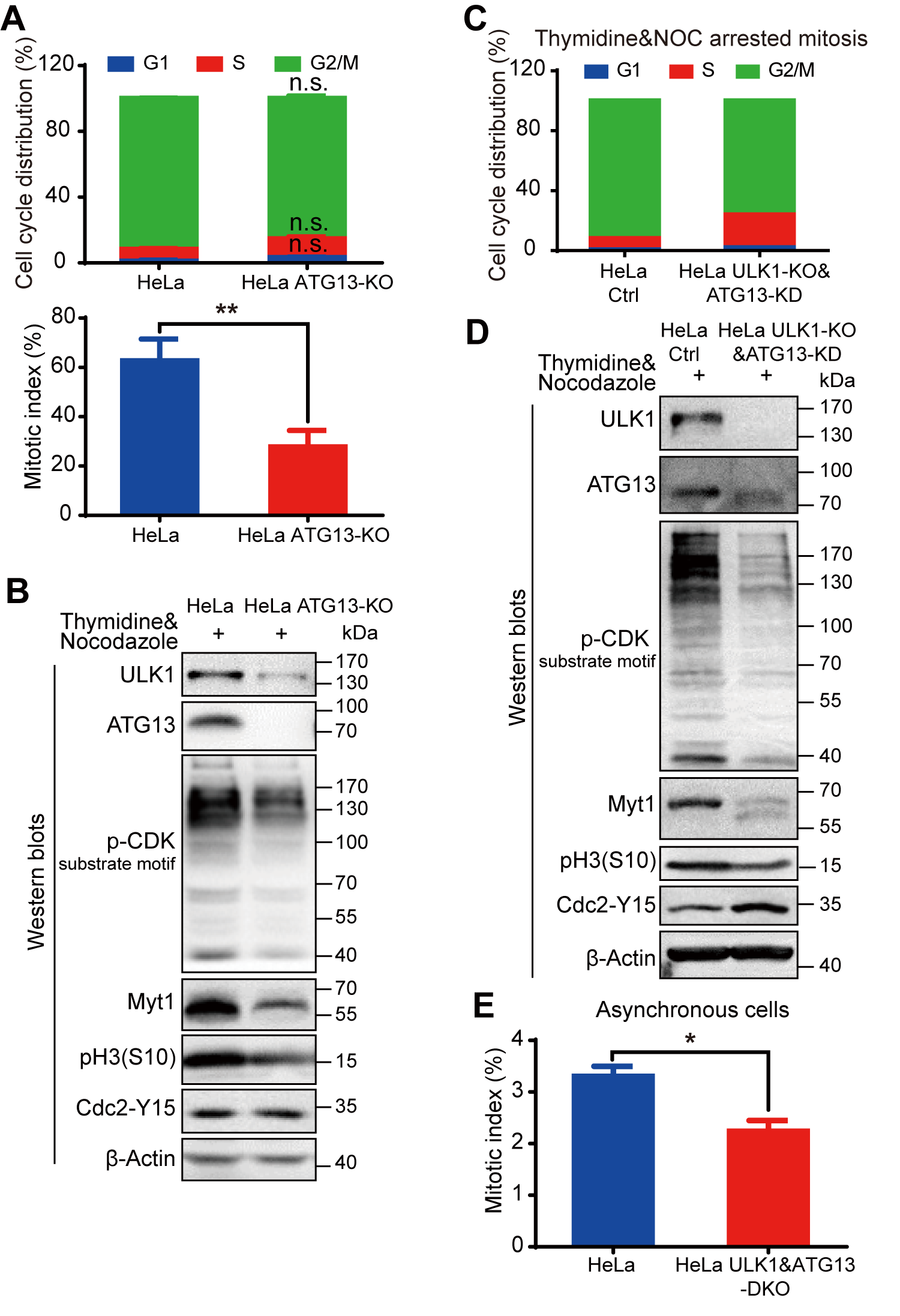

Supplement: S15 Fig — (A-B) Mitotic index was decreased in ATG13-KO cells synchronized by single-thymidine and nocodazole. HeLa WT or ATG13-KO cells synchronized into mitosis were subjected to PI and pH3(S10) co-staining for cell cycle and mitotic index analysis by flow cytometry (A) or western blot analysis for cell cycle markers (B). (C) ULK1-KO combined with ATG13 “knockdown” inhibits S/G2 transition. HeLa ULK1-KO cells transiently transfected with the CRISPR/Cas9 vector control or plasmid subcloned gRNA for human ATG13 were synchronized with thymidine and nocodazole for cell cycle analysis by flow cytometry. (D) The cell lysate collected from (C) was subjected to western blot analysis by indicated antibodies. (E) ULK1 and ATG13 DKO decreases mitotic index. HeLa WT or ULK1&ATG13-DKO cells were subjected to pH3(S10) staining for mitotic index analysis by flow cytometry. n = 3, *p < 0.05, **p < 0.01. Numerical data underlying the figure panels are available in S1 Data. ATG, autophagy-related; DKO, double knockout; gRNA, guide RNA; KO, knockout; n.s., not significant; PI, propidium iodide; ULK1, unc-51-like autophagy activating kinase 1; WT, wild type. (TIF) [file pbio.3000288.s017.tif]

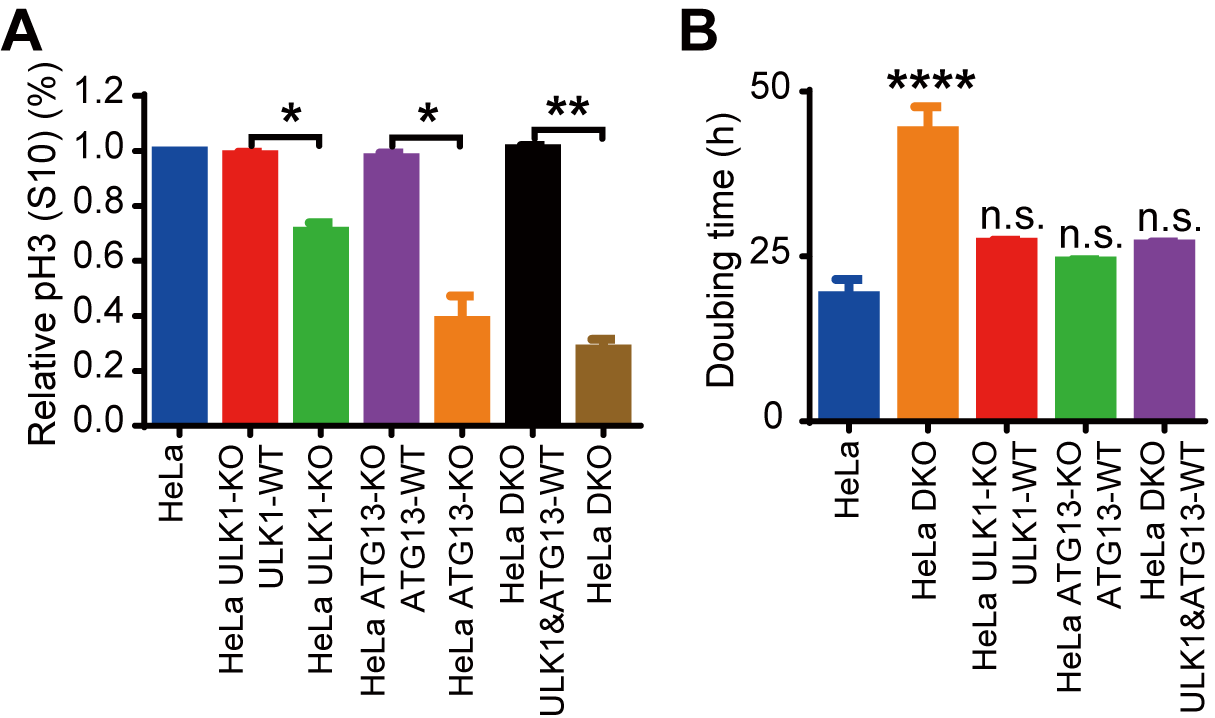

Supplement: S16 Fig — (A) Cells synchronized by thymidine and nocodazole were subjected to PI and pH3(S10) co-staining for cell cycle and mitotic index analysis by flow cytometry. (B) Doubling time of various cell lines. The statistical analysis (1-way ANOVA followed by Tukey’s multiple comparison test) was done by comparing indicated cells to HeLa cells. The doubling time is calculated as Fig 7D. n = 3, *p < 0.05, **p < 0.01, ****p < 0.0001. Numerical data underlying the figure panels are available in S1 Data. n.s., not significant; PI, propidium iodide. (TIF) [file pbio.3000288.s018.tif]

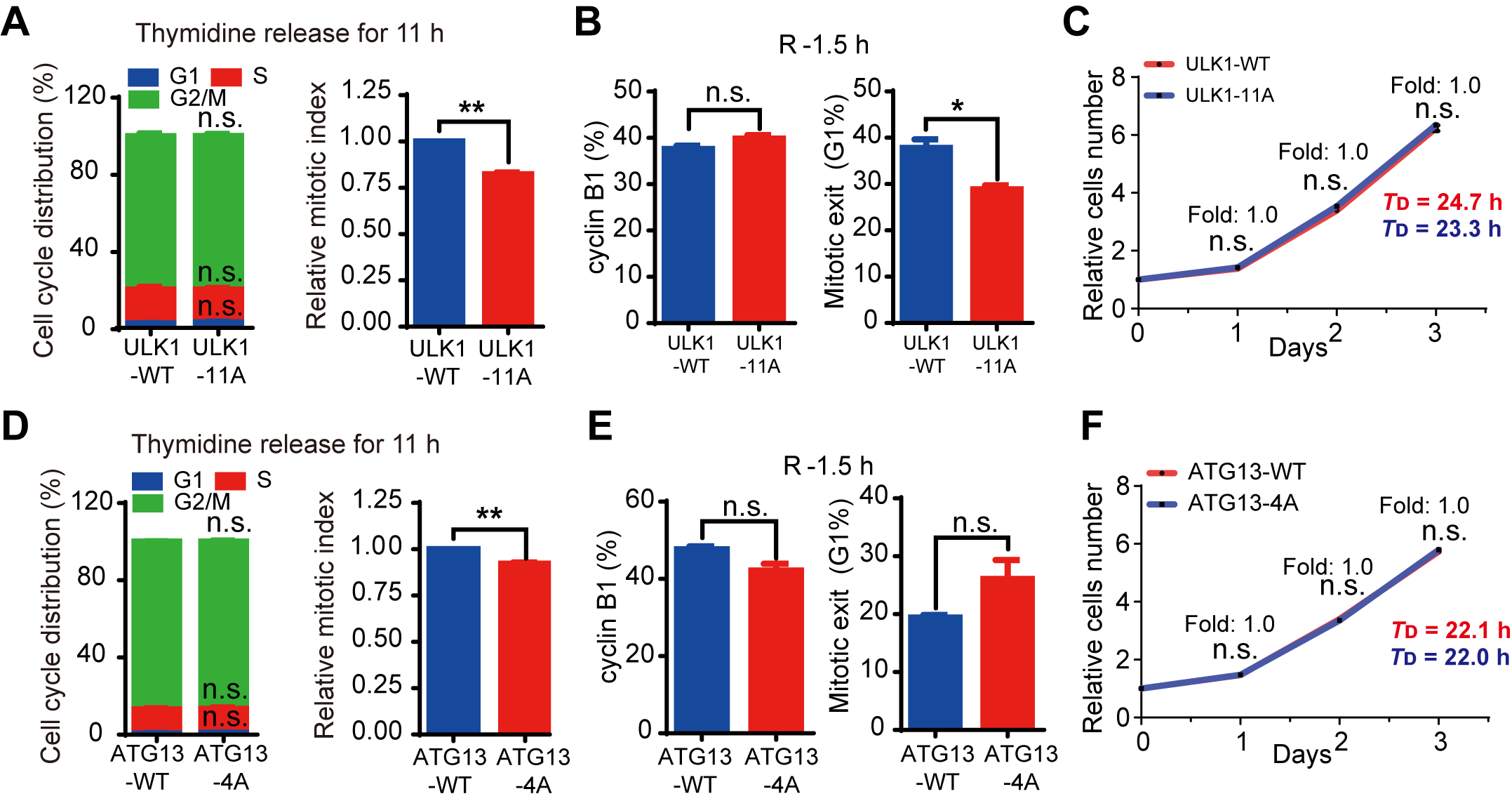

Supplement: S17 Fig — (A, D) The cell cycle distribution and mitotic entry of HeLa ULK1-KO cells stably overexpressing WT or mutant 11A FLAG-tagged mULK1 and HeLa ATG13-KO cells stably overexpressing WT or mutant 4A FLAG-tagged ATG13. Cells released from thymidine for 11 hours were subjected to PI and pH3(S10) co-staining for cell cycle and mitotic index analysis by flow cytometry. (B, E) Mitotic exit of ULK1 or ATG13 WT or mutant cells. Cells were synchronized into mitosis with thymidine and nocodazole and released into nocodazole-free complete DMEM medium for different time points and then subjected to either PI or cyclin B1 staining for cell cycle, and cyclin B1 level analysis by flow cytometry. (C, F) ULK1 or ATG13 WT or mutant does not affect cell proliferation. Cells were plated at 1 × 10^5^ cells/mL and cultured for 1, 2, or 3 days. The cell number was counted by flow cytometry, and the doubling time was calculated. TD indicates the average cell doubling time and is calculated as: TD = t*[lg2/(lgNt − lgN0)], where t is the culture time, Nt is the cell number after culturing, and N0 is the original cell number plated. n = 3, *p < 0.05, **p < 0.01. Numerical data underlying the figure panels are available in S1 Data. 11A, S622&T635&T653&S479&S543&S413&T401&S403&S405&T282&T502A; ATG, autophagy-related; DMEM, Dulbecco’s Modified Eagle Medium; KO, knockout; mULK1, mouse ULK1; n.s., not significant; PI, propidium iodide; ULK1, unc-51-like autophagy activating kinase 1; WT, wild type. (TIF) [file pbio.3000288.s019.tif]

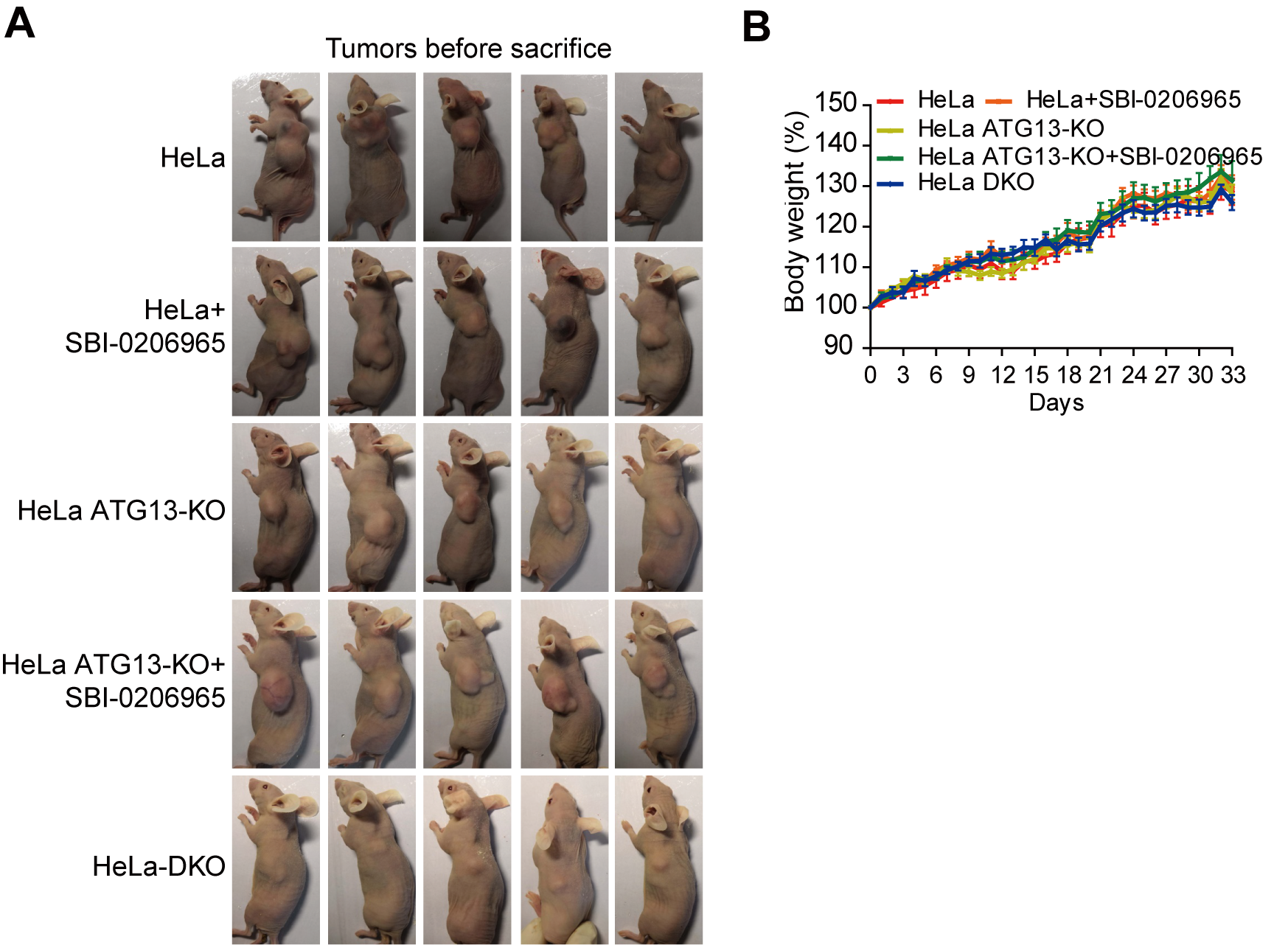

Supplement: S18 Fig — (A) The mouse model in nude mice bearing WT or KO cells treated with or without ULK1 kinase inhibitor SBI-0206965 was established. (B) Time course of the body weight for nude mice in (A). Numerical data underlying the figure panels are available in S1 Data. KO, knockout; ULK1, unc-51-like autophagy activating kinase 1; WT, wild type. (TIF) [file pbio.3000288.s020.tif]
